# Catabolism of Branched Chain Amino Acids Contributes Significantly to Synthesis of Odd-Chain and Even-Chain Fatty Acids in 3T3-L1 Adipocytes

**DOI:** 10.1371/journal.pone.0145850

**Published:** 2015-12-28

**Authors:** Scott B. Crown, Nicholas Marze, Maciek R. Antoniewicz

**Affiliations:** Department of Chemical and Biomolecular Engineering, Metabolic Engineering and Systems Biology Laboratory, University of Delaware, Newark, Delaware, United States of America; Case Western Reserve University, UNITED STATES

## Abstract

The branched chain amino acids (BCAA) valine, leucine and isoleucine have been implicated in a number of diseases including obesity, insulin resistance, and type 2 diabetes mellitus, although the mechanisms are still poorly understood. Adipose tissue plays an important role in BCAA homeostasis by actively metabolizing circulating BCAA. In this work, we have investigated the link between BCAA catabolism and fatty acid synthesis in 3T3-L1 adipocytes using parallel ^13^C-labeling experiments, mass spectrometry and model-based isotopomer data analysis. Specifically, we performed parallel labeling experiments with four fully ^13^C-labeled tracers, [U-^13^C]valine, [U-^13^C]leucine, [U-^13^C]isoleucine and [U-^13^C]glutamine. We measured mass isotopomer distributions of fatty acids and intracellular metabolites by GC-MS and analyzed the data using the isotopomer spectral analysis (ISA) framework. We demonstrate that 3T3-L1 adipocytes accumulate significant amounts of even chain length (C14:0, C16:0 and C18:0) and odd chain length (C15:0 and C17:0) fatty acids under standard cell culture conditions. Using a novel GC-MS method, we demonstrate that propionyl-CoA acts as the primer on fatty acid synthase for the production of odd chain fatty acids. BCAA contributed significantly to the production of all fatty acids. Leucine and isoleucine contributed at least 25% to lipogenic acetyl-CoA pool, and valine and isoleucine contributed 100% to lipogenic propionyl-CoA pool. Our results further suggest that low activity of methylmalonyl-CoA mutase and mass action kinetics of propionyl-CoA on fatty acid synthase result in high rates of odd chain fatty acid synthesis in 3T3-L1 cells. Overall, this work provides important new insights into the connection between BCAA catabolism and fatty acid synthesis in adipocytes and underscores the high capacity of adipocytes for metabolizing BCAA.

## Introduction

The branched chain amino acids (BCAA) valine, leucine and isoleucine are essential amino acids that comprise about 15–25% of total protein intake [1]. The first step of BCAA catabolism is the conversion of BCAA to their respective α-keto acids through mitochondrial branched chain aminotransferase (BCATm). Hepatic activity of this initial enzymatic step is very low [2], although the liver can readily oxidize the α-keto acids formed from BCATm activity in other peripheral tissues, such as skeletal muscle, cardiac muscle and adipose tissue. The rate-limiting step of BCAA catabolism is branch chain keto-acid dehydrogenase (BCKDH), which can be found in a host of tissues including liver, skeletal muscle, kidney, and adipose tissue. The major tissue responsible for BCAA oxidation is believed to be skeletal muscle [3]; however, the role of other tissues in BCAA metabolism, especially in states of metabolic disease are not well understood.

Due to poor catabolism of BCAA in the liver, BCAA can circulate through the bloodstream and act as signaling molecules of the nutritional state. BCAA, especially leucine, have been implicated as important sensor molecules that interact with the cell survival signaling pathway, i.e. mammalian target of rapamycin (mTOR), and the insulin signaling cascade [1,4,5]. Elevated plasma concentrations of BCAA and BCAA metabolism byproducts, e.g. alanine, glutamate/glutamine, and C3/C5-carnitine species, have been identified as a metabolic signature of obese individuals compared to their lean counterparts [6]. In addition, several cohort studies have demonstrated that elevated circulating concentrations of BCAA may even serve as biomarkers for predicting the development of insulin-resistance and T2DM [7–10]. Furthermore, there is evidence that BCAA levels associate with the success of weight-interventions either through lifestyle changes [8] or bariatric surgery [11,12].

Adipose tissue, an often underappreciated endocrine organ and regulator of energy homeostasis [13,14], may play a crucial role in amino acid regulation through modulation of circulating BCAA levels [15]. Disruption of adipose-specific catabolism of BCAA has been observed in obese and insulin-resistant states and correlated with elevated circulating BCAA concentrations [15–17]. It is hypothesized that changes in adipose BCAA oxidation plays an important role in the incidence and progression of these metabolic diseases [18].

With the important role adipose plays in BCAA metabolism, it is of interest to examine the interplay between BCAA catabolism and glucose metabolism. 3T3-L1 adipocytes are an important *in vitro* model system for adipose tissue and are commonly used as this cell line has similar phenotypic behavior as adipose tissue. It has been well documented that BCAA catabolic enzyme activities and expression levels greatly increase over the course of 3T3-L1 differentiation [19–21]. In addition, agonism of PPARγ, a key receptor in glucose and fatty acid metabolism, results in increased transcript levels of BCAA catabolic enzymes [22]. Furthermore, glucose and BCAA catabolism are coupled in 3T3-L1 adipocytes as inhibition of glucose metabolism with 2-deoxy-D-glucose resulted in decreased BCAA catabolic enzyme activity and expression, regardless of insulin supplementation [22].

In this work, we have quantified for the first time the contribution of BCAA to fatty acid synthesis in 3T3-L1 adipocytes. To this end, parallel isotopic labeling experiments were performed with four ^13^C-tracers: [U-^13^C]valine, [U-^13^C]leucine, [U-^13^C]isoleucine and [U-^13^C]glutamine. While conducting this work, we noted a significant accumulation of odd chain length fatty acids in 3T3-L1 adipocytes. With the help of a novel GC-MS analysis technique we systematically elucidated the pathway of odd chain fatty acid synthesis. Overall, this work represents an important advance towards understanding the interconnection between BCAA and fatty acid metabolism in adipocytes. Moreover, this study demonstrates the power of using parallel ^13^C-labeling experiments for pathway quantification and highlights a novel GC-MS methodology for fatty acid analysis.

## Materials and Methods

### Materials

Culture materials were purchased from Cellgro (Mediatech, Manassas, VA). [U-^13^C]Valine (99 atom% ^13^C), [U-^13^C]leucine (98%), [U-^13^C,^15^N]isoleucine (99% ^13^C), [U-^13^C]glutamine (98% ^13^C) and [U-^13^C]algal amino acids (97+% ^13^C) were purchased from Sigma-Aldrich (St. Louis, MO). Free amino acids were purchased from Fisher Sci. (Hampton, NH). Amino acid standard H was purchased from Pierce Sci. (Rockford, IL). Stock solutions (40x) of natural and uniformly ^13^C-labeled valine, leucine, and isoleucine were prepared at 40mM in deionized water. Stock solutions (40x) of natural and [U-^13^C]glutamine were prepared at 160mM in deionized water. [U-^13^C]algal amino acids were solubilized at 10mg/mL in 0.1N HCl phosphate buffered saline. All amino acid solutions were stored at -85°C until use.

### Culture and differentiation of 3T3-L1 adipocytes

**Fig 1:**
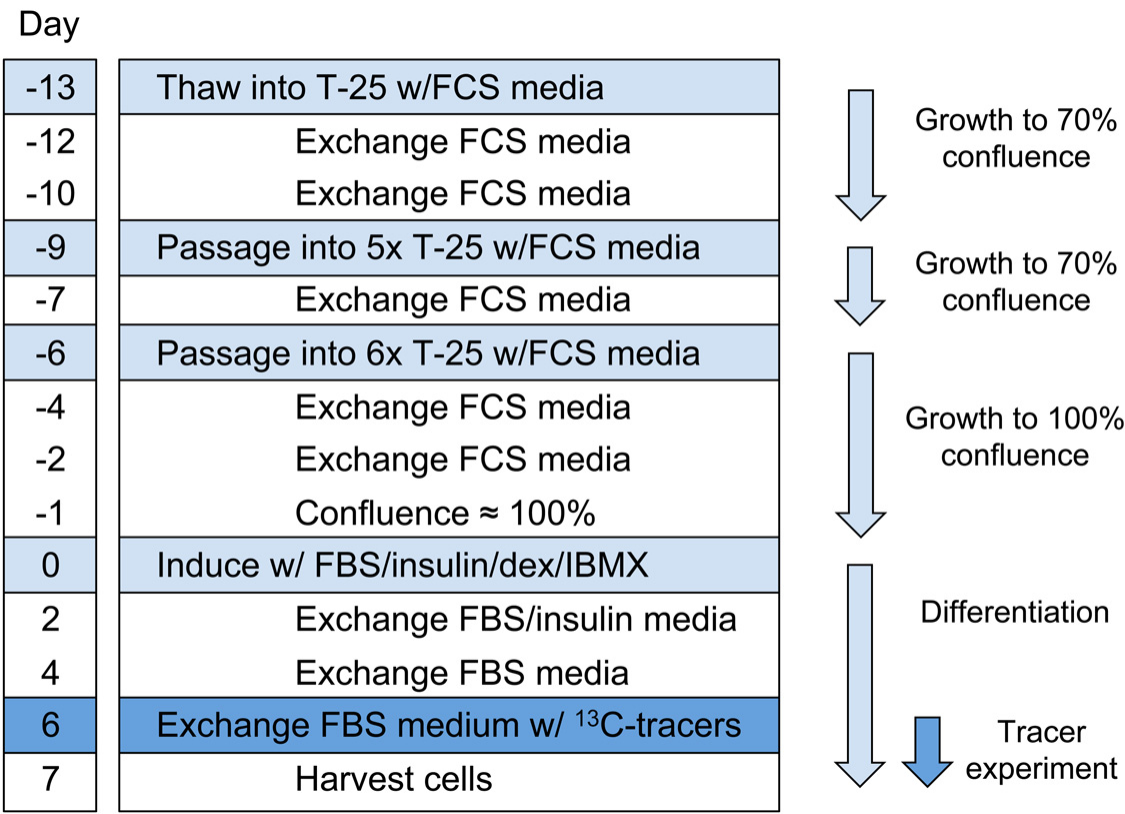
Timeline for ^13^C-tracer experiments with 3T3-L1 adipocytes.

Pre-adipocyte 3T3-L1 cells (ATCC, Cat. No. CL-173) were cultured in Dulbecco’s modified Eagle medium (DMEM, Cellgro, Cat. No. 10–013) supplemented with 10% fetal calf serum (FCS, Sigma-Aldrich, Cat. No. N4762) and 1% penicillin-streptomycin solution (PS) in T-25 CellBIND flasks (Corning, NY). Cells were grown in a humidified incubator at 37°C with 10% CO_2_ and passaged every three days (~70% confluence) at a split ratio of 1:6. Medium was exchanged with fresh medium every two days (Fig 1). To prepare for differentiation, preadipocytes were grown to 100% confluence and maintained at confluency for one day (designated as day 0). The monolayers were then incubated for 48 hours in DMEM with 10% fetal bovine serum (FBS, Cellgro, Cat. No. 35-011-CV), 1% PS and a differentiation cocktail that included 1μg/mL insulin, 1μM dexamethasone (dex) and 0.5mM 3-isobutyl-1-methylxanthine (IBMX). After 48 hours (day 2), medium was exchanged with DMEM/FBS/PS medium supplemented with 1μg/mL insulin. On day 4, medium was exchanged with DMEM/FBS/PS medium without any supplementations. On day 6, medium was exchanged with glutamine-free DMEM medium (Cellgro, Cat. No. 15–013) supplemented with 10% FBS, 1% PS, and additions of valine, leucine, isoleucine and glutamine from the 40x stock solutions. For parallel labeling experiments, one of the four amino acids was uniformly ^13^C-labeled and the other amino acids were unlabeled. After 24 hours of incubation (day 7), cells were harvested for metabolite extraction as described below.

### Cell visualization and staining

The differentiating adipocytes were observed by phase contrast imaging using a Leica DMI6000 B inverted microscope. Fatty acid visualization was conducted with Oil Red O (Sigma-Aldrich, Cat. No. O-0625) staining. To stain the cells, cells were washed twice with PBS, followed by fixation with a 10% formalin solution. After two washes with 60% isopropanol, cells were stained with 0.3% (w/v) Oil Red O in 60% isopropanol. Upon removal of Oil Red O, cells were washed twice with PBS and visualized using a Leica DMI6000 B inverted microscope.

### Isotopic tracer experiments

For isotopic tracer experiments, one vial of frozen 3T3-L1 cells was thawed and plated in a T-25 flask. After four days, the cells were split into fresh T-25 flasks (passage 1), grown for three days, and split again at a split ratio of 1:6 (passage 2) (Fig 1). This resulted in a total of 26 flasks for cell culture experiments, where all cells were derived from the same frozen vial.

Four stable isotope tracers were used in the study: 1) [U-^13^C]valine (MPE ~45%); 2) [U-^13^C]leucine (MPE ~42%); 3) [U-^13^C,^15^N]isoleucine (MPE ~54%); 4) [U-^13^C]glutamine (MPE ~97%). The molar percent enrichment (MPE) of the BCAA tracers is less than 100%, since the BCAA tracers were added in addition to the ~0.8mM already present in DMEM media. All isotopic tracer experiments were conducted with four biological replicates (*n* = 4) where the only difference was which amino acid was labeled. In addition to the 16 flasks for the labeling experiments, six flasks were used for cell counting. Furthermore, four flasks without tracer addition were used for quantifying media metabolites and 500μL of media was removed at 0, 6, 12 and 24 hours. At the end of the labeling experiment (day 7), tracer experiment flasks were harvested and cells were extracted as described below to obtain intracellular metabolites for analysis of ^13^C-labeling by GC-MS. All samples were stored at -85°C prior to analysis.

### Viable cell number, glucose and lactate analysis

Cell numbers (n = 6) were measured using a hemocytomer and cell viability was determined by trypan blue exclusion method. For each sample, a minimum of 500 cells was counted. Concentrations of glucose and lactate were measured in triplicate by YSI 2700 biochemistry analyzer (YSI, Yellow Springs, OH).

### Gas chromatography-mass spectrometry

GC-MS analysis was performed using an Agilent 7890A GC equipped with a DB-5ms capillary column (30 m x 0.25 mm i.d. x 0.25μm; Agilent J&W Scientific) for analysis of aqueous metabolites and fatty acid picolinyl esters; and a DB-23 capillary column (30 m x 0.25 mm i.d. x 0.25μm; Agilent J&W Scientific) for analysis of fatty acid methyl esters (FAMEs) [23]. The GC instrumented interfaced with a Waters Quatro Micro GC-MS/MS (Milford, MA) operating under electron impact ionization at 70 eV and 200°C ion source temperature. The injection port and interface temperatures were 250°C for the DB-5ms column and 230°C for the DB-23 column. Helium flow was maintained at 1 mL/min. Mass spectra were recorded in selected ion recording (SIR) mode with 30 ms dwell time. Mass isotopomer distributions were obtained by integration of ion chromatograms [24] and corrected for natural isotope abundances [25].

### Extraction of intracellular metabolites

At the harvesting time, culture medium was collected from the T-25 flasks and stored at -85°C until further analysis. The cells were washed twice with a chilled 0.9% NaCl solution (4°C). Metabolic activity was quenched by the addition of 1.5 mL of cold methanol (-20°C) and cells were subsequently placed on ice. After five-minute incubation, cells were scraped off the flask surface and the cell suspension was transferred into glass tubes with Teflon-sealed caps. Next, 1.5 mL of chloroform was added and the tubes were vortexed vigorously for 10 s. Lastly, any remaining cell debris in the flasks was resuspended in 1.5 mL of water and transferred to the glass tubes. The tubes were vortexed for 1 minute and then stored overnight at 4°C. The following day, the tubes were centrifuged at 2000 rpm and 4°C for 20 minutes. The resulting phase separation resulted in an aqueous upper phase containing polar metabolites and an organic lower phase containing nonpolar metabolites. The upper phase (methanol and water) was transferred to two 1.5 mL Eppendorf tubes and the lower phase (chloroform) was transferred to a glass tube with a Teflon-sealed cap. Samples were stored at -85°C until further use.

### Derivatization and GC-MS analysis of extracellular metabolites

Immediately after sampling, 200 μL of medium was spiked with 30 μL of 10 mg/mL [U-^13^C]algal solution, 10 μL of 60 mM [U-^13^C]glutamine and 30 μL of 0.1N HCl [26]. Samples were then treated with 1 mL of cold acetone (-20°C) and vortexed vigorously. The deproteinized samples were centrifuged at 14,000g for 5 minutes and the supernatants were evaporated to dryness under N_2_ flow at 37°C using an evaporator (Reacti-Vap/Reacti-Therm III; Fierce, Rockford, IL). 50 μL of pyridine was added to the samples and mixed by pipetting. 50 μL of N-methyl-N-(tert-butyl-dimethylsilyl)-trifluoroacetamide (MTBSTFA) + 1% tert-butyldimethylchlorosilane (TBDMS) (Thermo Scientific, Bellefonte, PA) was added and the samples were derivatized at 60°C for 30 minutes. Afterwards, samples were incubated overnight at room temperature. The derivatized samples were centrifuged for 2 minutes at 14,000g and the supernatants were transferred to GC vials for GC-MS analysis. The injection volume was 1 μL and samples were injected in split mode with split ratio ranging from 1:10 to 1:20. GC oven temperature was held at 80°C for 2 minutes, increased to 280°C at 7°C/min and held until the total run time was 40 minutes. Mass spectra of selected metabolite fragments (Table B in S1 File) were collected in SIR mode.

### Derivatization and GC-MS analysis of intracellular metabolites

The extracted aqueous samples were thawed and evaporated to dryness at 37°C with N_2_ using an evaporator. During the process, the contents of the two Eppendorf tubes were combined. Metabolites were suspended in 50 μL of 2 wt% methoxylamine hydrochloride in pyridine and incubated at 37°C for 90 minutes on a heating block. Next, 70 μL of MTBSTFA + 1% TBDMS was added and the samples were incubated at 60°C for 30 minutes. Next, samples were incubated overnight at room temperature. The derivatized samples were centrifuged for 2 minutes at 14,000g and the supernatants were transferred to GC vials for GC-MS analysis. The injection volume was 1 μL and samples were injected in split mode with split ratio ranging from 1:2 to 1:5. GC oven temperature was held at 70°C for 2 minutes, increased to 140°C at 3°C/min, increased to 150°C at 1°C/min, increased to 242°C at 3°C/min, increased at 10°C/min to 280°C and held until the total run time was 75 minutes. Mass spectra of selected metabolite fragments (Table C in S1 File) were collected in SIR mode.

### Derivatization and GC-MS analysis of fatty acids

For analysis of fatty acid labeling, the chloroform phase was defrosted and split into two glass tubes. Both samples were then evaporated to dryness under air at 37°C using an evaporator. One sample was prepared as fatty acid methyl esters (FAMEs) to measure molecular ion mass spectra. The second sample was derivatized into picolinyl esters to obtain positional labeling information.

#### Fatty acid methyl ester (FAME) derivatization and GC-MS analysis

Dried lipids were resuspended in 1 mL of chloroform and 1 mL of methanol. Next, 100 μL of concentrated sulfuric acid was added to the samples. The glass tubes were wrapped with Teflon tape and sealed with Teflon-coated caps. After 2-hour incubation at 100°C, the samples were cooled to room temperature and 0.5 mL of deionized water was added. The resulting two-phase mixtures were vortexed vigorously for 1 minute and allowed to phase separate. To accelerate phase separation, samples were centrifuged at 2000 rpm and 4°C for 5 minutes. After a clear phase boundary formed, the lower phase (chloroform) was pipetted into a 1.5 mL Eppendorf tube and evaporated to dryness at 37°C under air using an evaporator. The samples were then resuspended in 200 μL of hexane and mixed by pipetting. To remove insoluble debris, samples were centrifuged at 14,000 rpm for 2 minutes. The clear liquid was transferred to a glass GC vial for GC-MS analysis. The injection volume was 1 μL and samples were injected in split mode with split ratio ranging from 1:4 to 1:10. GC oven temperature was held at 125°C for 2 minutes, increased to 240°C at 5°C/min, and held until the total run time was 30 minutes. Mass spectra of selected metabolite fragments (Table C in S1 File) were collected in SIR mode.

#### Picolinyl ester derivatization and GC-MS analysis

Picolinyl ester derivatization was modified from previous studies [27,28]. Dried lipids were resuspended in 1 mL dichloromethane. Next, 300 μL of a fresh 3-hydroxymethylpyridine and potassium tert-butoxide (in 1M THF) solution (2:1 v/v) was added to the samples. After 30-minute incubation at 40°C, the solutions were cooled to room temperature, 2 mL of water was added to the samples, followed by 4 mL of hexane. The resulting solution was vortexed vigorously. Once two phases were clearly formed, the upper phase (organic) was collected and passed through packed sodium sulfate bed to strip moisture. The anhydrous samples were then evaporated to dryness at 37°C under air using an evaporator. The samples were then resuspended in 100 μL of hexane and mixed by pipetting. To remove insoluble debris, samples were centrifuged at 14,000 rpm for 2 minutes. The clear liquid was transferred to a glass GC vial for GC-MS analysis. The injection volume was 1 μL and samples were injected in split mode with split ratio ranging from 1:5 to 1:10. GC oven temperature was held at 200°C for 10 minutes, increased to 210°C at 5°C/min, held for 5 minutes, increased to 280°C at 5°C/min and held until the total run time was 40 minutes. Mass spectra of selected metabolite fragments (Table C in S1 File) were collected in SIR mode.

### Determination of [U^-13^C]algal amino acid concentrations

To determine concentrations of amino acids in [U-^13^C]algal amino acid solution, 30μL of 10mg/mL [U-^13^C]algal amino acids was spiked with 0, 6, 12.5, 25, or 50 μL of amino acid standard H (1.25 μmol/mL for cystine, and 2.5 μmol/mL for other amino acids). Samples were dried under nitrogen, derivatized with TBDMS, and analyzed by GC-MS as described above. From the isotopic dilution of the mass spectra the concentrations of the amino acids in the [U-^13^C]algal solution were determined (Table B in S1 File) [29]. The [U-^13^C]algal amino acid solution was then used as an internal standard to quantify the concentrations of amino acids in medium samples.

### Isotopomer spectral analysis of fatty acids

Isotopomer spectral analysis (ISA) was conducted on the measured mass isotopomer distributions (MIDs) of C14:0, C15:0, C16:0, C17:0 and C18:0 fatty acids [30] to determine the fractional new synthesis of each fatty acid (g-values) and the relative contributions of glutamine, BCAA, and other metabolites to the precursor pools for fatty acid synthesis (D-values). Even chain length fatty acids were modeled as the condensation products of multiple acetyl-CoA (AcCoA) units. For example, C16:0 was modeled as the condensation product of 8 AcCoAs. Odd chain length fatty acids were modeled as the condensation product of a three-carbon precursor (propionyl-CoA, PropCoA) and multiple AcCoAs. For example, C15:0 was modeled as the condensation product of PropCoA and 6 AcCoAs. Metabolites contributing to lipogenic AcCoA were leucine, isoleucine, glutamine and other unlabeled metabolites (e.g. glucose); metabolites contributing to lipogenic PropCoA were valine and isoleucine (Tables AS, AT and BT in S1 File).

In addition to determining D- and g-values, we also determined the mass isotopomer distributions of AcCoA and PropCoA produced from ^13^C-glutamine and ^13^C-BCAA tracers. For example, rather than assuming that [U-^13^C]glutamine and [U-^13^C]-labeled BCAA would produce M+2 labeled AcCoA, in the ISA model we allowed each [U-^13^C]-labeled tracer to produce M+1 and M+2 AcCoA; similarly, [U-^13^C]valine and [U-^13^C]isoleucine were allowed to produce M+1, M+2 and M+3 labeled PropCoA (Tables AS, AT and BT is S1 File). The relative abundances of M+1, M+2 and M+3 mass isotopomers were estimated together with the other ISA model parameters using the Metran software [31], which is based on the EMU framework [32]. In short, ISA model parameters were estimated by minimizing the variance-weighted sum of squared residuals (SSR) between the experimentally measured and model predicted mass isotopomers of fatty acids using non-linear least-squares regression [33]. For each fatty acid, the MIDs from the four parallel tracer experiments with [U-^13^C]glutamine, [U-^13^C]valine, [U-^13^C]leucine and [U-^13^C]isoleucine tracers were fitted simultaneously. Methods for parallel fitting of multiple data sets with the Metran software have been described before [34–36]. In order to find a global solution, flux estimation was repeated at least 10 times starting with random initial values for all fluxes. The fitted results were subjected to a χ^2^ statistical test to assess the goodness-of-fit, and accurate 95% confidence intervals were computed for all estimated parameters by evaluating the SSR sensitivity to flux variations [37,38].

## Results

### Cell growth and differentiation of adipocytes

3T3-L1 cells were cultured from the pre-adipocyte state to confluence and 24 hours post confluence (designated day 0), the cells were chemically differentiated with insulin, dexamethasone and IBMX to achieve the mature adipocyte phenotype (Fig 1). Undifferentiated preadipocyte cells, counted on day 0, had a viable cell density of 1.28 ± 0.03 ×10^6^ cells/mL and viability of 95 ± 1% (Table A in S1 File).

**Fig 2:**
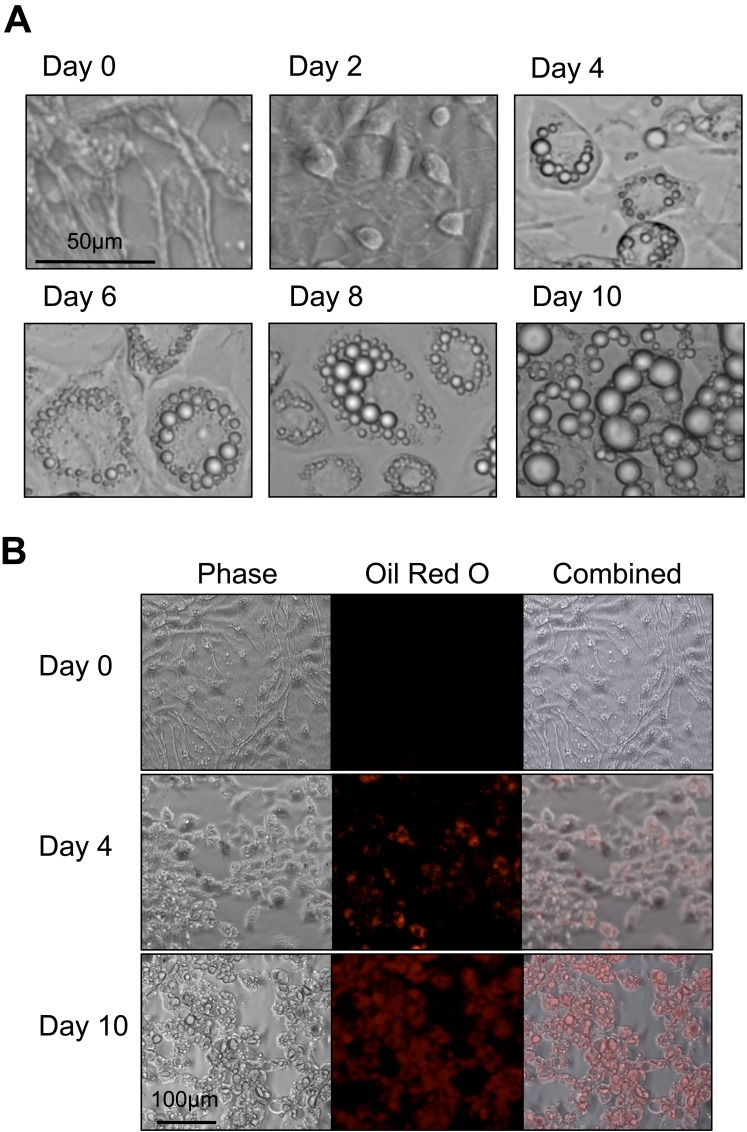
3T3-L1 adipocyte differentiation. (A) Phase contrast images of 3T3-L1 cells from induction (day 0) to ten days post-induction. Cells initially display a fibroblast phenotype. Over the course of differentiation, cell morphology changes and cells accumulate lipid droplets internally. (B) Triglyceride staining of 3T3-L1 cells with Oil Red O. Initially, the fibroblast phenotype displays negligible staining. As the adipocytes mature, the amount of staining increases until almost the entire cell volume is stained red.

Before differentiation, the cells displayed a fibroblast phenotype (Fig 2A), whereas after 48 hours of the differentiation process (day 2), the cells were more rounded and compact. On day 4, small droplets of triglyercides were visible in the periphery of the cells and over the remaining duration until day 10, triglyceride droplet accumulation increased until almost the entire cell volume was comprised of lipid droplets. Cells were treated with Oil Red O to confirm triglyceride accumulation (Fig 2B). On day 0, no visible staining of lipids was detected. After four days, triglyceride accumulation was detected in small amounts, especially in the periphery of cells. On day 10, almost the entire intracellular volume was stained, indicating significant accumulation of triglycerides. For ^13^C-tracer experiments, it was desirable to have noticeable accumulation of triglycerides over the duration of the tracer experiment. We determined that day 6 would be ideal for isotopic experiments, since the adipocyte phenotype has already been developed by that time [39–41], and intracellular triglyceride accumulation has not yet saturated.

### Glucose, lactate and glutamine metabolism

**Fig 3:**
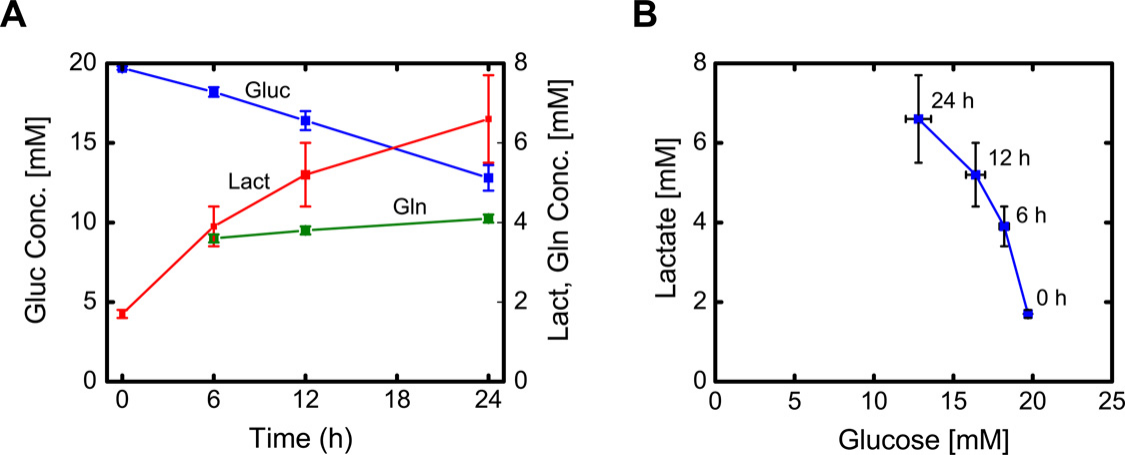
(A) Glucose, lactate, and glutamine concentration profiles over the course of the tracer experiment between days 6 and 7 (mean ± stdev; *n* = 4 biological replicates). (B) Profile of lactate concentration versus glucose concentration. The yield of lactate on glucose decreased during the experiment as indicated by a change in the slope.

Extracellular glucose, lactate and glutamine concentration profiles between days 6 and 7 are shown in Fig 3A. The initial glucose concentration was ~20 mM. Glucose was consumed at a constant rate of 227 nmol/10^6^ cells/h. The initial lactate concentration was 1.7 mM. Lactate was initially produced at a relatively high yield on glucose of ~1.4 mol/mol, but this decreased to ~0.4mol/mol after 24 hours (Fig 3B). Since glucose consumption remained constant over the 24-hour period, the decreased lactate yield indicates a shift in pyruvate metabolism. The initial glutamine concentration was ~3.5 mM and increased to 4.1 mM. Glutamine was produced at a tenth of the rate of glucose uptake (23 nmol/10^6^ cells/h). Despite the net increase in glutamine concentration, glutamine was highly exchanged between the medium and the cells. For [U-^13^C]glutamine experiments, medium glutamine was initially 100% M+5 labeled; however after 24 hours glutamine was only about 60% M+5 labeled (Figure F in S1 File). The decrease in M+5 labeling could not be explained solely by net glutamine secretion, as that would have resulted in ~85% M+5 (= 3.5mM /4.1mM).

### Amino acid metabolism

**Fig 4:**
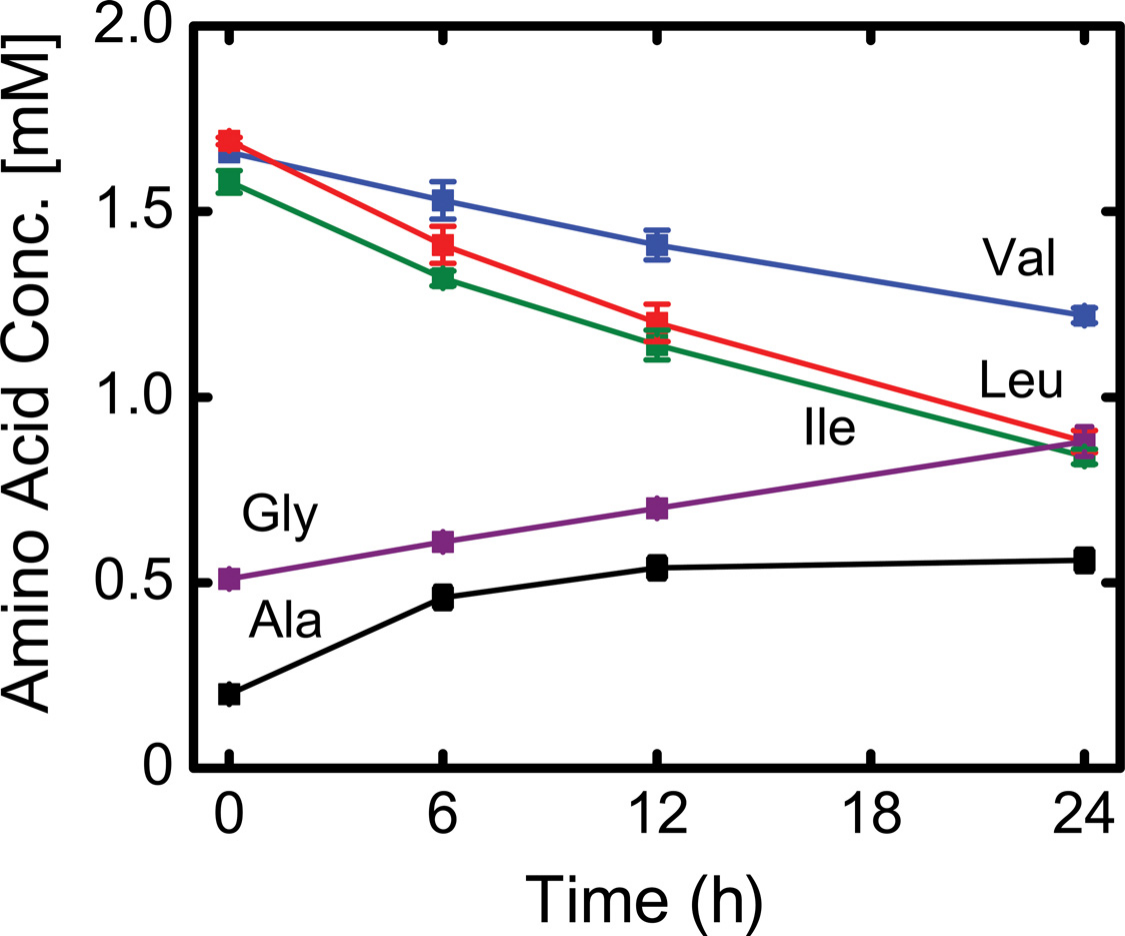
Amino acid concentration profiles over the course of the tracer experiment between days 6 and 7 (mean ± stdev; *n* = 4 biological replicates). Valine, leucine and isoleucine were consumed at a high rate, while glycine and alanine were secreted by the cells.

Several other amino acids were also actively net consumed (or produced) by the cells (Fig 4 and Table 1). Significantly, the cells readily consumed the BCAAs valine, leucine, and isoleucine. Isoleucine and leucine were consumed at comparable rates, 25 nmol/10^6^ cells/h, whereas valine uptake rate was 14 nmol/10^6^ cells/h. The BCAA consumption rates were several-fold greater than the consumption rates of other amino acids. Other than glutamine, the major secreted amino acids were alanine and glycine (Fig 4). Secretion of alanine and glycine is commonly observed in mammalian cell cultures as these amino acids can serve as a nitrogen sink [42].

**Table 1:** Glucose, lactate and amino acid production and consumption rates (nmol/10^6^ cells/h, mean ± SEM, *n* = 4 biological replicates).

Metabolite	Rate
Glucose	-227 ± 6
Lactate	153 ± 31
Alanine	10.4 ± 5.1
Glycine	12.0 ± 0.2
Valine	-14.2 ± 0.8
Leucine	-25.8 ± 2.5
Isoleucine	-23.5 ± 2.2
Methionine	-0.7 ± 0.1
Serine	-2.2 ± 0.4
Threonine	1.4 ± 0.8
Phenylalanine	1.0 ± 0.1
Aspartate	-1.2 ± 0.6
Glutamate	-2.3 ± 1.2
Tyrosine	0.6 ± 0.1
Glutamine	23.2 ± 0.8

Negative values denote net consumption.

### Fatty acid profiling

**Fig 5:**
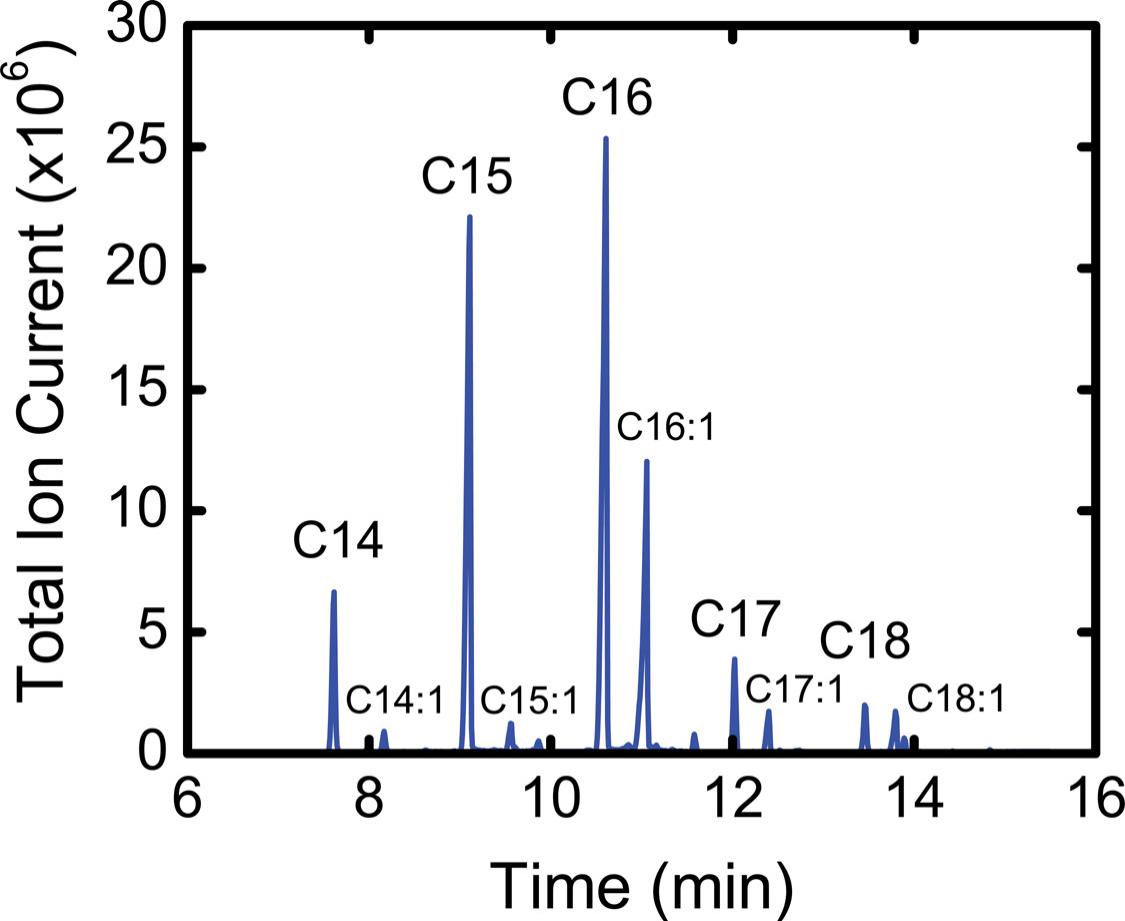
Total ion chromatogram from GC-MS analysis of fatty acid methyl esters (FAMEs). Fatty acids were extracted from differentiated 3T3-L1 adipocytes. Selected ion recording of molecular ions was conducted for GC-MS analysis.

Intracellular fatty acids were assayed by GC-MS after extraction of cellular triglycerides and subsequent transesterification with methanol. A representative total ion chromatogram for fatty acid methyl esters (FAMEs) is shown in Fig 5. Both even and odd chain fatty acids were detected, ranging in chain length from C14 to C18.

### Link between BCAA catabolism and fatty acid synthesis

**Fig 6:**
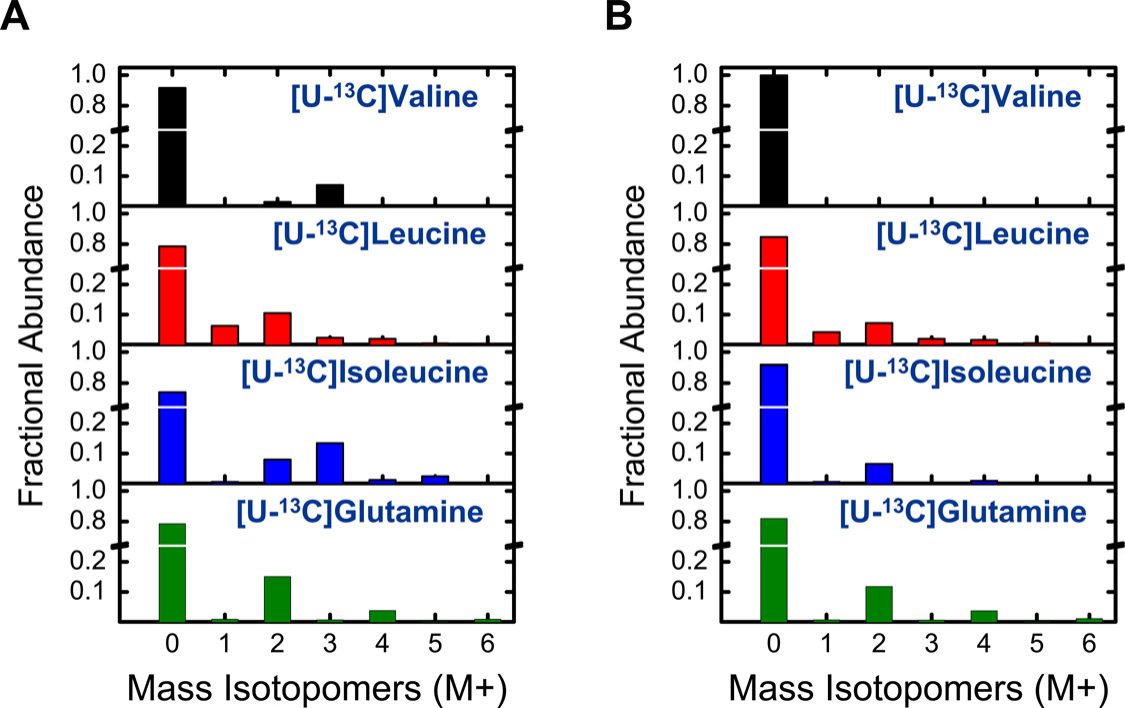
Mass isotopomer distributions of (A) methyl pentadecanoate (C15:0) and (B) methyl palmitate (C16:0) for four parallel labeling experiments with [U-^13^C]valine, [U-^13^C]leucine, [U-^13^C]isoleucine and [U-^13^C]glutamine tracers.

To investigate a potential link between BCAA catabolism and fatty acid synthesis, experiments were conducted with [U-^13^C]valine, [U-^13^C]leucine, [U-^13^C]isoleucine, and [U-^13^C]glutamine as tracers. Mass isotopomer distributions of FAMEs of pentadecanoic (C15:0) and palmitic (C16:0) acid are shown in Fig 6. Comparing the spectra for C15:0 and C16:0 we noted that: 1) for [U-^13^C]valine, C15:0 had a significant M+3 isotopomer, whereas C16:0 did not; 2) for [U-^13^C]isoleucine, C15:0 had a significant M+3 isotopomer and C16:0 did not; both fatty acids had M+2 isotopomers; 3) for [U-^13^C]leucine, both fatty acids had M+1 and M+2 isotopomers; 4) for [U-^13^C]glutamine, both fatty acids had M+2 and M+4 isotopomers.

Catabolism of valine and isoleucine is known to produce propionyl-CoA (PropCoA), which can account for M+3 isotopomers in [U-^13^C]valine and [U-^13^C]isoleucine experiments. As expected, palmitic acid did not show M+3 incorporation, since even chain fatty acids are formed solely from condensation of AcCoA. Isoleucine degradation also contributes to AcCoA, which explains the M+2 isotopomers in [U-^13^C]isoleucine experiments. Leucine degrades only into AcCoA, which accounts for M+1 and M+2 isotopomers in the [U-^13^C]leucine experiment. Details regarding the formation of M+1 isotopomers from [U-^13^C]leucine are discussed in the next section. Glutamine also contributes to lipogenic AcCoA pool, which explains the M+2 and M+4 isotopomers for [U-^13^C]glutamine experiments.

From these experiments, it can be concluded that valine and isoleucine both contribute to odd chain fatty acid synthesis through PropCoA precursor, and that PropCoA is not involved in the synthesis of even chain fatty acids. Furthermore, isoleucine, leucine, and glutamine all contribute to lipogenic AcCoA precursor pool.

### Odd chain fatty acid synthesis pathway

**Fig 7:**
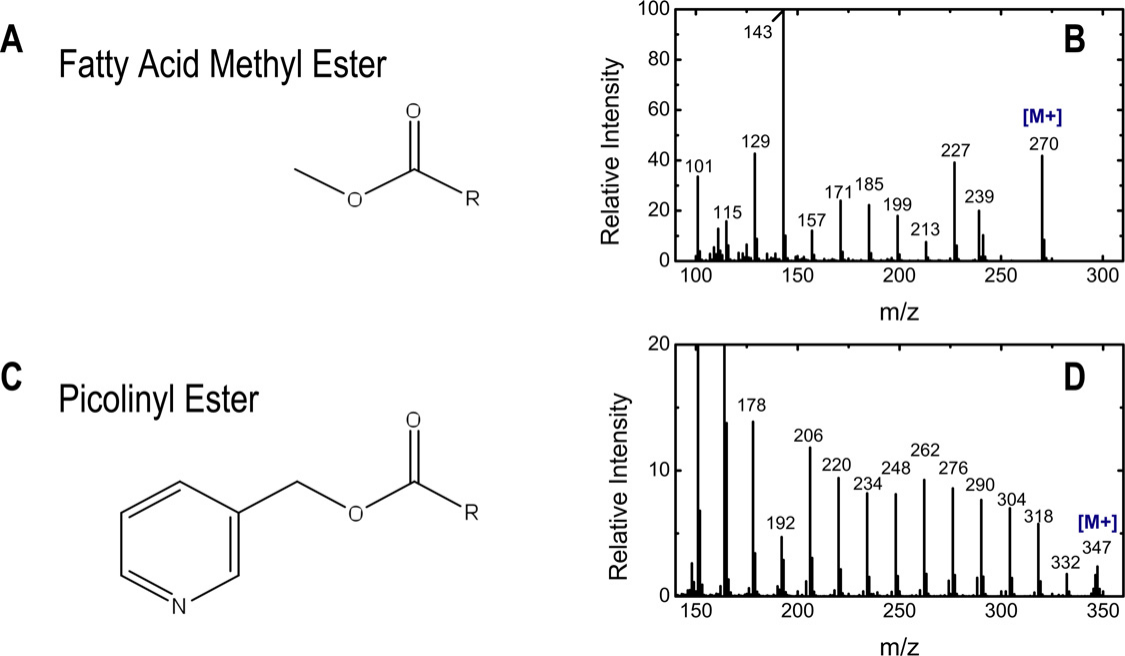
GC-MS analysis of fatty acid picolinyl ester derivatives. (A) Chemical structure of fatty acid methyl ester derivatives. (B) Mass spectrum of palmitic acid (C16:0) methyl ester derivative. Intermediate mass fragments cannot be used for positional labeling information as they are not unique fragments. (C) Chemical structure of fatty acid picolinyl ester derivatives. (D) Mass spectrum of palmitic acid (C16:0) picolinyl ester derivative. The dominant fragments in the spectrum are separated by 14 amu corresponding to a unique loss of consecutive CH_2_ groups.

To investigate where PropCoA is added to odd chain fatty acid carbon backbone, two fatty acid derivatizations were used in this study to gain structural information. FAMEs were used to determine the labeling of the complete molecule and picolinyl esters were utilized for investigating fragments of shorter carbon length. The structure of picolinyl esters and a representative mass spectrum of the palmitate derivative are shown in Fig 7. Mass spectra of picolinyl esters are characterized by fragments that are separated by 14 amu (e.g. *m/z* 332,318, 304, etc. for the palmitate derivative), corresponding to subsequent removal of -CH_2_ subunit from the ω-end of the fatty acid.

**Fig 8:**
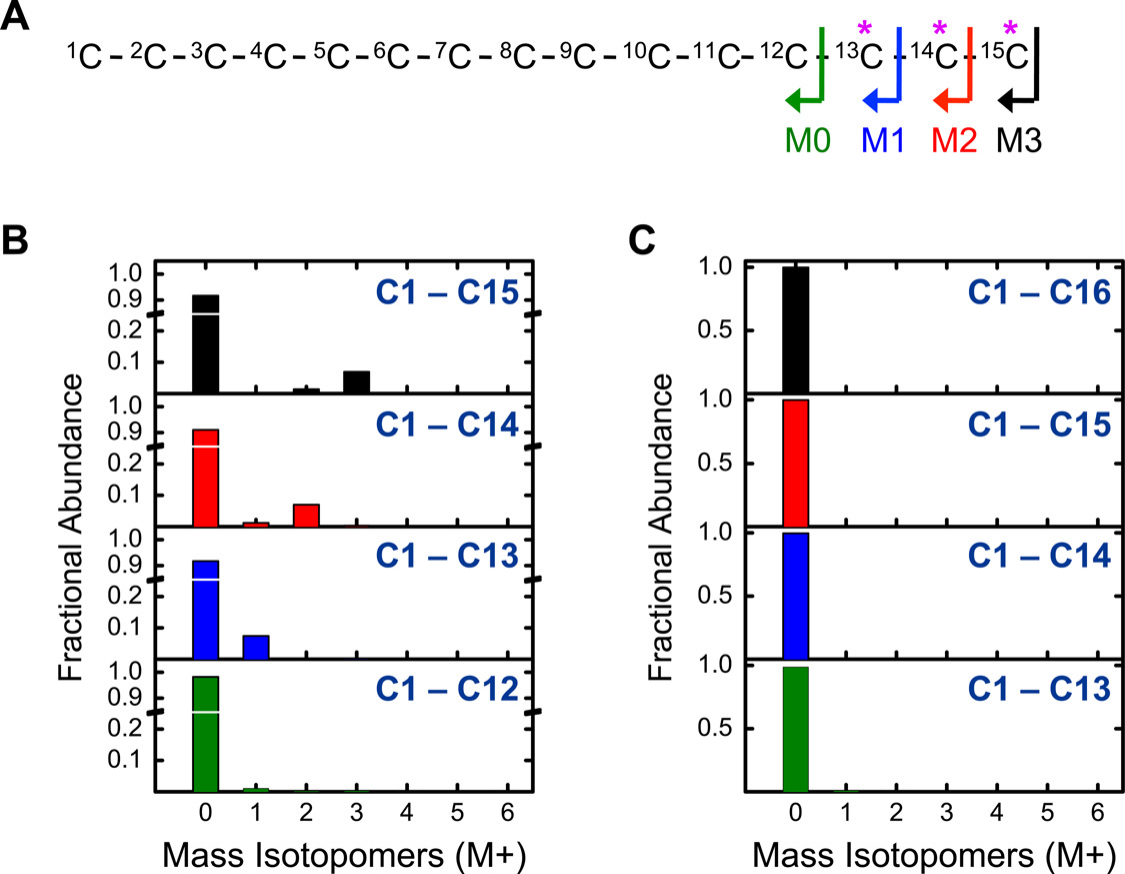
(A) Fragments of pentadecanoatic acid (C15:0) measured by GC-MS. An asterisk (*) indicates the expected location of ^13^C-labeled carbon atoms from [U-^13^C]valine. (B) Mass isotopomer distributions for four fragments of C15:0 retaining different parts of the fatty acid carbon backbone (from [U-^13^C]valine experiment). (C) Mass isotopomer distributions measured for four fragments of C16:0 from [U-^13^C]valine experiment.

For odd chain fatty synthesis it is assumed that PropCoA acts as the primer on fatty acid synthase that is subsequently elongated by AcCoA units. For [U-^13^C]valine, this would result in pentadecanoic acid being labeled on the first three carbons from the ω-end, i.e. carbon atoms C13-15. By applying the picolinyl ester derivatization for the [U-^13^C]valine experiment, this hypothesis was tested by measuring labeling of fragments corresponding to the removal of carbon atoms C13, C14 and C15 from pentadecanoic acid (Fig 8A). For the entire molecule (i.e. the FAME derivative) M+3 labeling was observed. Cleavage of the last carbon atom resulted in M+2 labeling, cleavage of the last two carbons resulted in M+1 labeling, and cleavage of the last three carbons resulted in an unlabeled fragment (Fig 8). The mass spectrum for pentadecanoic acid thus followed the expected trend. For palmitic acid, the fatty acid was unlabeled (M+0) for each fragment as was anticipated given that even chain fatty acids are produced from the elongation of AcCoAs. These data clearly show that PropCoA acts as the primer on fatty acid synthase for odd chain fatty acid synthesis.

### Quantifying BCAA contribution to fatty acid synthesis

Based on the results described in the previous section, a simplified model for the synthesis of odd chain and even chain fatty acids was constructed (Tables AS and AT in S1 File). Even chain fatty acids, such as palmitic acid (C16:0), were modeled as condensation products of AcCoA units. Potential sources for AcCoA were glutamine, leucine, isoleucine, and others (e.g. glucose). Odd chain fatty acids, such as pentadecanoic acid (C15:0), were modeled as products of the elongation of a single PropCoA moiety with AcCoA units. Sources of AcCoA were the same as for even chain fatty acids, whereas valine and isoleucine contributed to PropCoA. Isotopomer spectral analysis (ISA) was applied to determine the fractional contributions (D-values) of the various precursors to AcCoA and PropCoA, as well as fractional new synthesis (g-values) of each fatty acid.

**Fig 9:**
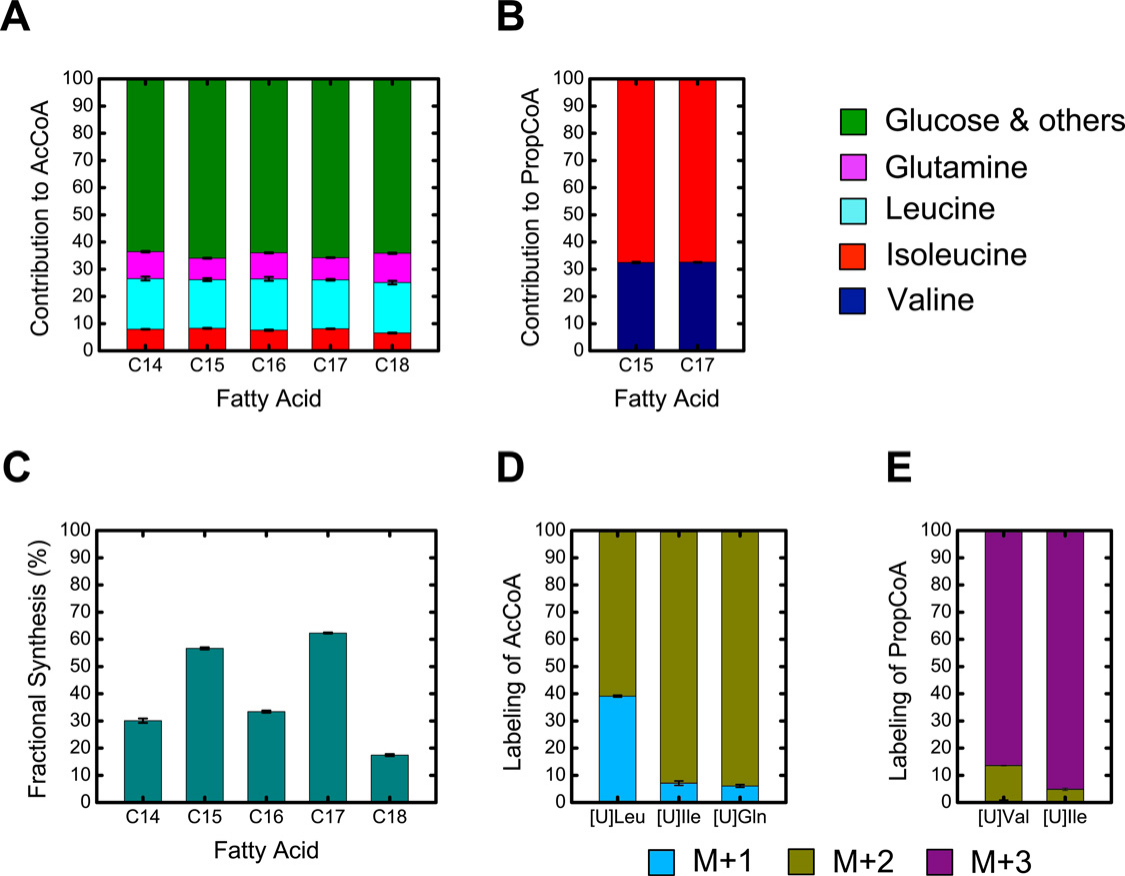
Isotopomer spectral analysis (ISA) results. (A) Estimated D(AcCoA)-values, fractional contributions to lipogenic acetyl-CoA precursor pool. (B) Estimated D(PropCoA)-values, fractional contributions to lipogenic propionyl-CoA precursor pool. (C) Estimated g(24h)-values, fractional new synthesis of each fatty acid during the 24 hr tracer experiment between days 6 and 7. (D & E) Estimated mass isotopomer labeling (M+1, M+2 and M+3) of AcCoA and PropCoA precursor pools from the various ^13^C-tracers used in this study.

The estimated D-values are shown in Fig 9A and 9B. The largest contributor to lipogenic AcCoA was leucine at ~18%, followed by glutamine (~10%) and isoleucine (~7%). The remaining contribution to lipogenic AcCoA is predominantly derived from glucose (data not shown). For the odd chain fatty acids, the largest contributor to PropCoA was isoleucine (~67%) with the remaining 33% accounted for by valine (Fig 9B). Since both even and odd chain fatty acids derive their AcCoA units from the same pool, it was encouraging that the D-values estimated for all fatty acids agreed well. Fatty acid g-values are shown in Fig 9C. The odd chain fatty acids C15:0 and C17:0 saw the greatest fold-increase between days 6 and 7, with ~55% fractional new synthesis, while stearic acid (C18:0) had the smallest increase (~20% fractional new synthesis).

From ISA analysis, we also determined the predicted mass isotopomer labeling of AcCoA and PropCoA pools derived from the various ^13^C-tracers (Fig 9D and 9E). For AcCoA, we determined that [U-^13^C]isoleucine and [U-^13^C]glutamine produced AcCoA that was 94% M+2 labeled and 6% M+1 labeled, while [U-^13^C]leucine produced AcCoA that was 61% M+2 labeled and 39% M+1 labeled. For PropCoA, we determined that [U-^13^C]isoleucine produced PropCoA that was 95% M+3 labeled and 5% M+2 labeled, and [U-^13^C]valine produced PropCoA that was 87% M+3 labeled and 13% M+2 labeled.

**Fig 10:**
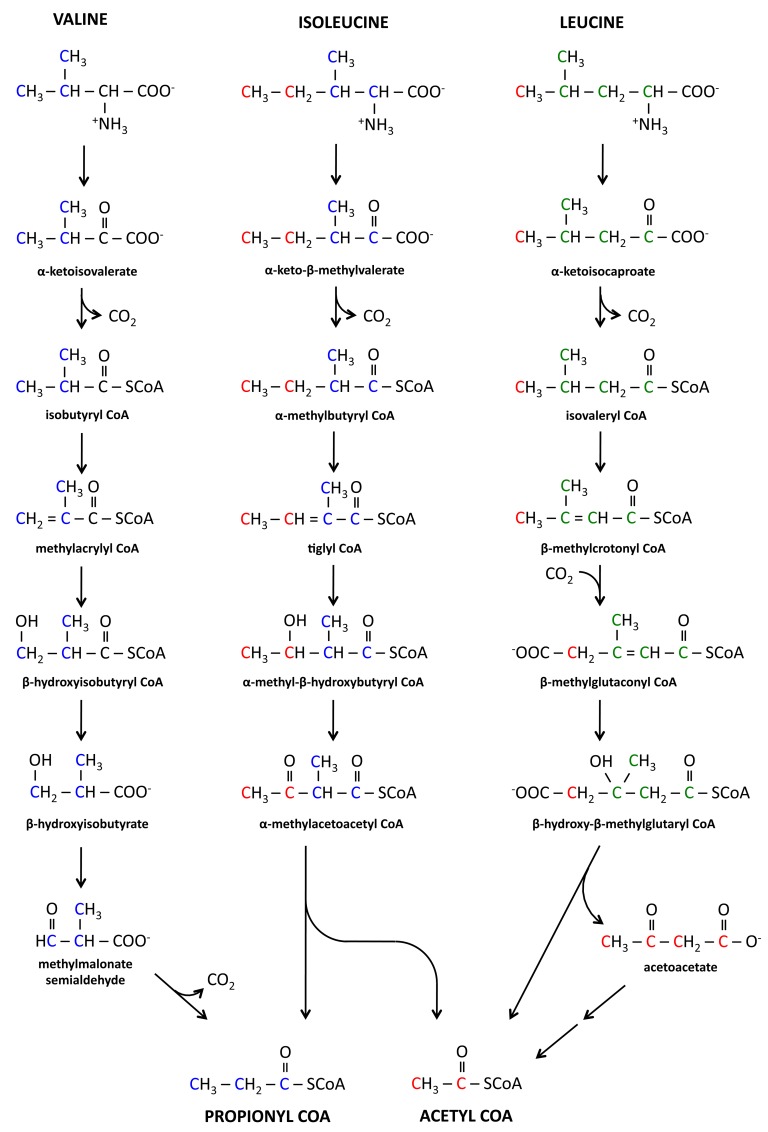
Pathways for branched chain amino acid catabolism. Valine degradation produces one propionyl-CoA, isoleucine catabolism produces one propionyl-CoA and one acetyl-CoA, and leucine catabolism produces three acetyl-CoA. Colors indicate the carbons which arise in propionyl-CoA (blue), acetyl-CoA (red), and acetoacetate (green).

To explain these results, catabolic pathways for BCAA are shown in Fig 10. From leucine degradation three molecules of AcCoA are produced, one directly and two from acetoacetate catabolism. An interesting fact of leucine catabolism is that it is first decarboxylated to form isovaleryl-CoA and later carboxylated at a different position. The decarboxylation and subsequent carboxylation steps will produce incomplete labeling of AcCoA derived from [U-^13^C]leucine. Assuming that incorporated CO_2_ is completely unlabeled, [U-^13^C]leucine will produce AcCoA that is about 67% M+2 and 33% M+1 labeled, which is in good agreement with the ISA modeling results.

## Discussion

In this study, we have investigated the connection between branched chain amino acid and fatty acid metabolism in 3T3-L1 adipocytes. Parallel labeling experiments were performed where the DMEM medium was supplemented with additional amino acids consisting of a mixture of valine, leucine, isoleucine and glutamine. Of the supplemented amino acids, one was uniformally ^13^C-labeled and the remaining three were unlabeled, i.e. natural ^13^C-abundance. Measured fatty acid labeling data were then used to determine the fractional contributions of BCAA and glutamine to AcCoA and PropCoA precursor pools (D-values), as well as the fraction of newly synthesized fatty acids (g-values). First, we demonstrated that 3T3-L1 cells accumulated both even and odd chain length fatty acids. Using a novel GC-MS method, we also demonstrated that PropCoA acts as the primer on fatty acid synthase for the production of odd chain fatty acids.

Previously, the contributions of glucose and acetate to palmitic acid synthesis in 3T3-L1 cells were determined [30]. In addition, contributions to fatty acid synthesis in brown adipocytes have been investigated for several metabolites, including glucose, glutamine, acetate and acetoacetate [31,43]. In this work, we have determined for the first time the contributions of BCAA to fatty acid synthesis. First, we observed high consumption rates of BCAA by 3T3-L1 cells, where the combined uptake rate of BCAA was 63 nmol/10^6^ cells/h, or about 28% that of the glucose uptake rate. We determined that BCAA contributed significantly to lipogenic AcCoA and PropCoA pools. Based on ISA modeling results, we determined that ~25% of lipogenic AcCoA was derived from leucine and isoleucine. This value was consistent for all measured fatty acids, which included C14:0, C15:0, C16:0, C17:0 and C18:0. Despite the fact that there was no net consumption of glutamine, glutamine still contributed ~10% to AcCoA due to high exchange between medium glutamine and intracellular glutamine. Lipogenic PropCoA was derived solely from isoleucine and valine catabolism. From these results, it is evident that all three BCAA contribute a significant portion to overall lipid synthesis. It is important to note that the BCAA concentrations used in these experiments are several fold greater than observed in plasma and ~2-fold greater than DMEM media. While the concentrations are supra-physiological, these experiments do demonstrate the capacity of the 3T3-L1 adipocyte model to catabolize BCAA and the interconnection between fatty acid and BCAA metabolism.

One important observation from the [U-^13^C]glutamine experiment was the transience of ^13^C-labeling in the medium. Initially, glutamine was 100% M+5 labeled; however, at the end of the 24-hour incubation, M+5 labeling was only about 60%. An assumption of ISA modeling is that the precursor pools are at steady state isotopic labeling, i.e. the labeling does not change with time. For the [U-^13^C]glutamine experiment this assumption is thus not valid. Since external glutamine labeling is changing with time, this will have a dynamic effect on the labeling of the resulting AcCoA pool as well. It is important to note, however, that the steady state assumption was valid for the other ^13^C-tracers used in this study. The observation of non-steady labeling of AcCoA for ^13^C-glutamine highlights one of the limitations of the ISA modeling technique. In light of this observation, it is important to discuss how it may affect the modeling results.

Since [U-^13^C]glutamine labeling data is non-steady, the corresponding measured mass isotopomers will be a time-weighted average labeling and the total amount of ^13^C-labeling incorporation will be less than would be expected if [U-^13^C]glutamine labeling remained constant throughout the entire experiment. As a result, the g-parameter would be a time-averaged value. The value will be less than determined from [U-^13^C]leucine and [U-^13^C]isoleucine experiments. The discrepancy in g-values would not be an issue if modeling [U-^13^C]glutamine data alone; however, modeling the data with the other tracer experiments could pose a problem.

To gain more insight, consider the case of odd chain fatty acids. In this case, the [U-^13^C]valine experiments will set a hard constraint on the g-value, since it only contributes to the PropCoA pool and not AcCoA pool. This will cause the g-value to be fixed at the true value, although this value will be greater than would be predicted with [U-^13^C]glutamine. As a result, there will be too much ^13^C-labeling to explain the [U-^13^C]glutamine data and in order to compensate for this the model will decrease the amount of labeling, e.g. by decreasing the D-value for glutamine. The AcCoA and PropCoA precursor pools are interconnected due to isoleucine contributing to both pools. As a result, errors resulting in D-values for the AcCoA may cause indirect effects on the PropCoA pool (i.e. the low PropCoA contribution of M+2 from [U-^13^C]isoleucine). Based on this logic, we believe that the D-value estimates for AcCoA are underestimates. The glutamine dynamics does not invalidate our findings, but instead of determining the exact contributions, we deem the values to be the lower limits for this system.

An interesting observation of this study was the presence of odd-chain fatty acids in cultured adipocytes, which although uncommon can occur in mammalian cells [44]. We demonstrated through the use of two fatty acid derivatization methods that PropCoA serves as a primer on fatty acid synthase. Enzyme-bound PropCoA is then extended by AcCoA polymerization as is the case for even chain fatty acid synthesis. To our knowledge, this is the first time that picolinyl esters have been used for mass isotopomer analysis to elucidate fatty acid pathways.

ISA modeling determined that odd chain fatty acids were primarily synthesized through PropCoA, contrary to published α-oxidation hypotheses [45–47] (Table BU in S1 File). Fatty acid α-oxidation may occur, however, its contribution is minimal compared to PropCoA elongation and can only be detected with sensitive, radioisotope assays [45]. The odd chain fatty acids accumulated at a greater relative rate than their even chain counterparts over the course of the tracer experiments. We believe that odd chain fatty acid accumulation occurs in part due to low activity of vitamin B_12_-dependent methylmalonyl-CoA mutase, which prevents PropCoA from entering into the TCA cycle. From [U-^13^C]valine experiments, we saw negligible labeling incorporation into TCA cycle intermediates (Table L in S1 File), supporting this claim; however when we added [U-^13^C]propionate and vitamin B_12_ to the media as supplements, ^13^C-labeling was seen in TCA cycle metabolites (Table N in S1 File). However, B_12_ supplementation did not completely ameliorate odd chain fatty acid synthesis, indicating that PropCoA derived from valine and isoleucine degradation may enforce mass-action effects on fatty acid synthase. This observation is in alignment with clinical observations of inherited diseases of propionate metabolism. In these disorders, propionate and byproducts of BCAA metabolism cannot assimilate into the TCA cycle and elevated levels of odd-chain fatty acids are commonly observed *in vivo* [48,49].

## Conclusions

This work provides important new insights into the connection between branched chain amino acid (BCAA) catabolism and fatty acid synthesis in adipocytes. We demonstrate that at least 25% of lipogenic acetyl-CoA is derived from BCAA catabolism in cultured 3T3-L1 adipocytes, and that propionyl-CoA, which serves as the precursor for odd chain fatty acid synthesis, is derived from catabolism of valine and isoleucine. Our results suggest that low activity of methylmalonyl-CoA mutase and mass action kinetics of propionyl-CoA on fatty acid synthase contribute to the high rates of odd chain fatty acid synthesis. Another important contribution of this work is in demonstrating the value of using parallel labeling experiments for quantitative pathway elucidation [50,51], and introducing a novel application of picolinyl-esters and GC-MS analysis for quantitative mass isotopomer analysis of fatty acids.

## Supporting Information

S1 File Supporting information. Cell culture data, mass spectrometry data and modeling results.
(DOCX)
