# Supplementary material for: Catabolism of Branched Chain Amino Acids Contributes Significantly to Synthesis of Odd-Chain and Even-Chain Fatty Acids in 3T3-L1 Adipocytes
Source: PLoS One. 2015 Dec 28;10(12):e0145850. doi: 10.1371/journal.pone.0145850 (PMC4692509; doi:10.1371/journal.pone.0145850)
Supplement: S1 File — Cell culture data, mass spectrometry data and modeling results. (DOCX) [file pone.0145850.s001.docx]

Catabolism of Branched Chain Amino Acids Contributes Significantly to Synthesis of Odd-chain and Even-chain Fatty Acids in 3T3-L1 Adipocytes

Scott B. Crown, Nicholas Marze, Maciek R. Antoniewicz

This document contains the following information in support of the primary article:

**Table A.** 3T3-L1 cell density and viability at the time of induction (day 0)

**Table B.** Metabolite fragments measured by GC-MS for analysis of extracellular metabolites

**Table C.** Metabolite fragments measured by GC-MS for analysis of intracellular metabolites

**Table D.** Extracellular metabolite concentrations between day 6 and day 7

**Table E.** Mass isotopomer distributions of tracers in the medium for parallel labeling experiments

**Figure F.** Time course of [U-^13^C]glutamine labeling in the medium between day 6 and day 7

**Table G.** Mass isotopomer distributions of intracellular valine for parallel labeling experiments

**Table H.** Mass isotopomer distributions of intracellular leucine for parallel labeling experiments

**Table I.** Mass isotopomer distributions of intracellular isoleucine for parallel labeling experiments

**Table J.** Mass isotopomer distributions of intracellular glutamine for parallel labeling experiments

**Table K.** Mass isotopomer distributions of intracellular methylmalonate for parallel labeling experiments

**Table L.** Mass isotopomer distributions of intracellular metabolites for labeling experiments with [U-^13^C]valine, and no tracer as control

**Table M.** Mass isotopomer distributions of pentadecanoic acid (C15:0) and palmitic acid (C16:0) (picolinyl ester derivatization) for labeling experiments with [U-^13^C]valine, and no tracer as control

**Table N.** Mass isotopomer distributions of intracellular metabolites for experiments with [U-^13^C]propionate in presence/absence of vitamin B_12_, and no tracer as control

**Table O.** Mass isotopomer distributions of methyl-myristate (C14:0) fragment at *m/z* 242 (C1-C14, FAME derivatization) for parallel labeling experiments

**Table P.** Mass isotopomer distributions of methyl-myristate (C14:0) fragment at *m/z* 242 (C1-C14, FAME derivatization) for 4 experiments with no tracer

**Table Q.** Mass isotopomer distributions of methyl-myristate (C14:0) fragment at *m/z* 242 (C1-C14, FAME derivatization) for 4 parallel labeling experiments with [U-^13^C]valine

**Table R.** Mass isotopomer distributions of methyl-myristate (C14:0) fragment at *m/z* 242 (C1-C14, FAME derivatization) for 4 parallel labeling experiments with [U-^13^C]leucine

**Table S.** Mass isotopomer distributions of methyl-myristate (C14:0) fragment at *m/z* 242 (C1-C14, FAME derivatization) for 4 parallel labeling experiments with [U-^13^C]isoleucine

**Table T.** Mass isotopomer distributions of methyl-myristate (C14:0) fragment at *m/z* 242 (C1-C14, FAME derivatization) for 4 parallel labeling experiments with [U-^13^C]glutamine

**Table U.** Mass isotopomer distributions of methyl-pentadecanoate (C15:0) fragment at *m/z* 256 (C1-C15, FAME derivatization) for parallel labeling experiments

**Table V.** Mass isotopomer distributions of methyl-pentadecanoate (C15:0) fragment at *m/z* 256 (C1-C15, FAME derivatization) for 4 experiments with no tracer

**Table W.** Mass isotopomer distributions of methyl-pentadecanoate (C15:0) fragment at *m/z* 256 (C1-C15, FAME derivatization) for 4 parallel labeling experiments with [U-^13^C]valine

**Table X.** Mass isotopomer distributions of methyl-pentadecanoate (C15:0) fragment at *m/z* 256 (C1-C15, FAME derivatization) for 4 parallel labeling experiments with [U-^13^C]leucine

**Table Y.** Mass isotopomer distributions of methyl-pentadecanoate (C15:0) fragment at *m/z* 256 (C1-C15, FAME derivatization) for 4 parallel labeling experiments with [U-^13^C]isoleucine

**Table Z.** Mass isotopomer distributions of methyl-pentadecanoate (C15:0) fragment at *m/z* 256 (C1-C15, FAME derivatization) for 4 parallel labeling experiments with [U-^13^C]glutamine

**Table AA.** Mass isotopomer distributions of methyl-palmitate (C16:0) fragment at *m/z* 270 (C1-C16, FAME derivatization) for parallel labeling experiments

**Table AB.** Mass isotopomer distributions of methyl-palmitate (C16:0) fragment at *m/z* 270 (C1-C16, FAME derivatization) for 4 experiments with no tracer

**Table AC.** Mass isotopomer distributions of methyl-palmitate (C16:0) fragment at *m/z* 270 (C1-C16, FAME derivatization) for 4 parallel labeling experiments with [U-^13^C]valine

**Table AD.** Mass isotopomer distributions of methyl-palmitate (C16:0) fragment at *m/z* 270 (C1-C16, FAME derivatization) for 4 parallel labeling experiments with [U-^13^C]leucine

**Table AE.** Mass isotopomer distributions of methyl-palmitate (C16:0) fragment at *m/z* 270 (C1-C16, FAME derivatization) for 4 parallel labeling experiments with [U-^13^C]isoleucine

**Table AF.** Mass isotopomer distributions of methyl-palmitate (C16:0) fragment at *m/z* 270 (C1-C16, FAME derivatization) for 4 parallel labeling experiments with [U-^13^C]glutamine

**Table AG.** Mass isotopomer distributions of methyl-heptadecanoate (C17:0) fragment at *m/z* 284 (C1-C17, FAME derivatization) for parallel labeling experiments

**Table AH.** Mass isotopomer distributions of methyl-heptadecanoate (C17:0) fragment at *m/z* 284 (C1-C17, FAME derivatization) for 4 experiments with no tracer

**Table AI.** Mass isotopomer distributions of methyl-heptadecanoate (C17:0) fragment at *m/z* 284 (C1-C17, FAME derivatization) for 4 parallel labeling experiments with [U-^13^C]valine

**Table AJ.** Mass isotopomer distributions of methyl-heptadecanoate (C17:0) fragment at *m/z* 284 (C1-C17, FAME derivatization) for 4 parallel labeling experiments with [U-^13^C]leucine

**Table AK.** Mass isotopomer distributions of methyl-heptadecanoate (C17:0) fragment at *m/z* 284 (C1-C17, FAME derivatization) for 4 parallel labeling experiments with [U-^13^C]isoleucine

**Table AL.** Mass isotopomer distributions of methyl-heptadecanoate (C17:0) fragment at *m/z* 284 (C1-C17, FAME derivatization) for 4 parallel labeling experiments with [U-^13^C]glutamine

**Table AM.** Mass isotopomer distributions of methyl-stearate (C18:0) fragment at *m/z* 298 (C1-C18, FAME derivatization) for parallel labeling experiments

**Table AN.** Mass isotopomer distributions of methyl-stearate (C18:0) fragment at *m/z* 298 (C1-C18, FAME derivatization) for 4 experiments with no tracer

**Table AO.** Mass isotopomer distributions of methyl-stearate (C18:0) fragment at *m/z* 298 (C1-C18, FAME derivatization) for 4 parallel labeling experiments with [U-^13^C]valine

**Table AP.** Mass isotopomer distributions of methyl-stearate (C18:0) fragment at *m/z* 298 (C1-C18, FAME derivatization) for 4 parallel labeling experiments with [U-^13^C]leucine

**Table AQ.** Mass isotopomer distributions of methyl-stearate (C18:0) fragment at *m/z* 298 (C1-C18, FAME derivatization) for 4 parallel labeling experiments with [U-^13^C]isoleucine

**Table AR.** Mass isotopomer distributions of methyl-stearate (C18:0) fragment at *m/z* 298 (C1-C18, FAME derivatization) for 4 parallel labeling experiments with [U-^13^C]glutamine

**Table AS.** ISA models for even chain fatty acids.

**Table AT.** ISA models for odd chain fatty acids.

**Table AU.** ISA model fits for myristate (C14:0).

**Table AV.** Measured vs. simulated mass isotopomer distributions of methyl-myristate (C14:0) for parallel labeling experiment set #1

**Table AW.** Measured vs. simulated mass isotopomer distributions of methyl-myristate (C14:0) for parallel labeling experiment set #2

**Table AX.** Measured vs. simulated mass isotopomer distributions of methyl-myristate (C14:0) for parallel labeling experiment set #3

**Table AY.** Measured vs. simulated mass isotopomer distributions of methyl-myristate (C14:0) for parallel labeling experiment set #4

**Table AZ.** ISA model fits for pentadecanoate (C15:0).

**Table BA.** Measured vs. simulated mass isotopomer distributions of methyl-pentadecanoate (C15:0) for parallel labeling experiment set #1

**Table BB.** Measured vs. simulated mass isotopomer distributions of methyl-pentadecanoate (C15:0) for parallel labeling experiment set #2

**Table BC.** Measured vs. simulated mass isotopomer distributions of methyl-pentadecanoate (C15:0) for parallel labeling experiment set #3

**Table BD.** Measured vs. simulated mass isotopomer distributions of methyl-pentadecanoate (C15:0) for parallel labeling experiment set #4

**Table BE.** ISA model fits for palmitate (C16:0).

**Table BF.** Measured vs. simulated mass isotopomer distributions of methyl-palmitate (C16:0) for parallel labeling experiment set #1

**Table BG.** Measured vs. simulated mass isotopomer distributions of methyl-palmitate (C16:0) for parallel labeling experiment set #2

**Table BH.** Measured vs. simulated mass isotopomer distributions of methyl-palmitate (C16:0) for parallel labeling experiment set #3

**Table BI.** Measured vs. simulated mass isotopomer distributions of methyl-palmitate (C16:0) for parallel labeling experiment set #4

**Table BJ.** ISA model fits for heptadecanoate (C17:0).

**Table BK.** Measured vs. simulated mass isotopomer distributions of methyl-heptadecanoate (C17:0) for parallel labeling experiment set #1

**Table BL.** Measured vs. simulated mass isotopomer distributions of methyl-heptadecanoate (C17:0) for parallel labeling experiment set #2

**Table BM.** Measured vs. simulated mass isotopomer distributions of methyl-heptadecanoate (C17:0) for parallel labeling experiment set #3

**Table BN.** Measured vs. simulated mass isotopomer distributions of methyl-heptadecanoate (C17:0) for parallel labeling experiment set #4

**Table BO.** ISA model fits for stearate (C18:0).

**Table BP.** Measured vs. simulated mass isotopomer distributions of methyl-stearate (C18:0) for parallel labeling experiment set #1

**Table BQ.** Measured vs. simulated mass isotopomer distributions of methyl-stearate (C18:0) for parallel labeling experiment set #2

**Table BR.** Measured vs. simulated mass isotopomer distributions of methyl-stearate (C18:0) for parallel labeling experiment set #3

**Table BS.** Measured vs. simulated mass isotopomer distributions of methyl-stearate (C18:0) for parallel labeling experiment set #4

**Table BT.** Combined ISA model for all fatty acids.

**Table BU.** Combined ISA model results.

**Table BV.** Measured vs. simulated mass isotopomer distributions of all fatty acids for parallel labeling experiment set #1, combined ISA model

**Table BW.** Measured vs. simulated mass isotopomer distributions of all fatty acids for parallel labeling experiment set #2, combined ISA model

**Table BX.** Measured vs. simulated mass isotopomer distributions of all fatty acids for parallel labeling experiment set #3, combined ISA model

**Table BY.** Measured vs. simulated mass isotopomer distributions of all fatty acids for parallel labeling experiment set #4, combined ISA model

**Table A.** 3T3-L1 cell density and viability at the time of induction (day 0).

|  | Viable cell density (10^6^ cells/mL) | Viability  (%) |
| --- | --- | --- |
| Flask 1 | 1.34 | 94.6 |
| Flask 2 | 1.25 | 91.4 |
| Flask 3 | 1.31 | 94.1 |
| Flask 4 | 1.35 | 96.3 |
| Flask 5 | 1.24 | 97.3 |
| Flask 6 | 1.19 | 96.1 |
| Mean ± SEM | 1.28 ± 0.03 | 94.9 ± 0.9 |

**Table B.** Metabolite fragments measured by GC-MS for analysis of extracellular metabolites

| Metabolite | Mass | Carbon atoms | Fragment formula | Internal standard | Concentration  of standard (mM) |
| --- | --- | --- | --- | --- | --- |
| Alanine | 260 | 1-2-3 | C_11_H_26_O_2_NSi_2_ | [U-^13^C]Ala in algal soln. | 7.88 ± 0.07 |
| Glycine | 246 | 1-2 | C_10_H_24_O_2_NSi_2_ | [U-^13^C]Gly in algal soln. | 6.36 ± 0.05 |
| Valine | 288 | 1-2-3-4-5 | C_13_H_30_O_2_NSi_2_ | [U-^13^C]Val in algal soln. | 4.18 ± 0.05 |
| Leucine | 274 | 2-3-4-5-6 | C_13_H_32_ONSi_2_ | [U-^13^C]Leu in algal soln. | 6.39 ± 0.23 |
| Isoleucine | 274 | 2-3-4-5-6 | C_13_H_32_ONSi_2_ | [U-^13^C]Ile in algal soln. | 2.94 ± 0.05 |
| Methionine | 320 | 1-2-3-4-5 | C_13_H_30_O_2_NSi_2_S | [U-^13^C]Met in algal soln. | 0.81 ± 0.08 |
| Serine | 390 | 1-2-3 | C_17_H_40_O_3_NSi_3_ | [U-^13^C]Ser in algal soln. | 2.82 ± 0.08 |
| Threonine | 404 | 1-2-3-4 | C_18_H_42_O_3_NSi_3_ | [U-^13^C]Thr in algal soln. | 3.25 ± 0.06 |
| Phenylalanine | 302 | 1-2 | C_14_H_32_O_2_NSi_2_ | [U-^13^C]Phe in algal soln. | 1.85 ± 0.03 |
| Aspartate | 418 | 1-2-3-4 | C_18_H_40_O_4_NSi_3_ | [U-^13^C]Asp in algal soln. | 5.26 ± 0.12 |
| Glutamate | 432 | 1-2-3-4-5 | C_19_H_42_O_4_NSi_3_ | [U-^13^C]Glu in algal soln. | 6.91 ± 0.11 |
| Tyrosine | 302 | 1-2 | C_14_H_32_O_2_NSi_2_ | [U-^13^C]Tyr in algal soln. | 1.70 ± 0.04 |
| Glutamine | 431 | 1-2-3-4-5 | C_19_H_43_O_3_N_2_Si_3_ | [U-^13^C]Gln | 58.8 ± 0.53 |

**Table C.** Metabolite fragments measured by GC-MS for analysis of intracellular metabolites

| Intracellular metabolite | Mass | Carbon atoms | Fragment formula |
| --- | --- | --- | --- |
| *TBDMS fragments* |  |  |  |
| Valine | 288 | 1-2-3-4-5 | C_13_H_30_O_2_NSi_2_ |
| Methylmalonate | 289 | 1-2-3-4 | C_12_H_25_O_4_Si_2_ |
| Leucine | 274 | 2-3-4-5-6 | C_13_H_32_ONSi_2_ |
| Isoleucine | 274 | 2-3-4-5-6 | C_13_H_32_ONSi_2_ |
| Succinate | 289 | 1-2-3-4 | C_12_H_25_O_4_Si_2_ |
| α-ketoglutarate | 346 | 1-2-3-4-5 | C_14_H_28_O_5_NSi_2_ |
| Malate | 419 | 1-2-3-4 | C_18_H_39_O_5_Si_3_ |
| Glutamine | 431 | 1-2-3-4-5 | C_19_H_43_O_3_N_2_Si_3_ |
| Citrate | 459 | 1-2-3-4-5-6 | C_20_H_39_O_6_Si_3_ |
|  |  |  |  |
| *Methyl ester fragments* |  |  |  |
| Myristic acid (C14:0) | 242 | 1- … -14 | C_15_H_30_O_2_ |
| Pentadecanoic acid (C15:0) | 256 | 1- … -15 | C_16_H_32_O_2_ |
| Palmitic acid (C16:0) | 270 | 1- … -16 | C_17_H_34_O_2_ |
| Heptadecanoic acid (C17:0) | 284 | 1- … -17 | C_18_H_36_O_2_ |
| Stearic acid (C18:0) | 298 | 1- … -18 | C_19_H_38_O_2_ |
|  |  |  |  |
| *Picolinyl ester fragments* |  |  |  |
| Pentadecanoic acid (ω-1) | 318 | 1- … -14 | C_20_H_32_O_2_N |
| Pentadecanoic acid (ω-2) | 304 | 1- … -13 | C_19_H_30_O_2_N |
| Pentadecanoic acid (ω-3) | 290 | 1- … -12 | C_18_H_28_O_2_N |
| Palmitic acid (ω-1) | 332 | 1- … -15 | C_21_H_34_O_2_N |
| Palmitic acid (ω-2) | 318 | 1- … -14 | C_20_H_32_O_2_N |
| Palmitic acid (ω-3) | 304 | 1- … -13 | C_19_H_30_O_2_N |

**Table D.** Extracellular metabolite concentrations between day 6 (t=0h) and day 7 (t=24h) (mean ± stdev, *n*=4 biological replicates).

|  | Extracellular concentration (mM) | | | |
| --- | --- | --- | --- | --- |
| Metabolite | 0 h | 6 h | 12 h | 24 h |
| Glucose | 19.7 ± 0.1 | 18.2 ± 0.3 | 16.4 ± 0.6 | 12.8 ± 0.8 |
| Lactate | 1.7 ± 0.1 | 3.9 ± 0.5 | 5.2 ± 0.8 | 6.6 ± 1.1 |
| Alanine | 0.20 ± 0.01 | 0.46 ± 0.03 | 0.54 ± 0.03 | 0.56 ± 0.03 |
| Glycine | 0.51 ± 0.02 | 0.61 ± 0.01 | 0.70 ± 0.02 | 0.88 ± 0.04 |
| Valine | 1.66 ± 0.00 | 1.53 ± 0.05 | 1.41 ± 0.04 | 1.22 ± 0.02 |
| Leucine | 1.69 ± 0.01 | 1.41 ± 0.05 | 1.20 ± 0.05 | 0.88 ± 0.03 |
| Isoleucine | 1.58 ± 0.03 | 1.32 ± 0.02 | 1.14 ± 0.04 | 0.84 ± 0.02 |
| Methionine | 0.21 ± 0.00 | 0.21 ± 0.01 | 0.20 ± 0.00 | 0.19 ± 0.00 |
| Serine | 0.40 ± 0.01 | 0.37 ± 0.01 | 0.35 ± 0.01 | 0.33 ± 0.02 |
| Threonine | 0.70 ± 0.04 | 0.69 ± 0.01 | 0.69 ± 0.01 | 0.74 ± 0.03 |
| Phenylalanine | 0.38 ± 0.01 | 0.39 ± 0.00 | 0.40 ± 0.01 | 0.41 ± 0.01 |
| Aspartate | 0.04 ± 0.00 | 0.01 ± 0.00 | 0.00 ± 0.00 | 0.00 ± 0.00 |
| Glutamate | 0.09 ± 0.01 | 0.03 ± 0.01 | 0.01 ± 0.00 | 0.01 ± 0.01 |
| Tyrosine | 0.35 ± 0.01 | 0.36 ± 0.01 | 0.36 ± 0.01 | 0.37 ± 0.01 |
| Glutamine | Not measured | 3.59 ± 0.07 | 3.75 ± 0.08 | 4.12 ± 0.10 |

**Table E.** Mass isotopomer distributions (MIDs) of isotopic tracers in the medium (TBDMS derivatized amino acids) for parallel labeling experiments with [U-^13^C]valine, [U-^13^C]leucine, [U-^13^C,^15^N]isoleucine, and [U-^13^C]glutamine measured on day 7 (t=24h). Shown are the raw MIDs and MIDs corrected for natural isotope abundances.

| Mass isotopomer | [U-^13^C]Val  *m/z* 288  (C1-C5) | | [U-^13^C]Leu  *m/z* 274  (C1-C6) | | [U-^13^C,^15^N]Ile  *m/z* 274  (C1-C6) | | [U-^13^C]Gln*  *m/z* 431  (C1-C5) | |
| --- | --- | --- | --- | --- | --- | --- | --- | --- |
|  | Raw | Corrected | Raw | Corrected | Raw | Corrected | Raw | Corrected |
| M0 | 0.402 | 0.549 | 0.424 | 0.579 | 0.333 | 0.455 | 0.260 | 0.415 |
| M1 | 0.104 | 0.004 | 0.106 | 0.000 | 0.135 | 0.069 | 0.100 | 0.003 |
| M2 | 0.041 | 0.001 | 0.042 | 0.001 | 0.047 | 0.002 | 0.061 | 0.024 |
| M3 | 0.008 | 0.001 | 0.008 | 0.002 | 0.012 | 0.002 | 0.025 | 0.013 |
| M4 | 0.021 | 0.026 | 0.022 | 0.027 | 0.015 | 0.018 | 0.027 | 0.029 |
| M5 | 0.329 | 0.421 | 0.311 | 0.396 | 0.270 | 0.345 | 0.351 | 0.518 |
| M6 | 0.065 |  | 0.061 |  | 0.139 | 0.109 | 0.111 |  |
| M7 | 0.029 |  | 0.026 |  | 0.039 |  | 0.053 |  |
| M8 |  |  |  |  | 0.010 |  | 0.012 |  |

* [U-^13^C]glutamine labeling in the medium on day 6 (t=0h) was 97% [U-^13^C]glutamine

**Figure F.** Time course of [U-^13^C]glutamine labeling in the medium between day 6 (t=0h) and day 7 (t=24h).


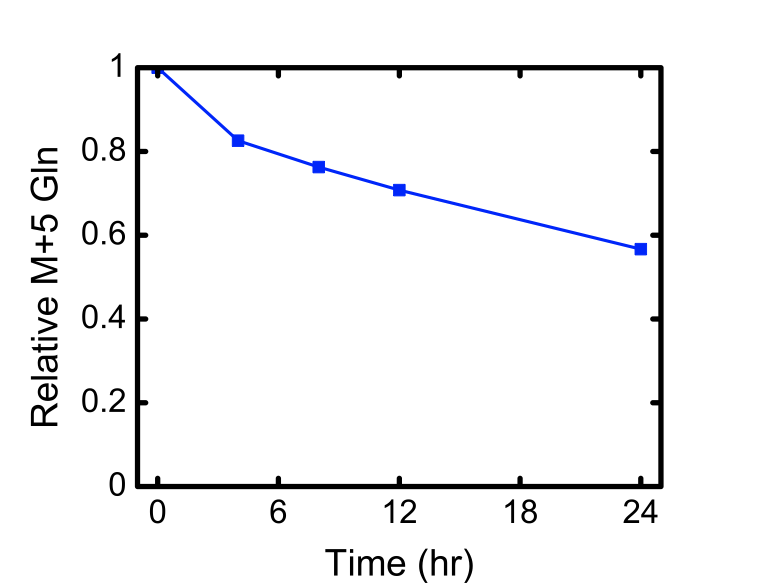


**Table G.** Mass isotopomer distributions (MIDs) of intracellular valine fragment at *m/z* 288 (TBDMS, C1-C5) for parallel labeling experiments with [U-^13^C]valine, [U-^13^C]leucine, [U-^13^C,^15^N]isoleucine, and [U-^13^C]glutamine measured on day 7 (t=24h). Shown are the raw MIDs and MIDs corrected for natural isotope abundances

| *m/z* | [U-^13^C]Val | | [U-^13^C]Leu | | [U-^13^C,^15^N]Ile | | [U-^13^C]Gln | |
| --- | --- | --- | --- | --- | --- | --- | --- | --- |
|  | Raw | Corrected | Raw | Corrected | Raw | Corrected | Raw | Corrected |
| 288 (M0) | 0.379 | 0.517 | 0.730 | 1.000 | 0.670 | 0.919 | 0.730 | 0.999 |
| 289 (M1) | 0.096 | 0.000 | 0.183 | 0.000 | 0.229 | 0.081 | 0.183 | 0.000 |
| 290 (M2) | 0.038 | 0.000 | 0.072 | 0.000 | 0.081 | 0.000 | 0.072 | 0.000 |
| 291 (M3) | 0.007 | 0.001 | 0.012 | 0.000 | 0.017 | 0.000 | 0.012 | 0.000 |
| 292 (M4) | 0.018 | 0.022 | 0.002 | 0.000 | 0.003 | 0.000 | 0.002 | 0.000 |
| 293 (M5) | 0.360 | 0.460 | 0.000 | 0.000 | 0.001 | 0.000 | 0.001 | 0.001 |
| 294 (M6) | 0.071 |  | 0.000 |  | 0.000 |  | 0.000 |  |
| 295 (M7) | 0.031 |  | 0.000 |  | 0.000 |  | 0.000 |  |

**Table H.** Mass isotopomer distributions (MIDs) of intracellular leucine fragment at *m/z* 274 (TBDMS, C1-C5) for parallel labeling experiments with [U-^13^C]valine, [U-^13^C]leucine, [U-^13^C,^15^N]isoleucine, and [U-^13^C]glutamine measured on day 7 (t=24h). Shown are the raw MIDs and MIDs corrected for natural isotope abundances

| *m/z* | [U-^13^C]Val | | [U-^13^C]Leu | | [U-^13^C,^15^N]Ile | | [U-^13^C]Gln | |
| --- | --- | --- | --- | --- | --- | --- | --- | --- |
|  | Raw | Corrected | Raw | Corrected | Raw | Corrected | Raw | Corrected |
| 274 (M0) | 0.729 | 0.999 | 0.424 | 0.578 | 0.638 | 0.873 | 0.731 | 1.000 |
| 275 (M1) | 0.183 | 0.000 | 0.107 | 0.000 | 0.249 | 0.119 | 0.184 | 0.000 |
| 276 (M2) | 0.070 | 0.000 | 0.041 | 0.000 | 0.083 | 0.000 | 0.070 | 0.000 |
| 277 (M3) | 0.012 | 0.000 | 0.008 | 0.001 | 0.018 | 0.000 | 0.012 | 0.000 |
| 278 (M4) | 0.002 | 0.000 | 0.018 | 0.021 | 0.003 | 0.000 | 0.002 | 0.000 |
| 279 (M5) | 0.002 | 0.001 | 0.313 | 0.400 | 0.006 | 0.007 | 0.001 | 0.000 |
| 280 (M6) | 0.000 |  | 0.062 |  | 0.002 |  | 0.000 |  |
| 281 (M7) | 0.000 |  | 0.026 |  | 0.001 |  | 0.000 |  |

**Table I.** Mass isotopomer distributions (MIDs) of intracellular isoleucine fragment at *m/z* 274 (TBDMS, C1-C5) for parallel labeling experiments with [U-^13^C]valine, [U-^13^C]leucine, [U-^13^C,^15^N]isoleucine, and [U-^13^C]glutamine measured on day 7 (t=24h). Shown are the raw MIDs and MIDs corrected for natural isotope abundances

| *m/z* | [U-^13^C]Val | | [U-^13^C]Leu | | [U-^13^C,^15^N]Ile | | [U-^13^C]Gln | |
| --- | --- | --- | --- | --- | --- | --- | --- | --- |
|  | Raw | Corrected | Raw | Corrected | Raw | Corrected | Raw | Corrected |
| 274 (M0) | 0.727 | 0.996 | 0.726 | 0.994 | 0.334 | 0.452 | 0.728 | 0.997 |
| 275 (M1) | 0.186 | 0.003 | 0.186 | 0.003 | 0.129 | 0.060 | 0.186 | 0.002 |
| 276 (M2) | 0.071 | 0.000 | 0.072 | 0.000 | 0.045 | 0.002 | 0.071 | 0.000 |
| 277 (M3) | 0.012 | 0.000 | 0.012 | 0.000 | 0.011 | 0.001 | 0.012 | 0.000 |
| 278 (M4) | 0.002 | 0.000 | 0.002 | 0.000 | 0.013 | 0.015 | 0.002 | 0.000 |
| 279 (M5) | 0.001 | 0.001 | 0.002 | 0.002 | 0.295 | 0.376 | 0.001 | 0.000 |
| 280 (M6) | 0.000 |  | 0.001 |  | 0.134 | 0.095 | 0.000 |  |
| 281 (M7) | 0.000 |  | 0.000 |  | 0.039 |  | 0.000 |  |

**Table J.** Mass isotopomer distributions (MIDs) of intracellular glutamine fragment at *m/z* 431 (TBDMS, C1-C5) for parallel labeling experiments with [U-^13^C]valine, [U-^13^C]leucine, [U-^13^C,^15^N]isoleucine, and [U-^13^C]glutamine measured on day 7 (t=24h). Shown are the raw MIDs and MIDs corrected for natural isotope abundances

| *m/z* | [U-^13^C]Val | | [U-^13^C]Leu | | [U-^13^C,^15^N]Ile | | [U-^13^C]Gln | |
| --- | --- | --- | --- | --- | --- | --- | --- | --- |
|  | Raw | Corrected | Raw | Corrected | Raw | Corrected | Raw | Corrected |
| 431 (M0) | 0.627 | 0.996 | 0.618 | 0.989 | 0.578 | 0.925 | 0.277 | 0.436 |
| 432 (M1) | 0.231 | 0.000 | 0.231 | 0.000 | 0.254 | 0.059 | 0.107 | 0.005 |
| 433 (M2) | 0.105 | 0.000 | 0.112 | 0.009 | 0.121 | 0.013 | 0.065 | 0.025 |
| 434 (M3) | 0.028 | 0.004 | 0.029 | 0.002 | 0.035 | 0.002 | 0.026 | 0.012 |
| 435 (M4) | 0.007 | 0.000 | 0.007 | 0.000 | 0.009 | 0.000 | 0.024 | 0.023 |
| 436 (M5) | 0.002 | 0.000 | 0.002 | 0.000 | 0.002 | 0.000 | 0.343 | 0.500 |
| 437 (M6) | 0.000 |  | 0.000 |  | 0.000 |  | 0.108 |  |
| 438 (M7) | 0.000 |  | 0.000 |  | 0.000 |  | 0.051 |  |

**Table K.** Mass isotopomer distributions (MIDs) of intracellular methylmalonate fragment at *m/z* 289 (TBDMS, C1-C4) for parallel labeling experiments with [U-^13^C]valine, [U-^13^C]leucine, [U-^13^C,^15^N]isoleucine, and [U-^13^C]glutamine measured on day 7 (t=24h). Shown are the raw MIDs and MIDs corrected for natural isotope abundances

| *m/z* | [U-^13^C]Val | | [U-^13^C]Leu | | [U-^13^C,^15^N]Ile | | [U-^13^C]Gln | |
| --- | --- | --- | --- | --- | --- | --- | --- | --- |
|  | Raw | Corrected | Raw | Corrected | Raw | Corrected | Raw | Corrected |
| 289 (M0) | 0.595 | 0.807 | 0.739 | 1.000 | 0.477 | 0.647 | 0.737 | 1.000 |
| 290 (M1) | 0.158 | 0.022 | 0.174 | 0.000 | 0.140 | 0.035 | 0.175 | 0.000 |
| 291 (M2) | 0.087 | 0.032 | 0.073 | 0.000 | 0.099 | 0.059 | 0.073 | 0.000 |
| 292 (M3) | 0.119 | 0.135 | 0.012 | 0.000 | 0.209 | 0.248 | 0.012 | 0.000 |
| 293 (M4) | 0.028 | 0.004 | 0.002 | 0.000 | 0.053 | 0.011 | 0.002 | 0.000 |
| 294 (M5) | 0.011 |  | 0.000 |  | 0.020 |  | 0.000 |  |
| 295 (M6) | 0.002 |  | 0.000 |  | 0.003 |  | 0.000 |  |

**Table L.** Mass isotopomer distributions of intracellular metabolites for labeling experiments with **[U-^13^C]valine** and **no tracer**, measured on day 7, i.e. after 24 h incubation with the tracer (mean, *n*=2 biological replicates). Data was not corrected for natural isotope abundances.

| Intracellular metab. *m/z* of fragment  (C-atoms) | Tracer | M0 | M1 | M2 | M3 | M4 | M5 | M6 |
| --- | --- | --- | --- | --- | --- | --- | --- | --- |
| Methylmalonate  *m/z* 289 | [U-^13^C]Val | 0.595 | 0.158 | 0.087 | 0.119 | 0.028 | 0.011 | 0.002 |
| (C1-C4) | No tracer | 0.739 | 0.174 | 0.073 | 0.012 | 0.002 | 0.000 | 0.000 |
|  |  |  |  |  |  |  |  |  |
| Succinate  *m/z* 289 | [U-^13^C]Val | 0.722 | 0.180 | 0.074 | 0.016 | 0.004 | 0.002 | 0.000 |
| (C1-C4) | No tracer | 0.728 | 0.178 | 0.075 | 0.014 | 0.004 | 0.002 | 0.000 |
|  |  |  |  |  |  |  |  |  |
| Malate  *m/z* 419 | [U-^13^C]Val | 0.638 | 0.224 | 0.105 | 0.025 | 0.006 | 0.001 | 0.000 |
| (C1-C4) | No tracer | 0.639 | 0.224 | 0.105 | 0.025 | 0.006 | 0.001 | 0.000 |
|  |  |  |  |  |  |  |  |  |
| Citrate  *m/z* 459 | [U-^13^C]Val | 0.612 | 0.229 | 0.111 | 0.028 | 0.009 | 0.002 | 0.004 |
| (C1-C6) | No tracer | 0.613 | 0.229 | 0.110 | 0.028 | 0.009 | 0.002 | 0.004 |
|  |  |  |  |  |  |  |  |  |
| α-ketoglutarate  *m/z* 346 | [U-^13^C]Val | 0.711 | 0.192 | 0.078 | 0.014 | 0.003 | 0.001 | 0.001 |
| (C1-C5) | No tracer | 0.713 | 0.191 | 0.077 | 0.014 | 0.003 | 0.001 | 0.001 |

**Table M.** Mass isotopomer distributions of pentadecanoic acid (C15:0) and palmitic acid (C16:0) fragments (picolinyl ester derivatization) for labeling experiments with **[U-^13^C]valine** and **no tracer**, measured on day 7, i.e. after 24 h incubation with the tracer (mean, *n*=2 biological replicates). Data was not corrected for natural isotope abundances.

| Fatty acid *m/z* of fragment  (C-atoms) | Tracer | M0 | M1 | M2 | M3 | M4 | M5 |
| --- | --- | --- | --- | --- | --- | --- | --- |
| C15:0  *m/z* 318 | [U-^13^C]Val | 0.722 | 0.174 | 0.080 | 0.017 | 0.003 | 0.001 |
| (C1-C14) | No tracer | 0.790 | 0.180 | 0.024 | 0.002 | 0.001 | 0.001 |
|  |  |  |  |  |  |  |  |
| C15:0  *m/z* 304 | [U-^13^C]Val | 0.736 | 0.220 | 0.033 | 0.006 | 0.001 | 0.000 |
| (C1-C13) | No tracer | 0.797 | 0.174 | 0.024 | 0.003 | 0.001 | 0.000 |
|  |  |  |  |  |  |  |  |
| C15:0  *m/z* 290 | [U-^13^C]Val | 0.797 | 0.172 | 0.023 | 0.005 | 0.001 | 0.000 |
| (C1-C12) | No tracer | 0.797 | 0.172 | 0.024 | 0.003 | 0.001 | 0.001 |
|  |  |  |  |  |  |  |  |
| C16:0  *m/z* 332 | [U-^13^C]Val | 0.784 | 0.183 | 0.025 | 0.005 | 0.001 | 0.001 |
| (C1-C15) | No tracer | 0.787 | 0.183 | 0.025 | 0.003 | 0.001 | 0.001 |
|  |  |  |  |  |  |  |  |
| C16:0  *m/z* 318 | [U-^13^C]Val | 0.793 | 0.180 | 0.023 | 0.001 | 0.000 | 0.001 |
| (C1-C14) | No tracer | 0.791 | 0.180 | 0.025 | 0.003 | 0.001 | 0.000 |
|  |  |  |  |  |  |  |  |
| C16:0  *m/z* 304 | [U-^13^C]Val | 0.793 | 0.180 | 0.023 | 0.003 | 0.000 | 0.000 |
| (C1-C13) | No tracer | 0.795 | 0.180 | 0.023 | 0.002 | 0.000 | 0.000 |

* [U-^13^C]valine labeling in the medium was 45%

**Table N.** Mass isotopomer distributions of intracellular metabolites for experiments with **[U-^13^C]propionate** and **no tracer** in presence/absence of **vitamin B_12_**, measured on day 7, i.e. after 24 h incubation with the tracer. Data was corrected for natural isotope abundances.

| Intracellular metab. *m/z* of fragment  (C-atoms) | Tracer | M0 | M1 | M2 | M3 | M4 | M5 | M6 |
| --- | --- | --- | --- | --- | --- | --- | --- | --- |
| Methylmalonate  *m/z* 289 | [U-^13^C]Prop+B_12_ | 0.400 | 0.012 | 0.042 | 0.527 | 0.018 |  |  |
| (C1-C4) | [U-^13^C]Prop | 0.297 | 0.032 | 0.075 | 0.545 | 0.052 |  |  |
|  | No tracer | 0.998 | 0.000 | 0.000 | 0.002 | 0.000 |  |  |
|  |  |  |  |  |  |  |  |  |
| Succinate  *m/z* 289 | [U-^13^C]Prop+B_12_ | 0.827 | 0.019 | 0.040 | 0.108 | 0.005 |  |  |
| (C1-C4) | [U-^13^C]Prop | 0.959 | 0.009 | 0.010 | 0.020 | 0.002 |  |  |
|  | No tracer | 0.993 | 0.005 | 0.000 | 0.000 | 0.001 |  |  |
|  |  |  |  |  |  |  |  |  |
| Malate  *m/z* 419 | [U-^13^C]Prop+B_12_ | 0.888 | 0.021 | 0.052 | 0.039 | 0.001 |  |  |
| (C1-C4) | [U-^13^C]Prop | 0.989 | 0.000 | 0.006 | 0.005 | 0.000 |  |  |
|  | No tracer | 1.000 | 0.000 | 0.000 | 0.000 | 0.000 |  |  |
|  |  |  |  |  |  |  |  |  |
| Citrate  *m/z* 459 | [U-^13^C]Prop+B_12_ | 0.882 | 0.019 | 0.063 | 0.028 | 0.003 | 0.001 | 0.002 |
| (C1-C6) | [U-^13^C]Prop | 0.982 | 0.000 | 0.013 | 0.004 | 0.000 | 0.000 | 0.002 |
|  | No tracer | 0.996 | 0.000 | 0.004 | 0.000 | 0.000 | 0.000 | 0.001 |
|  |  |  |  |  |  |  |  |  |
| α-ketoglutarate  *m/z* 346 | [U-^13^C]Prop+B_12_ | 0.855 | 0.026 | 0.093 | 0.020 | 0.003 | 0.001 |  |
| (C1-C5) | [U-^13^C]Prop | 0.974 | 0.000 | 0.020 | 0.004 | 0.001 | 0.001 |  |
|  | No tracer | 0.990 | 0.000 | 0.009 | 0.001 | 0.000 | 0.001 |  |

**Table O.** Mass isotopomer distributions of methyl-myristate (C14:0) fragment at *m/z* 242 (C1-C14, FAME derivatization) for parallel labeling experiments with [U-^13^C]valine, [U-^13^C]leucine, [U-^13^C]isoleucine, [U-^13^C]glutamine and no tracer measured on day 7, i.e. after 24 h incubation with the tracer (mean ± stdev, *n*=4 biological replicates). Data was not corrected for natural isotope abundances.

| *m/z* | No tracer | [U-^13^C]Val | [U-^13^C]Leu | [U-^13^C]Ile | [U-^13^C]Gln |
| --- | --- | --- | --- | --- | --- |
| 242 (M0) | 0.838 ± 0.002 | 0.839 ± 0.004 | 0.727 ± 0.005 | 0.779 ± 0.002 | 0.707 ± 0.009 |
| 243 (M1) | 0.143 ± 0.002 | 0.141 ± 0.004 | 0.158 ± 0.001 | 0.138 ± 0.002 | 0.128 ± 0.002 |
| 244 (M2) | 0.015 ± 0.000 | 0.016 ± 0.000 | 0.072 ± 0.004 | 0.063 ± 0.002 | 0.102 ± 0.004 |
| 245 (M3) | 0.001 ± 0.000 | 0.001 ± 0.000 | 0.022 ± 0.001 | 0.009 ± 0.000 | 0.018 ± 0.001 |
| 246 (M4) | 0.001 ± 0.001 | 0.000 ± 0.000 | 0.012 ± 0.001 | 0.007 ± 0.001 | 0.026 ± 0.002 |
| 247 (M5) | 0.000 ± 0.000 | 0.000 ± 0.000 | 0.004 ± 0.001 | 0.001 ± 0.000 | 0.005 ± 0.002 |
| 248 (M6) |  |  | 0.002 ± 0.000 | 0.001 ± 0.000 | 0.007 ± 0.001 |
| 249 (M7) |  |  | 0.001 ± 0.000 | 0.000 ± 0.000 | 0.002 ± 0.002 |
| 250 (M8) |  |  |  |  | 0.002 ± 0.001 |

* [U-^13^C]valine labeling in the medium was 45%
* [U-^13^C]leucine labeling in the medium was 42%
* [U-^13^C]isoleucine labeling in the medium was 47%
* [U-^13^C]glutamine labeling in the medium on day 6 (t=0) was 97%

**Table P.** Mass isotopomer distributions of methyl-myristate (C14:0) fragment at *m/z* 242 (C1-C14, FAME derivatization) for 4 experiments with **no tracer** measured on day 7 (mean, average of at least 2 injections for each sample). Data was not corrected for natural isotope abundances.

| *m/z* | Expt 1 | Expt 2 | Expt 3 | Expt 4 | Mean ± stdev |
| --- | --- | --- | --- | --- | --- |
| 242 (M0) | 0.837 | 0.836 | 0.837 | 0.841 | 0.838 ± 0.002 |
| 243 (M1) | 0.145 | 0.145 | 0.142 | 0.141 | 0.143 ± 0.002 |
| 244 (M2) | 0.015 | 0.016 | 0.015 | 0.015 | 0.015 ± 0.000 |
| 245 (M3) | 0.001 | 0.001 | 0.001 | 0.001 | 0.001 ± 0.000 |
| 246 (M4) | 0.000 | 0.000 | 0.001 | 0.000 | 0.001 ± 0.001 |
| 247 (M5) | 0.000 | 0.000 | 0.000 | 0.000 | 0.000 ± 0.000 |

**Table Q.** Mass isotopomer distributions of methyl-myristate (C14:0) fragment at *m/z* 242 (C1-C14, FAME derivatization) for 4 parallel labeling experiments with **[U-^13^C]valine** measured on day 7, i.e. after 24 h incubation with the tracer (mean, average of at least 2 injections for each sample). Data was not corrected for natural isotope abundances.

| *m/z* | Expt 1 | Expt 2 | Expt 3 | Expt 4 | Mean ± stdev |
| --- | --- | --- | --- | --- | --- |
| 242 (M0) | 0.837 | 0.834 | 0.843 | 0.842 | 0.839 ± 0.004 |
| 243 (M1) | 0.143 | 0.147 | 0.137 | 0.140 | 0.141 ± 0.004 |
| 244 (M2) | 0.015 | 0.016 | 0.015 | 0.015 | 0.016 ± 0.000 |
| 245 (M3) | 0.001 | 0.001 | 0.001 | 0.001 | 0.001 ± 0.000 |
| 246 (M4) | 0.000 | 0.000 | 0.001 | 0.000 | 0.000 ± 0.000 |
| 247 (M5) | 0.000 | 0.000 | 0.001 | 0.000 | 0.000 ± 0.000 |

* [U-^13^C]valine labeling in the medium was 45%

**Table R.** Mass isotopomer distributions of methyl-myristate (C14:0) fragment at *m/z* 242 (C1-C14, FAME derivatization) for 4 parallel labeling experiments with **[U-^13^C]leucine** measured on day 7, i.e. after 24 h incubation with the tracer (mean, average of at least 2 injections for each sample). Data was not corrected for natural isotope abundances.

| *m/z* | Expt 1 | Expt 2 | Expt 3 | Expt 4 | Mean ± stdev |
| --- | --- | --- | --- | --- | --- |
| 242 (M0) | 0.733 | 0.730 | 0.726 | 0.721 | 0.727 ± 0.005 |
| 243 (M1) | 0.157 | 0.160 | 0.157 | 0.158 | 0.158 ± 0.001 |
| 244 (M2) | 0.068 | 0.070 | 0.074 | 0.076 | 0.072 ± 0.004 |
| 245 (M3) | 0.020 | 0.021 | 0.021 | 0.024 | 0.022 ± 0.001 |
| 246 (M4) | 0.011 | 0.012 | 0.013 | 0.014 | 0.012 ± 0.001 |
| 247 (M5) | 0.003 | 0.004 | 0.004 | 0.004 | 0.004 ± 0.001 |
| 248 (M6) | 0.002 | 0.002 | 0.002 | 0.002 | 0.002 ± 0.000 |
| 249 (M7) | 0.001 | 0.001 | 0.001 | 0.001 | 0.001 ± 0.000 |

* [U-^13^C]leucine labeling in the medium was 42%

**Table S.** Mass isotopomer distributions of methyl-myristate (C14:0) fragment at *m/z* 242 (C1-C14, FAME derivatization) for 4 parallel labeling experiments with **[U-^13^C]isoleucine** measured on day 7, i.e. after 24 h incubation with the tracer (mean, average of at least 2 injections for each sample). Data was not corrected for natural isotope abundances.

| *m/z* | Expt 1 | Expt 2 | Expt 3 | Expt 4 | Mean ± stdev |
| --- | --- | --- | --- | --- | --- |
| 242 (M0) | 0.781 | 0.777 | 0.778 | 0.780 | 0.779 ± 0.002 |
| 243 (M1) | 0.138 | 0.141 | 0.136 | 0.138 | 0.138 ± 0.002 |
| 244 (M2) | 0.062 | 0.062 | 0.065 | 0.064 | 0.063 ± 0.002 |
| 245 (M3) | 0.009 | 0.009 | 0.009 | 0.009 | 0.009 ± 0.000 |
| 246 (M4) | 0.007 | 0.007 | 0.008 | 0.007 | 0.007 ± 0.001 |
| 247 (M5) | 0.001 | 0.001 | 0.002 | 0.001 | 0.001 ± 0.000 |
| 248 (M6) | 0.001 | 0.001 | 0.001 | 0.001 | 0.001 ± 0.000 |
| 249 (M7) | 0.000 | 0.001 | 0.001 | 0.000 | 0.000 ± 0.000 |

* [U-^13^C]isoleucine labeling in the medium was 47%

**Table T.** Mass isotopomer distributions of methyl-myristate (C14:0) fragment at *m/z* 242 (C1-C14, FAME derivatization) for 4 parallel labeling experiments with **[U-^13^C]glutamine** measured on day 7, i.e. after 24 h incubation with the tracer (mean, average of at least 2 injections for each sample). Data was not corrected for natural isotope abundances.

| *m/z* | Expt 1 | Expt 2 | Expt 3 | Expt 4 | Mean ± stdev |
| --- | --- | --- | --- | --- | --- |
| 242 (M0) | 0.712 | 0.699 | 0.700 | 0.717 | 0.707 ± 0.009 |
| 243 (M1) | 0.128 | 0.130 | 0.126 | 0.129 | 0.128 ± 0.002 |
| 244 (M2) | 0.098 | 0.100 | 0.108 | 0.100 | 0.102 ± 0.004 |
| 245 (M3) | 0.017 | 0.017 | 0.019 | 0.017 | 0.018 ± 0.001 |
| 246 (M4) | 0.026 | 0.026 | 0.030 | 0.024 | 0.026 ± 0.002 |
| 247 (M5) | 0.005 | 0.008 | 0.005 | 0.004 | 0.005 ± 0.002 |
| 248 (M6) | 0.007 | 0.008 | 0.007 | 0.005 | 0.007 ± 0.001 |
| 249 (M7) | 0.003 | 0.005 | 0.001 | 0.001 | 0.002 ± 0.002 |
| 250 (M8) | 0.002 | 0.003 | 0.002 | 0.001 | 0.002 ± 0.001 |

* [U-^13^C]glutamine labeling in the medium on day 6 (t=0) was 97%

**Table U.** Mass isotopomer distributions of methyl-pentadecanoate (C15:0) fragment at *m/z* 256 (C1-C15, FAME derivatization) for parallel labeling experiments with [U-^13^C]valine, [U-^13^C]leucine, [U-^13^C]isoleucine, [U-^13^C]glutamine and no tracer measured on day 7, i.e. after 24 h incubation with the tracer (mean ± stdev, *n*=4 biological replicates). Data was not corrected for natural isotope abundances.

| *m/z* | No tracer | [U-^13^C]Val | [U-^13^C]Leu | [U-^13^C]Ile | [U-^13^C]Gln |
| --- | --- | --- | --- | --- | --- |
| 256 (M0) | 0.822 ± 0.005 | 0.754 ± 0.005 | 0.646 ± 0.005 | 0.605 ± 0.002 | 0.640 ± 0.010 |
| 257 (M1) | 0.157 ± 0.003 | 0.143 ± 0.004 | 0.177 ± 0.003 | 0.121 ± 0.003 | 0.132 ± 0.002 |
| 258 (M2) | 0.017 ± 0.001 | 0.027 ± 0.001 | 0.111 ± 0.002 | 0.082 ± 0.001 | 0.144 ± 0.004 |
| 259 (M3) | 0.001 ± 0.000 | 0.064 ± 0.002 | 0.035 ± 0.001 | 0.129 ± 0.002 | 0.027 ± 0.001 |
| 260 (M4) | 0.001 ± 0.001 | 0.010 ± 0.000 | 0.020 ± 0.001 | 0.030 ± 0.000 | 0.037 ± 0.004 |
| 261 (M5) | 0.000 ± 0.000 | 0.001 ± 0.000 | 0.006 ± 0.000 | 0.025 ± 0.001 | 0.006 ± 0.001 |
| 262 (M6) |  |  | 0.003 ± 0.000 | 0.004 ± 0.000 | 0.008 ± 0.001 |
| 263 (M7) |  |  | 0.001 ± 0.000 | 0.003 ± 0.000 | 0.001 ± 0.000 |
| 264 (M8) |  |  |  |  | 0.002 ± 0.000 |

* [U-^13^C]valine labeling in the medium was 45%
* [U-^13^C]leucine labeling in the medium was 42%
* [U-^13^C]isoleucine labeling in the medium was 47%
* [U-^13^C]glutamine labeling in the medium on day 6 (t=0) was 97%

**Table V.** Mass isotopomer distributions of methyl-pentadecanoate (C15:0) fragment at *m/z* 256 (C1-C15, FAME derivatization) for 4 experiments with **no tracer** measured on day 7 (mean, average of at least 2 injections for each sample). Data was not corrected for natural isotope abundances.

| *m/z* | Expt 1 | Expt 2 | Expt 3 | Expt 4 | Mean ± stdev |
| --- | --- | --- | --- | --- | --- |
| 256 (M0) | 0.818 | 0.818 | 0.824 | 0.828 | 0.822 ± 0.005 |
| 257 (M1) | 0.158 | 0.159 | 0.156 | 0.153 | 0.157 ± 0.003 |
| 258 (M2) | 0.018 | 0.018 | 0.017 | 0.017 | 0.017 ± 0.001 |
| 259 (M3) | 0.002 | 0.001 | 0.001 | 0.001 | 0.001 ± 0.000 |
| 260 (M4) | 0.002 | 0.001 | 0.001 | 0.000 | 0.001 ± 0.001 |
| 261 (M5) | 0.001 | 0.000 | 0.000 | 0.000 | 0.000 ± 0.000 |

**Table W.** Mass isotopomer distributions of methyl-pentadecanoate (C15:0) fragment at *m/z* 256 (C1-C15, FAME derivatization) for 4 parallel labeling experiments with **[U-^13^C]valine** measured on day 7, i.e. after 24 h incubation with the tracer (mean, average of at least 2 injections for each sample). Data was not corrected for natural isotope abundances.

| *m/z* | Expt 1 | Expt 2 | Expt 3 | Expt 4 | Mean ± stdev |
| --- | --- | --- | --- | --- | --- |
| 256 (M0) | 0.754 | 0.747 | 0.755 | 0.759 | 0.754 ± 0.005 |
| 257 (M1) | 0.142 | 0.149 | 0.139 | 0.141 | 0.143 ± 0.004 |
| 258 (M2) | 0.027 | 0.029 | 0.027 | 0.027 | 0.027 ± 0.001 |
| 259 (M3) | 0.064 | 0.063 | 0.066 | 0.063 | 0.064 ± 0.002 |
| 260 (M4) | 0.010 | 0.010 | 0.010 | 0.009 | 0.010 ± 0.000 |
| 261 (M5) | 0.001 | 0.001 | 0.001 | 0.001 | 0.001 ± 0.000 |

* [U-^13^C]valine labeling in the medium was 45%

**Table X.** Mass isotopomer distributions of methyl-pentadecanoate (C15:0) fragment at *m/z* 256 (C1-C15, FAME derivatization) for 4 parallel labeling experiments with **[U-^13^C]leucine** measured on day 7, i.e. after 24 h incubation with the tracer (mean, average of at least 2 injections for each sample). Data was not corrected for natural isotope abundances.

| *m/z* | Expt 1 | Expt 2 | Expt 3 | Expt 4 | Mean ± stdev |
| --- | --- | --- | --- | --- | --- |
| 256 (M0) | 0.644 | 0.646 | 0.652 | 0.641 | 0.646 ± 0.005 |
| 257 (M1) | 0.178 | 0.182 | 0.174 | 0.175 | 0.177 ± 0.003 |
| 258 (M2) | 0.111 | 0.109 | 0.110 | 0.114 | 0.111 ± 0.002 |
| 259 (M3) | 0.035 | 0.035 | 0.034 | 0.037 | 0.035 ± 0.001 |
| 260 (M4) | 0.021 | 0.020 | 0.019 | 0.022 | 0.020 ± 0.001 |
| 261 (M5) | 0.006 | 0.006 | 0.006 | 0.007 | 0.006 ± 0.000 |
| 262 (M6) | 0.003 | 0.003 | 0.002 | 0.003 | 0.003 ± 0.000 |
| 263 (M7) | 0.001 | 0.001 | 0.001 | 0.001 | 0.001 ± 0.000 |

* [U-^13^C]leucine labeling in the medium was 42%

**Table Y.** Mass isotopomer distributions of methyl-pentadecanoate (C15:0) fragment at *m/z* 256 (C1-C15, FAME derivatization) for 4 parallel labeling experiments with **[U-^13^C]isoleucine** measured on day 7, i.e. after 24 h incubation with the tracer (mean, average of at least 2 injections for each sample). Data was not corrected for natural isotope abundances.

| *m/z* | Expt 1 | Expt 2 | Expt 3 | Expt 4 | Mean ± stdev |
| --- | --- | --- | --- | --- | --- |
| 256 (M0) | 0.606 | 0.604 | 0.603 | 0.606 | 0.605 ± 0.002 |
| 257 (M1) | 0.122 | 0.124 | 0.118 | 0.120 | 0.121 ± 0.003 |
| 258 (M2) | 0.081 | 0.081 | 0.083 | 0.082 | 0.082 ± 0.001 |
| 259 (M3) | 0.127 | 0.128 | 0.131 | 0.129 | 0.129 ± 0.002 |
| 260 (M4) | 0.030 | 0.030 | 0.031 | 0.030 | 0.030 ± 0.000 |
| 261 (M5) | 0.025 | 0.024 | 0.026 | 0.025 | 0.025 ± 0.001 |
| 262 (M6) | 0.004 | 0.004 | 0.004 | 0.004 | 0.004 ± 0.000 |
| 263 (M7) | 0.003 | 0.003 | 0.003 | 0.003 | 0.003 ± 0.000 |

* [U-^13^C]isoleucine labeling in the medium was 47%

**Table Z.** Mass isotopomer distributions of methyl-pentadecanoate (C15:0) fragment at *m/z* 256 (C1-C15, FAME derivatization) for 4 parallel labeling experiments with **[U-^13^C]glutamine** measured on day 7, i.e. after 24 h incubation with the tracer (mean, average of at least 2 injections for each sample). Data was not corrected for natural isotope abundances.

| *m/z* | Expt 1 | Expt 2 | Expt 3 | Expt 4 | Mean ± stdev |
| --- | --- | --- | --- | --- | --- |
| 256 (M0) | 0.641 | 0.632 | 0.635 | 0.654 | 0.640 ± 0.010 |
| 257 (M1) | 0.132 | 0.134 | 0.129 | 0.132 | 0.132 ± 0.002 |
| 258 (M2) | 0.143 | 0.142 | 0.150 | 0.141 | 0.144 ± 0.004 |
| 259 (M3) | 0.027 | 0.027 | 0.028 | 0.026 | 0.027 ± 0.001 |
| 260 (M4) | 0.037 | 0.041 | 0.038 | 0.032 | 0.037 ± 0.004 |
| 261 (M5) | 0.006 | 0.008 | 0.006 | 0.005 | 0.006 ± 0.001 |
| 262 (M6) | 0.008 | 0.009 | 0.009 | 0.007 | 0.008 ± 0.001 |
| 263 (M7) | 0.001 | 0.001 | 0.001 | 0.001 | 0.001 ± 0.000 |
| 264 (M8) | 0.002 | 0.002 | 0.002 | 0.001 | 0.002 ± 0.000 |

* [U-^13^C]glutamine labeling in the medium on day 6 (t=0) was 97%

**Table AA.** Mass isotopomer distributions of methyl-palmitate (C16:0) fragment at *m/z* 270 (C1-C16, FAME derivatization) for parallel labeling experiments with [U-^13^C]valine, [U-^13^C]leucine, [U-^13^C]isoleucine, [U-^13^C]glutamine and no tracer measured on day 7, i.e. after 24 h incubation with the tracer (mean ± stdev, *n*=4 biological replicates). Data was not corrected for natural isotope abundances.

| *m/z* | No tracer | [U-^13^C]Val | [U-^13^C]Leu | [U-^13^C]Ile | [U-^13^C]Gln |
| --- | --- | --- | --- | --- | --- |
| 270 (M0) | 0.815 ± 0.003 | 0.816 ± 0.004 | 0.682 ± 0.006 | 0.745 ± 0.001 | 0.662 ± 0.007 |
| 271 (M1) | 0.163 ± 0.003 | 0.162 ± 0.003 | 0.174 ± 0.002 | 0.155 ± 0.002 | 0.140 ± 0.002 |
| 272 (M2) | 0.019 ± 0.001 | 0.020 ± 0.001 | 0.085 ± 0.003 | 0.074 ± 0.002 | 0.116 ± 0.003 |
| 273 (M3) | 0.002 ± 0.000 | 0.002 ± 0.000 | 0.029 ± 0.002 | 0.013 ± 0.000 | 0.023 ± 0.001 |
| 274 (M4) |  |  | 0.018 ± 0.001 | 0.009 ± 0.000 | 0.034 ± 0.002 |
| 275 (M5) |  |  | 0.006 ± 0.000 | 0.001 ± 0.000 | 0.006 ± 0.000 |
| 276 (M6) |  |  | 0.003 ± 0.000 | 0.001 ± 0.000 | 0.010 ± 0.001 |
| 277 (M7) |  |  | 0.001 ± 0.000 |  | 0.002 ± 0.000 |
| 278 (M8) |  |  |  |  | 0.003 ± 0.000 |

* [U-^13^C]valine labeling in the medium was 45%
* [U-^13^C]leucine labeling in the medium was 42%
* [U-^13^C]isoleucine labeling in the medium was 47%
* [U-^13^C]glutamine labeling in the medium on day 6 (t=0) was 97%

**Table AB.** Mass isotopomer distributions of methyl-palmitate (C16:0) fragment at *m/z* 270 (C1-C16, FAME derivatization) for 4 experiments with **no tracer** measured on day 7 (mean, average of at least 2 injections for each sample). Data was not corrected for natural isotope abundances.

| *m/z* | Expt 1 | Expt 2 | Expt 3 | Expt 4 | Mean ± stdev |
| --- | --- | --- | --- | --- | --- |
| 270 (M0) | 0.812 | 0.812 | 0.816 | 0.819 | 0.815 ± 0.003 |
| 271 (M1) | 0.165 | 0.166 | 0.162 | 0.160 | 0.163 ± 0.003 |
| 272 (M2) | 0.019 | 0.019 | 0.018 | 0.018 | 0.019 ± 0.001 |
| 273 (M3) | 0.002 | 0.002 | 0.002 | 0.002 | 0.002 ± 0.000 |

**Table AC.** Mass isotopomer distributions of methyl-palmitate (C16:0) fragment at *m/z* 270 (C1-C16, FAME derivatization) for 4 parallel labeling experiments with **[U-^13^C]valine** measured on day 7, i.e. after 24 h incubation with the tracer (mean, average of at least 2 injections for each sample). Data was not corrected for natural isotope abundances.

| *m/z* | Expt 1 | Expt 2 | Expt 3 | Expt 4 | Mean ± stdev |
| --- | --- | --- | --- | --- | --- |
| 270 (M0) | 0.815 | 0.811 | 0.819 | 0.819 | 0.816 ± 0.004 |
| 271 (M1) | 0.163 | 0.166 | 0.159 | 0.160 | 0.162 ± 0.003 |
| 272 (M2) | 0.020 | 0.020 | 0.020 | 0.019 | 0.020 ± 0.001 |
| 273 (M3) | 0.002 | 0.002 | 0.002 | 0.002 | 0.002 ± 0.000 |

* [U-^13^C]valine labeling in the medium was 45%

**Table AD.** Mass isotopomer distributions of methyl-palmitate (C16:0) fragment at *m/z* 270 (C1-C16, FAME derivatization) for 4 parallel labeling experiments with **[U-^13^C]leucine** measured on day 7, i.e. after 24 h incubation with the tracer (mean, average of at least 2 injections for each sample). Data was not corrected for natural isotope abundances.

| *m/z* | Expt 1 | Expt 2 | Expt 3 | Expt 4 | Mean ± stdev |
| --- | --- | --- | --- | --- | --- |
| 270 (M0) | 0.688 | 0.684 | 0.682 | 0.674 | 0.682 ± 0.006 |
| 271 (M1) | 0.174 | 0.177 | 0.173 | 0.173 | 0.174 ± 0.002 |
| 272 (M2) | 0.082 | 0.083 | 0.086 | 0.089 | 0.085 ± 0.003 |
| 273 (M3) | 0.028 | 0.028 | 0.029 | 0.031 | 0.029 ± 0.002 |
| 274 (M4) | 0.017 | 0.017 | 0.017 | 0.019 | 0.018 ± 0.001 |
| 275 (M5) | 0.006 | 0.006 | 0.006 | 0.007 | 0.006 ± 0.000 |
| 276 (M6) | 0.003 | 0.003 | 0.003 | 0.004 | 0.003 ± 0.000 |
| 277 (M7) | 0.001 | 0.001 | 0.001 | 0.001 | 0.001 ± 0.000 |

* [U-^13^C]leucine labeling in the medium was 42%

**Table AE.** Mass isotopomer distributions of methyl-palmitate (C16:0) fragment at *m/z* 270 (C1-C16, FAME derivatization) for 4 parallel labeling experiments with **[U-^13^C]isoleucine** measured on day 7, i.e. after 24 h incubation with the tracer (mean, average of at least 2 injections for each sample). Data was not corrected for natural isotope abundances.

| *m/z* | Expt 1 | Expt 2 | Expt 3 | Expt 4 | Mean ± stdev |
| --- | --- | --- | --- | --- | --- |
| 270 (M0) | 0.746 | 0.746 | 0.744 | 0.745 | 0.745 ± 0.001 |
| 271 (M1) | 0.156 | 0.157 | 0.153 | 0.153 | 0.155 ± 0.002 |
| 272 (M2) | 0.073 | 0.072 | 0.076 | 0.076 | 0.074 ± 0.002 |
| 273 (M3) | 0.013 | 0.012 | 0.013 | 0.013 | 0.013 ± 0.000 |
| 274 (M4) | 0.009 | 0.009 | 0.009 | 0.009 | 0.009 ± 0.000 |
| 275 (M5) | 0.001 | 0.001 | 0.002 | 0.002 | 0.001 ± 0.000 |
| 276 (M6) | 0.001 | 0.001 | 0.001 | 0.001 | 0.001 ± 0.000 |

* [U-^13^C]isoleucine labeling in the medium was 47%

**Table AF.** Mass isotopomer distributions of methyl-palmitate (C16:0) fragment at *m/z* 270 (C1-C16, FAME derivatization) for 4 parallel labeling experiments with **[U-^13^C]glutamine** measured on day 7, i.e. after 24 h incubation with the tracer (mean, average of at least 2 injections for each sample). Data was not corrected for natural isotope abundances.

| *m/z* | Expt 1 | Expt 2 | Expt 3 | Expt 4 | Mean ± stdev |
| --- | --- | --- | --- | --- | --- |
| 270 (M0) | 0.661 | 0.662 | 0.655 | 0.671 | 0.662 ± 0.007 |
| 271 (M1) | 0.140 | 0.142 | 0.139 | 0.140 | 0.140 ± 0.002 |
| 272 (M2) | 0.115 | 0.115 | 0.120 | 0.114 | 0.116 ± 0.003 |
| 273 (M3) | 0.023 | 0.023 | 0.025 | 0.023 | 0.023 ± 0.001 |
| 274 (M4) | 0.034 | 0.033 | 0.036 | 0.032 | 0.034 ± 0.002 |
| 275 (M5) | 0.006 | 0.006 | 0.007 | 0.006 | 0.006 ± 0.000 |
| 276 (M6) | 0.010 | 0.009 | 0.011 | 0.009 | 0.010 ± 0.001 |
| 277 (M7) | 0.002 | 0.002 | 0.002 | 0.001 | 0.002 ± 0.000 |
| 278 (M8) | 0.003 | 0.003 | 0.003 | 0.003 | 0.003 ± 0.000 |

* [U-^13^C]glutamine labeling in the medium on day 6 (t=0) was 97%

**Table AG.** Mass isotopomer distributions of methyl-heptadecanoate (C17:0) fragment at *m/z* 284 (C1-C17, FAME derivatization) for parallel labeling experiments with [U-^13^C]valine, [U-^13^C]leucine, [U-^13^C]isoleucine, [U-^13^C]glutamine and no tracer measured on day 7, i.e. after 24 h incubation with the tracer (mean ± stdev, *n*=4 biological replicates). Data was not corrected for natural isotope abundances.

| *m/z* | No tracer | [U-^13^C]Val | [U-^13^C]Leu | [U-^13^C]Ile | [U-^13^C]Gln |
| --- | --- | --- | --- | --- | --- |
| 284 (M0) | 0.814 ± 0.001 | 0.737 ± 0.003 | 0.597 ± 0.004 | 0.568 ± 0.003 | 0.589 ± 0.008 |
| 285 (M1) | 0.163 ± 0.001 | 0.148 ± 0.001 | 0.182 ± 0.001 | 0.122 ± 0.001 | 0.131 ± 0.002 |
| 286 (M2) | 0.019 ± 0.001 | 0.030 ± 0.001 | 0.129 ± 0.002 | 0.094 ± 0.001 | 0.167 ± 0.003 |
| 287 (M3) | 0.002 ± 0.000 | 0.070 ± 0.002 | 0.046 ± 0.001 | 0.139 ± 0.002 | 0.034 ± 0.001 |
| 288 (M4) | 0.000 ± 0.000 | 0.012 ± 0.001 | 0.028 ± 0.001 | 0.036 ± 0.000 | 0.047 ± 0.003 |
| 289 (M5) | 0.000 ± 0.000 | 0.002 ± 0.000 | 0.010 ± 0.000 | 0.029 ± 0.001 | 0.009 ± 0.001 |
| 290 (M6) |  |  | 0.005 ± 0.000 | 0.006 ± 0.000 | 0.013 ± 0.002 |
| 291 (M7) |  |  | 0.002 ± 0.000 | 0.004 ± 0.000 | 0.002 ± 0.000 |
| 292 (M8) |  |  | 0.001 ± 0.000 | 0.001 ± 0.000 | 0.004 ± 0.001 |

* [U-^13^C]valine labeling in the medium was 45%
* [U-^13^C]leucine labeling in the medium was 42%
* [U-^13^C]isoleucine labeling in the medium was 47%
* [U-^13^C]glutamine labeling in the medium on day 6 (t=0) was 97%

**Table AH.** Mass isotopomer distributions of methyl-heptadecanoate (C17:0) fragment at *m/z* 284 (C1-C17, FAME derivatization) for 4 experiments with **no tracer** measured on day 7 (mean, average of at least 2 injections for each sample). Data was not corrected for natural isotope abundances.

| *m/z* | Expt 1 | Expt 2 | Expt 3 | Expt 4 | Mean ± stdev |
| --- | --- | --- | --- | --- | --- |
| 284 (M0) | 0.813 | 0.813 | 0.813 | 0.816 | 0.814 ± 0.001 |
| 285 (M1) | 0.165 | 0.164 | 0.163 | 0.162 | 0.163 ± 0.001 |
| 286 (M2) | 0.019 | 0.019 | 0.018 | 0.019 | 0.019 ± 0.001 |
| 287 (M3) | 0.002 | 0.002 | 0.002 | 0.002 | 0.002 ± 0.000 |
| 288 (M4) | 0.000 | 0.000 | 0.000 | 0.000 | 0.000 ± 0.000 |
| 289 (M5) | 0.000 | 0.000 | 0.000 | 0.000 | 0.000 ± 0.000 |

**Table AI.** Mass isotopomer distributions of methyl-heptadecanoate (C17:0) fragment at *m/z* 284 (C1-C17, FAME derivatization) for 4 parallel labeling experiments with **[U-^13^C]valine** measured on day 7, i.e. after 24 h incubation with the tracer (mean, average of at least 2 injections for each sample). Data was not corrected for natural isotope abundances.

| *m/z* | Expt 1 | Expt 2 | Expt 3 | Expt 4 | Mean ± stdev |
| --- | --- | --- | --- | --- | --- |
| 284 (M0) | 0.734 | 0.737 | 0.735 | 0.740 | 0.737 ± 0.003 |
| 285 (M1) | 0.148 | 0.149 | 0.146 | 0.149 | 0.148 ± 0.001 |
| 286 (M2) | 0.030 | 0.031 | 0.029 | 0.030 | 0.030 ± 0.001 |
| 287 (M3) | 0.071 | 0.068 | 0.072 | 0.067 | 0.070 ± 0.002 |
| 288 (M4) | 0.011 | 0.011 | 0.013 | 0.011 | 0.012 ± 0.001 |
| 289 (M5) | 0.001 | 0.001 | 0.002 | 0.001 | 0.002 ± 0.000 |

* [U-^13^C]valine labeling in the medium was 45%

**Table AJ.** Mass isotopomer distributions of methyl-heptadecanoate (C17:0) fragment at *m/z* 284 (C1-C17, FAME derivatization) for 4 parallel labeling experiments with **[U-^13^C]leucine** measured on day 7, i.e. after 24 h incubation with the tracer (mean, average of at least 2 injections for each sample). Data was not corrected for natural isotope abundances.

| *m/z* | Expt 1 | Expt 2 | Expt 3 | Expt 4 | Mean ± stdev |
| --- | --- | --- | --- | --- | --- |
| 284 (M0) | 0.595 | 0.601 | 0.599 | 0.592 | 0.597 ± 0.004 |
| 285 (M1) | 0.180 | 0.183 | 0.181 | 0.181 | 0.182 ± 0.001 |
| 286 (M2) | 0.130 | 0.127 | 0.128 | 0.130 | 0.129 ± 0.002 |
| 287 (M3) | 0.047 | 0.045 | 0.045 | 0.048 | 0.046 ± 0.001 |
| 288 (M4) | 0.028 | 0.027 | 0.027 | 0.030 | 0.028 ± 0.001 |
| 289 (M5) | 0.010 | 0.010 | 0.010 | 0.011 | 0.010 ± 0.000 |
| 290 (M6) | 0.006 | 0.005 | 0.005 | 0.005 | 0.005 ± 0.000 |
| 291 (M7) | 0.002 | 0.002 | 0.002 | 0.002 | 0.002 ± 0.000 |

* [U-^13^C]leucine labeling in the medium was 42%

**Table AK.** Mass isotopomer distributions of methyl-heptadecanoate (C17:0) fragment at *m/z* 284 (C1-C17, FAME derivatization) for 4 parallel labeling experiments with **[U-^13^C]isoleucine** measured on day 7, i.e. after 24 h incubation with the tracer (mean, average of at least 2 injections for each sample). Data was not corrected for natural isotope abundances.

| *m/z* | Expt 1 | Expt 2 | Expt 3 | Expt 4 | Mean ± stdev |
| --- | --- | --- | --- | --- | --- |
| 284 (M0) | 0.566 | 0.571 | 0.565 | 0.570 | 0.568 ± 0.003 |
| 285 (M1) | 0.121 | 0.122 | 0.121 | 0.124 | 0.122 ± 0.001 |
| 286 (M2) | 0.095 | 0.093 | 0.094 | 0.094 | 0.094 ± 0.001 |
| 287 (M3) | 0.140 | 0.139 | 0.141 | 0.137 | 0.139 ± 0.002 |
| 288 (M4) | 0.036 | 0.035 | 0.036 | 0.035 | 0.036 ± 0.000 |
| 289 (M5) | 0.030 | 0.028 | 0.029 | 0.029 | 0.029 ± 0.001 |
| 290 (M6) | 0.006 | 0.006 | 0.006 | 0.006 | 0.006 ± 0.000 |
| 291 (M7) | 0.004 | 0.004 | 0.005 | 0.004 | 0.004 ± 0.000 |

* [U-^13^C]isoleucine labeling in the medium was 47%

**Table AL.** Mass isotopomer distributions of methyl-heptadecanoate (C17:0) fragment at *m/z* 284 (C1-C17, FAME derivatization) for 4 parallel labeling experiments with **[U-^13^C]glutamine** measured on day 7, i.e. after 24 h incubation with the tracer (mean, average of at least 2 injections for each sample). Data was not corrected for natural isotope abundances.

| *m/z* | Expt 1 | Expt 2 | Expt 3 | Expt 4 | Mean ± stdev |
| --- | --- | --- | --- | --- | --- |
| 284 (M0) | 0.583 | 0.590 | 0.583 | 0.599 | 0.589 ± 0.008 |
| 285 (M1) | 0.129 | 0.132 | 0.130 | 0.134 | 0.131 ± 0.002 |
| 286 (M2) | 0.170 | 0.167 | 0.167 | 0.163 | 0.167 ± 0.003 |
| 287 (M3) | 0.034 | 0.034 | 0.034 | 0.033 | 0.034 ± 0.001 |
| 288 (M4) | 0.049 | 0.046 | 0.051 | 0.044 | 0.047 ± 0.003 |
| 289 (M5) | 0.009 | 0.009 | 0.009 | 0.008 | 0.009 ± 0.001 |
| 290 (M6) | 0.015 | 0.013 | 0.014 | 0.011 | 0.013 ± 0.002 |
| 291 (M7) | 0.002 | 0.002 | 0.003 | 0.002 | 0.002 ± 0.000 |
| 292 (M8) | 0.005 | 0.004 | 0.005 | 0.004 | 0.004 ± 0.001 |

* [U-^13^C]glutamine labeling in the medium on day 6 (t=0) was 97%

**Table AM.** Mass isotopomer distributions of methyl-stearate (C18:0) fragment at *m/z* 298 (C1-C18, FAME derivatization) for parallel labeling experiments with [U-^13^C]valine, [U-^13^C]leucine, [U-^13^C]isoleucine, [U-^13^C]glutamine and no tracer measured on day 7, i.e. after 24 h incubation with the tracer (mean ± stdev, *n*=4 biological replicates). Data was not corrected for natural isotope abundances.

| *m/z* | No tracer | [U-^13^C]Val | [U-^13^C]Leu | [U-^13^C]Ile | [U-^13^C]Gln |
| --- | --- | --- | --- | --- | --- |
| 298 (M0) | 0.806 ± 0.002 | 0.807 ± 0.000 | 0.731 ± 0.005 | 0.771 ± 0.002 | 0.713 ± 0.006 |
| 299 (M1) | 0.170 ± 0.001 | 0.167 ± 0.002 | 0.173 ± 0.001 | 0.164 ± 0.001 | 0.153 ± 0.003 |
| 300 (M2) | 0.020 ± 0.000 | 0.020 ± 0.001 | 0.055 ± 0.002 | 0.048 ± 0.001 | 0.071 ± 0.002 |
| 301 (M3) | 0.002 ± 0.000 | 0.002 ± 0.000 | 0.018 ± 0.001 | 0.008 ± 0.000 | 0.014 ± 0.001 |
| 302 (M4) | 0.000 ± 0.000 | 0.001 ± 0.000 | 0.011 ± 0.001 | 0.005 ± 0.001 | 0.022 ± 0.001 |
| 303 (M5) | 0.000 ± 0.000 | 0.000 ± 0.000 | 0.005 ± 0.000 | 0.001 ± 0.000 | 0.005 ± 0.001 |
| 304 (M6) |  |  | 0.003 ± 0.000 | 0.001 ± 0.000 | 0.009 ± 0.001 |
| 305 (M7) |  |  | 0.001 ± 0.000 | 0.001 ± 0.000 | 0.002 ± 0.000 |
| 306 (M8) |  |  | 0.001 ± 0.000 | 0.000 ± 0.000 | 0.005 ± 0.001 |

* [U-^13^C]valine labeling in the medium was 45%
* [U-^13^C]leucine labeling in the medium was 42%
* [U-^13^C]isoleucine labeling in the medium was 47%
* [U-^13^C]glutamine labeling in the medium on day 6 (t=0) was 97%

**Table AN.** Mass isotopomer distributions of methyl-stearate (C18:0) fragment at *m/z* 298 (C1-C18, FAME derivatization) for 4 experiments with **no tracer** measured on day 7 (mean, average of at least 2 injections for each sample). Data was not corrected for natural isotope abundances.

| *m/z* | Expt 1 | Expt 2 | Expt 3 | Expt 4 | Mean ± stdev |
| --- | --- | --- | --- | --- | --- |
| 298 (M0) | 0.807 | 0.806 | 0.803 | 0.807 | 0.806 ± 0.002 |
| 299 (M1) | 0.170 | 0.171 | 0.169 | 0.168 | 0.170 ± 0.001 |
| 300 (M2) | 0.020 | 0.021 | 0.019 | 0.020 | 0.020 ± 0.000 |
| 301 (M3) | 0.002 | 0.002 | 0.002 | 0.002 | 0.002 ± 0.000 |
| 302 (M4) | 0.000 | 0.000 | 0.001 | 0.000 | 0.000 ± 0.000 |
| 303 (M5) | 0.000 | 0.000 | 0.000 | 0.000 | 0.000 ± 0.000 |

**Table AO.** Mass isotopomer distributions of methyl-stearate (C18:0) fragment at *m/z* 298 (C1-C18, FAME derivatization) for 4 parallel labeling experiments with **[U-^13^C]valine** measured on day 7, i.e. after 24 h incubation with the tracer (mean, average of at least 2 injections for each sample). Data was not corrected for natural isotope abundances.

| *m/z* | Expt 1 | Expt 2 | Expt 3 | Expt 4 | Mean ± stdev |
| --- | --- | --- | --- | --- | --- |
| 298 (M0) | 0.806 | 0.806 | 0.807 | 0.807 | 0.807 ± 0.000 |
| 299 (M1) | 0.167 | 0.169 | 0.165 | 0.168 | 0.167 ± 0.002 |
| 300 (M2) | 0.021 | 0.020 | 0.021 | 0.020 | 0.020 ± 0.001 |
| 301 (M3) | 0.002 | 0.002 | 0.002 | 0.002 | 0.002 ± 0.000 |
| 302 (M4) | 0.001 | 0.000 | 0.001 | 0.001 | 0.001 ± 0.000 |
| 303 (M5) | 0.000 | 0.000 | 0.001 | 0.000 | 0.000 ± 0.000 |

* [U-^13^C]valine labeling in the medium was 45%

**Table AP.** Mass isotopomer distributions of methyl-stearate (C18:0) fragment at *m/z* 298 (C1-C18, FAME derivatization) for 4 parallel labeling experiments with **[U-^13^C]leucine** measured on day 7, i.e. after 24 h incubation with the tracer (mean, average of at least 2 injections for each sample). Data was not corrected for natural isotope abundances.

| *m/z* | Expt 1 | Expt 2 | Expt 3 | Expt 4 | Mean ± stdev |
| --- | --- | --- | --- | --- | --- |
| 298 (M0) | 0.736 | 0.734 | 0.724 | 0.731 | 0.731 ± 0.005 |
| 299 (M1) | 0.172 | 0.172 | 0.174 | 0.173 | 0.173 ± 0.001 |
| 300 (M2) | 0.053 | 0.055 | 0.057 | 0.054 | 0.055 ± 0.002 |
| 301 (M3) | 0.017 | 0.018 | 0.019 | 0.018 | 0.018 ± 0.001 |
| 302 (M4) | 0.011 | 0.011 | 0.012 | 0.012 | 0.011 ± 0.001 |
| 303 (M5) | 0.005 | 0.004 | 0.005 | 0.005 | 0.005 ± 0.000 |
| 304 (M6) | 0.002 | 0.002 | 0.003 | 0.003 | 0.003 ± 0.000 |
| 305 (M7) | 0.001 | 0.001 | 0.002 | 0.002 | 0.001 ± 0.000 |

* [U-^13^C]leucine labeling in the medium was 42%

**Table AQ.** Mass isotopomer distributions of methyl-stearate (C18:0) fragment at *m/z* 298 (C1-C18, FAME derivatization) for 4 parallel labeling experiments with **[U-^13^C]isoleucine** measured on day 7, i.e. after 24 h incubation with the tracer (mean, average of at least 2 injections for each sample). Data was not corrected for natural isotope abundances.

| *m/z* | Expt 1 | Expt 2 | Expt 3 | Expt 4 | Mean ± stdev |
| --- | --- | --- | --- | --- | --- |
| 298 (M0) | 0.771 | 0.773 | 0.768 | 0.771 | 0.771 ± 0.002 |
| 299 (M1) | 0.166 | 0.164 | 0.163 | 0.164 | 0.164 ± 0.001 |
| 300 (M2) | 0.047 | 0.048 | 0.049 | 0.049 | 0.048 ± 0.001 |
| 301 (M3) | 0.007 | 0.008 | 0.008 | 0.008 | 0.008 ± 0.000 |
| 302 (M4) | 0.005 | 0.006 | 0.006 | 0.005 | 0.005 ± 0.001 |
| 303 (M5) | 0.001 | 0.001 | 0.001 | 0.001 | 0.001 ± 0.000 |
| 304 (M6) | 0.001 | 0.001 | 0.001 | 0.001 | 0.001 ± 0.000 |
| 305 (M7) | 0.001 | 0.000 | 0.001 | 0.000 | 0.001 ± 0.000 |

* [U-^13^C]isoleucine labeling in the medium was 47%

**Table AR.** Mass isotopomer distributions of methyl-stearate (C18:0) fragment at *m/z* 298 (C1-C18, FAME derivatization) for 4 parallel labeling experiments with **[U-^13^C]glutamine** measured on day 7, i.e. after 24 h incubation with the tracer (mean, average of at least 2 injections for each sample). Data was not corrected for natural isotope abundances.

| *m/z* | Expt 1 | Expt 2 | Expt 3 | Expt 4 | Mean ± stdev |
| --- | --- | --- | --- | --- | --- |
| 298 (M0) | 0.706 | 0.719 | 0.712 | 0.717 | 0.713 ± 0.006 |
| 299 (M1) | 0.157 | 0.154 | 0.149 | 0.152 | 0.153 ± 0.003 |
| 300 (M2) | 0.071 | 0.069 | 0.073 | 0.072 | 0.071 ± 0.002 |
| 301 (M3) | 0.014 | 0.013 | 0.014 | 0.014 | 0.014 ± 0.001 |
| 302 (M4) | 0.022 | 0.021 | 0.024 | 0.022 | 0.022 ± 0.001 |
| 303 (M5) | 0.005 | 0.004 | 0.006 | 0.004 | 0.005 ± 0.001 |
| 304 (M6) | 0.009 | 0.008 | 0.010 | 0.009 | 0.009 ± 0.001 |
| 305 (M7) | 0.002 | 0.002 | 0.002 | 0.002 | 0.002 ± 0.000 |
| 306 (M8) | 0.007 | 0.006 | 0.005 | 0.004 | 0.005 ± 0.001 |

* [U-^13^C]glutamine labeling in the medium on day 6 (t=0) was 97%

**Table AS.** ISA models for even chain fatty acids.

| *C14:0 model* | *C16:0 model* | *C18:0 model* |
| --- | --- | --- |
| AcCoA.Gln > AcCoA | AcCoA.Gln > AcCoA | AcCoA.Gln > AcCoA |
| AcCoA.GlnM1 > AcCoA.Gln | AcCoA.GlnM1 > AcCoA.Gln | AcCoA.GlnM1 > AcCoA.Gln |
| AcCoA.GlnM2 > AcCoA.Gln | AcCoA.GlnM2 > AcCoA.Gln | AcCoA.GlnM2 > AcCoA.Gln |
| AcCoA.Leu > AcCoA | AcCoA.Leu > AcCoA | AcCoA.Leu > AcCoA |
| AcCoA.LeuM1 > AcCoA.Leu | AcCoA.LeuM1 > AcCoA.Leu | AcCoA.LeuM1 > AcCoA.Leu |
| AcCoA.LeuM2 > AcCoA.Leu | AcCoA.LeuM2 > AcCoA.Leu | AcCoA.LeuM2 > AcCoA.Leu |
| AcCoA.Ile > AcCoA | AcCoA.Ile > AcCoA | AcCoA.Ile > AcCoA |
| AcCoA.IleM1 > AcCoA.Ile | AcCoA.IleM1 > AcCoA.Ile | AcCoA.IleM1 > AcCoA.Ile |
| AcCoA.IleM2 > AcCoA.Ile | AcCoA.IleM2 > AcCoA.Ile | AcCoA.IleM2 > AcCoA.Ile |
| 7 AcCoA > C14 | 8 AcCoA > C16 | 9 AcCoA > C18 |

**Table AT.** ISA models for odd chain fatty acids.

| *C15:0 model* | *C17:0 model* |
| --- | --- |
| AcCoA.Gln > AcCoA | AcCoA.Gln > AcCoA |
| AcCoA.GlnM1 > AcCoA.Gln | AcCoA.GlnM1 > AcCoA.Gln |
| AcCoA.GlnM2 > AcCoA.Gln | AcCoA.GlnM2 > AcCoA.Gln |
| AcCoA.Leu > AcCoA | AcCoA.Leu > AcCoA |
| AcCoA.LeuM1 > AcCoA.Leu | AcCoA.LeuM1 > AcCoA.Leu |
| AcCoA.LeuM2 > AcCoA.Leu | AcCoA.LeuM2 > AcCoA.Leu |
| AcCoA.Ile > AcCoA | AcCoA.Ile > AcCoA |
| AcCoA.IleM1 > AcCoA.Ile | AcCoA.IleM1 > AcCoA.Ile |
| AcCoA.IleM2 > AcCoA.Ile | AcCoA.IleM2 > AcCoA.Ile |
| PropCoA.Val > PropCoA | PropCoA.Val > PropCoA |
| PropCoA.ValM1 > PropCoA.Val | PropCoA.ValM1 > PropCoA.Val |
| PropCoA.ValM2 > PropCoA.Val | PropCoA.ValM2 > PropCoA.Val |
| PropCoA.ValM3 > PropCoA.Val | PropCoA.ValM3 > PropCoA.Val |
| PropCoA.Ile > PropCoA | PropCoA.Ile > PropCoA |
| PropCoA.IleM1 > PropCoA.Ile | PropCoA.IleM1 > PropCoA.Ile |
| PropCoA.IleM2 > PropCoA.Ile | PropCoA.IleM2 > PropCoA.Ile |
| PropCoA.IleM3 > PropCoA.Ile | PropCoA.IleM3 > PropCoA.Ile |
| 6 AcCoA + PropCoA > C15 | 7 AcCoA + PropCoA > C17 |

**Table AU.** ISA model fits for myristate (C14:0).

| *Flux* | Expt 1 | Expt 2 | Expt 3 | Expt 4 | Mean ± SEM |
| --- | --- | --- | --- | --- | --- |
| AcCoA.Gln > AcCoA | 10.0 | 10.6 | 9.9 | 9.2 | 9.9 ± 0.3 |
| AcCoA.GlnM1 > AcCoA.Gln | 0.4 | 0.8 | 0.5 | 0.6 | 0.6 ± 0.1 |
| AcCoA.GlnM2 > AcCoA.Gln | 9.5 | 9.8 | 9.3 | 8.6 | 9.3 ± 0.3 |
| AcCoA.Leu > AcCoA | 18.1 | 18.3 | 17.5 | 20.6 | 18.6 ± 0.7 |
| AcCoA.LeuM1 > AcCoA.Leu | 7.1 | 7.4 | 6.8 | 8.3 | 7.4 ± 0.3 |
| AcCoA.LeuM2 > AcCoA.Leu | 11.0 | 10.9 | 10.7 | 12.4 | 11.2 ± 0.4 |
| AcCoA.Ile > AcCoA | 7.9 | 8.1 | 7.6 | 8.1 | 7.9 ± 0.1 |
| AcCoA.IleM1 > AcCoA.Ile | 0.4 | 0.8 | 0.4 | 0.8 | 0.6 ± 0.1 |
| AcCoA.IleM2 > AcCoA.Ile | 7.5 | 7.2 | 7.2 | 7.3 | 7.3 ± 0.1 |
| 7 AcCoA > C14 | 28.5 | 29.4 | 32.0 | 30.4 | 30.1 ± 0.8 |

**Table AV.** Measured vs. simulated mass isotopomer distributions (MIDs) of methyl-myristate (C14:0) fragment at *m/z* 242 (FAME, C1-C14) for parallel labeling experiment #1 (SSR = 3.4, statistically accepted fit) with [U-^13^C]valine, [U-^13^C]leucine, [U-^13^C]isoleucine, and [U-^13^C]glutamine measured on day 7 (t=24h). Data was corrected for natural isotope abundances.

| *m/z* | [U-^13^C]Val | | [U-^13^C]Leu | | [U-^13^C,^15^N]Ile | | [U-^13^C]Gln | |
| --- | --- | --- | --- | --- | --- | --- | --- | --- |
|  | Expt | Sim | Expt | Sim | Expt | Sim | Expt | Sim |
| 242 (M0) | 0.999 | 1.000 | 0.879 | 0.879 | 0.933 | 0.934 | 0.854 | 0.855 |
| 243 (M1) | 0.000 | 0.000 | 0.036 | 0.038 | 0.004 | 0.004 | 0.006 | 0.007 |
| 244 (M2) | 0.001 | 0.000 | 0.058 | 0.060 | 0.055 | 0.055 | 0.099 | 0.098 |
| 245 (M3) | 0.000 | 0.000 | 0.013 | 0.011 | 0.001 | 0.001 | 0.004 | 0.004 |
| 246 (M4) |  |  | 0.010 | 0.009 | 0.006 | 0.006 | 0.027 | 0.030 |
| 247 (M5) |  |  | 0.002 | 0.001 | 0.000 | 0.000 | 0.002 | 0.001 |
| 248 (M6) |  |  | 0.002 | 0.001 | 0.001 | 0.000 | 0.007 | 0.005 |
| 249 (M7) |  |  |  |  |  |  | 0.002 | 0.000 |

**Table AW.** Measured vs. simulated mass isotopomer distributions (MIDs) of methyl-myristate (C14:0) fragment at *m/z* 242 (FAME, C1-C14) for parallel labeling experiment #2 (SSres = 7.8, statistically accepted fit) with [U-^13^C]valine, [U-^13^C]leucine, [U-^13^C]isoleucine, and [U-^13^C]glutamine measured on day 7 (t=24h). Data was corrected for natural isotope abundances.

| *m/z* | [U-^13^C]Val | | [U-^13^C]Leu | | [U-^13^C]Ile | | [U-^13^C]Gln | |
| --- | --- | --- | --- | --- | --- | --- | --- | --- |
|  | Expt | Sim | Expt | Sim | Expt | Sim | Expt | Sim |
| 242 (M0) | 0.998 | 1.000 | 0.874 | 0.874 | 0.930 | 0.930 | 0.842 | 0.844 |
| 243 (M1) | 0.002 | 0.000 | 0.039 | 0.041 | 0.007 | 0.007 | 0.009 | 0.010 |
| 244 (M2) | 0.001 | 0.000 | 0.060 | 0.062 | 0.054 | 0.055 | 0.101 | 0.100 |
| 245 (M3) | 0.000 | 0.000 | 0.014 | 0.012 | 0.001 | 0.002 | 0.004 | 0.007 |
| 246 (M4) |  |  | 0.011 | 0.009 | 0.007 | 0.006 | 0.028 | 0.031 |
| 247 (M5) |  |  | 0.002 | 0.002 | 0.000 | 0.000 | 0.005 | 0.002 |
| 248 (M6) |  |  | 0.001 | 0.001 | 0.000 | 0.000 | 0.008 | 0.005 |
| 249 (M7) |  |  |  |  |  |  | 0.004 | 0.000 |

**Table AX.** Measured vs. simulated mass isotopomer distributions (MIDs) of methyl-myristate (C14:0) fragment at *m/z* 242 (FAME, C1-C14) for parallel labeling experiment #3 (SSR = 2.8, statistically accepted fit) with [U-^13^C]valine, [U-^13^C]leucine, [U-^13^C]isoleucine, and [U-^13^C]glutamine measured on day 7 (t=24h). Data was corrected for natural isotope abundances.

| *m/z* | [U-^13^C]Val | | [U-^13^C]Leu | | [U-^13^C]Ile | | [U-^13^C]Gln | |
| --- | --- | --- | --- | --- | --- | --- | --- | --- |
|  | Expt | Sim | Expt | Sim | Expt | Sim | Expt | Sim |
| 242 (M0) | 0.998 | 1.000 | 0.868 | 0.868 | 0.928 | 0.928 | 0.838 | 0.838 |
| 243 (M1) | 0.000 | 0.000 | 0.039 | 0.041 | 0.005 | 0.005 | 0.008 | 0.009 |
| 244 (M2) | 0.002 | 0.000 | 0.065 | 0.067 | 0.059 | 0.060 | 0.110 | 0.109 |
| 245 (M3) | 0.000 | 0.000 | 0.013 | 0.012 | 0.001 | 0.001 | 0.005 | 0.005 |
| 246 (M4) |  |  | 0.011 | 0.010 | 0.007 | 0.006 | 0.030 | 0.032 |
| 247 (M5) |  |  | 0.003 | 0.001 | 0.000 | 0.000 | 0.002 | 0.001 |
| 248 (M6) |  |  | 0.001 | 0.001 | 0.001 | 0.000 | 0.008 | 0.005 |
| 249 (M7) |  |  |  |  |  |  | 0.000 | 0.000 |

**Table AY.** Measured vs. simulated mass isotopomer distributions (MIDs) of methyl-myristate (C14:0) fragment at *m/z* 242 (FAME, C1-C14) for parallel labeling experiment #4 (SSR = 1.2, statistically accepted fit) with [U-^13^C]valine, [U-^13^C]leucine, [U-^13^C]isoleucine, and [U-^13^C]glutamine measured on day 7 (t=24h). Data was corrected for natural isotope abundances.

| *m/z* | [U-^13^C]Val | | [U-^13^C]Leu | | [U-^13^C]Ile | | [U-^13^C]Gln | |
| --- | --- | --- | --- | --- | --- | --- | --- | --- |
|  | Expt | Sim | Expt | Sim | Expt | Sim | Expt | Sim |
| 242 (M0) | 0.999 | 1.000 | 0.857 | 0.858 | 0.927 | 0.927 | 0.854 | 0.854 |
| 243 (M1) | 0.000 | 0.000 | 0.043 | 0.044 | 0.008 | 0.008 | 0.010 | 0.009 |
| 244 (M2) | 0.001 | 0.000 | 0.067 | 0.068 | 0.057 | 0.057 | 0.101 | 0.100 |
| 245 (M3) | 0.000 | 0.000 | 0.016 | 0.015 | 0.001 | 0.002 | 0.004 | 0.005 |
| 246 (M4) |  |  | 0.012 | 0.012 | 0.007 | 0.006 | 0.026 | 0.027 |
| 247 (M5) |  |  | 0.003 | 0.002 | 0.000 | 0.000 | 0.001 | 0.001 |
| 248 (M6) |  |  | 0.002 | 0.001 | 0.001 | 0.000 | 0.005 | 0.004 |
| 249 (M7) |  |  |  |  |  |  | 0.000 | 0.000 |

**Table AZ.** ISA model fits for pentadecanoate (C15:0).

| *Flux* | Expt 1 | Expt 2 | Expt 3 | Expt 4 | Mean ± SEM |
| --- | --- | --- | --- | --- | --- |
| AcCoA.Gln > AcCoA | 7.9 | 8.3 | 7.9 | 7.4 | 7.9 ± 0.2 |
| AcCoA.GlnM1 > AcCoA.Gln | 0.5 | 0.8 | 0.4 | 0.6 | 0.6 ± 0.1 |
| AcCoA.GlnM2 > AcCoA.Gln | 7.3 | 7.5 | 7.5 | 6.8 | 7.3 ± 0.2 |
| AcCoA.Leu > AcCoA | 18.3 | 18.1 | 16.3 | 18.9 | 17.9 ± 0.6 |
| AcCoA.LeuM1 > AcCoA.Leu | 7.0 | 7.5 | 6.0 | 7.6 | 7.0 ± 0.4 |
| AcCoA.LeuM2 > AcCoA.Leu | 11.3 | 10.5 | 10.3 | 11.3 | 10.8 ± 0.3 |
| AcCoA.Ile > AcCoA | 8.2 | 8.6 | 7.8 | 8.4 | 8.2 ± 0.2 |
| AcCoA.IleM1 > AcCoA.Ile | 0.5 | 0.8 | 0.2 | 1.0 | 0.6 ± 0.2 |
| AcCoA.IleM2 > AcCoA.Ile | 7.6 | 7.8 | 7.6 | 7.4 | 7.6 ± 0.1 |
| PropCoA.Val > PropCoA | 32.3 | 33.4 | 32.5 | 31.6 | 32.5 ± 0.4 |
| PropCoA.ValM1 > PropCoA.Val | 0.0 | 1.2 | 0.0 | 0.0 | 0.3 ± 0.3 |
| PropCoA.ValM2 > PropCoA.Val | 4.1 | 4.4 | 4.1 | 4.1 | 4.2 ± 0.1 |
| PropCoA.ValM3 > PropCoA.Val | 28.2 | 27.8 | 28.4 | 27.5 | 28.0 ± 0.2 |
| PropCoA.Ile > PropCoA | 67.7 | 66.6 | 67.5 | 68.4 | 67.5 ± 0.4 |
| PropCoA.IleM1 > PropCoA.Ile | 0.0 | 0.0 | 0.0 | 0.0 | 0.0 ± 0.0 |
| PropCoA.IleM2 > PropCoA.Ile | 3.4 | 2.2 | 3.4 | 4.4 | 3.3 ± 0.5 |
| PropCoA.IleM3 > PropCoA.Ile | 64.3 | 64.5 | 64.1 | 64.0 | 64.2 ± 0.1 |
| 6 AcCoA + PropCoA > C15 | 55.9 | 56.0 | 57.7 | 57.1 | 56.7 ± 0.4 |

**Table BA.** Measured vs. simulated mass isotopomer distributions (MIDs) of methyl-pentadecanoate (C15:0) fragment at *m/z* 256 (FAME, C1-C15) for parallel labeling experiment #1 (SSR = 34.2, statistically accepted fit) with [U-^13^C]valine, [U-^13^C]leucine, [U-^13^C]isoleucine, and [U-^13^C]glutamine measured on day 7 (t=24h). Data was corrected for natural isotope abundances.

| *m/z* | [U-^13^C]Val | | [U-^13^C]Leu | | [U-^13^C]Ile | | [U-^13^C]Gln | |
| --- | --- | --- | --- | --- | --- | --- | --- | --- |
|  | Expt | Sim | Expt | Sim | Expt | Sim | Expt | Sim |
| 256 (M0) | 0.917 | 0.919 | 0.786 | 0.788 | 0.741 | 0.743 | 0.786 | 0.788 |
| 257 (M1) | 0.000 | 0.000 | 0.062 | 0.068 | 0.005 | 0.006 | 0.009 | 0.015 |
| 258 (M2) | 0.013 | 0.012 | 0.105 | 0.110 | 0.079 | 0.077 | 0.151 | 0.157 |
| 259 (M3) | 0.070 | 0.069 | 0.022 | 0.017 | 0.134 | 0.131 | 0.006 | 0.006 |
| 260 (M4) |  |  | 0.019 | 0.014 | 0.012 | 0.011 | 0.038 | 0.030 |
| 261 (M5) |  |  | 0.004 | 0.002 | 0.024 | 0.029 | 0.001 | 0.001 |
| 262 (M6) |  |  | 0.003 | 0.001 | 0.001 | 0.001 | 0.008 | 0.003 |
| 263 (M7) |  |  |  |  | 0.003 | 0.003 | 0.000 | 0.000 |

**Table BB.** Measured vs. simulated mass isotopomer distributions (MIDs) of methyl-pentadecanoate (C15:0) fragment at *m/z* 256 (FAME, C1-C15) for parallel labeling experiment #2 (SSR = 50.1, statistically accepted fit) with [U-^13^C]valine, [U-^13^C]leucine, [U-^13^C]isoleucine, and [U-^13^C]glutamine measured on day 7 (t=24h). Data was corrected for natural isotope abundances.

| *m/z* | [U-^13^C]Val | | [U-^13^C]Leu | | [U-^13^C]Ile | | [U-^13^C]Gln | |
| --- | --- | --- | --- | --- | --- | --- | --- | --- |
|  | Expt | Sim | Expt | Sim | Expt | Sim | Expt | Sim |
| 256 (M0) | 0.912 | 0.916 | 0.787 | 0.790 | 0.738 | 0.740 | 0.776 | 0.778 |
| 257 (M1) | 0.005 | 0.003 | 0.067 | 0.073 | 0.008 | 0.009 | 0.014 | 0.020 |
| 258 (M2) | 0.014 | 0.013 | 0.100 | 0.105 | 0.079 | 0.076 | 0.149 | 0.158 |
| 259 (M3) | 0.069 | 0.068 | 0.022 | 0.017 | 0.135 | 0.130 | 0.007 | 0.008 |
| 260 (M4) |  |  | 0.017 | 0.013 | 0.013 | 0.012 | 0.043 | 0.031 |
| 261 (M5) |  |  | 0.004 | 0.002 | 0.024 | 0.029 | 0.003 | 0.001 |
| 262 (M6) |  |  | 0.003 | 0.001 | 0.002 | 0.001 | 0.009 | 0.003 |
| 263 (M7) |  |  |  |  | 0.003 | 0.003 | 0.000 | 0.000 |

**Table BC.** Measured vs. simulated mass isotopomer distributions (MIDs) of methyl-pentadecanoate (C15:0) fragment at *m/z* 256 (FAME, C1-C15) for parallel labeling experiment #3 (SSR = 32.8, statistically accepted fit) with [U-^13^C]valine, [U-^13^C]leucine, [U-^13^C]isoleucine, and [U-^13^C]glutamine measured on day 7 (t=24h). Data was corrected for natural isotope abundances.

| *m/z* | [U-^13^C]Val | | [U-^13^C]Leu | | [U-^13^C]Ile | | [U-^13^C]Gln | |
| --- | --- | --- | --- | --- | --- | --- | --- | --- |
|  | Expt | Sim | Expt | Sim | Expt | Sim | Expt | Sim |
| 256 (M0) | 0.913 | 0.916 | 0.798 | 0.800 | 0.736 | 0.738 | 0.778 | 0.780 |
| 257 (M1) | 0.000 | 0.000 | 0.057 | 0.063 | 0.001 | 0.003 | 0.007 | 0.013 |
| 258 (M2) | 0.014 | 0.013 | 0.103 | 0.108 | 0.082 | 0.080 | 0.159 | 0.166 |
| 259 (M3) | 0.073 | 0.072 | 0.021 | 0.015 | 0.139 | 0.135 | 0.006 | 0.005 |
| 260 (M4) |  |  | 0.016 | 0.013 | 0.013 | 0.010 | 0.039 | 0.032 |
| 261 (M5) |  |  | 0.004 | 0.001 | 0.025 | 0.029 | 0.001 | 0.001 |
| 262 (M6) |  |  | 0.002 | 0.001 | 0.001 | 0.001 | 0.010 | 0.003 |
| 263 (M7) |  |  |  |  | 0.003 | 0.003 | 0.000 | 0.000 |

**Table BD.** Measured vs. simulated mass isotopomer distributions (MIDs) of methyl-pentadecanoate (C15:0) fragment at *m/z* 256 (FAME, C1-C15) for parallel labeling experiment #4 (SSR = 25.0, statistically accepted fit) with [U-^13^C]valine, [U-^13^C]leucine, [U-^13^C]isoleucine, and [U-^13^C]glutamine measured on day 7 (t=24h). Data was corrected for natural isotope abundances.

| *m/z* | [U-^13^C]Val | | [U-^13^C]Leu | | [U-^13^C]Ile | | [U-^13^C]Gln | |
| --- | --- | --- | --- | --- | --- | --- | --- | --- |
|  | Expt | Sim | Expt | Sim | Expt | Sim | Expt | Sim |
| 256 (M0) | 0.916 | 0.919 | 0.775 | 0.778 | 0.732 | 0.734 | 0.792 | 0.793 |
| 257 (M1) | 0.001 | 0.000 | 0.068 | 0.074 | 0.010 | 0.011 | 0.014 | 0.017 |
| 258 (M2) | 0.013 | 0.012 | 0.107 | 0.112 | 0.080 | 0.078 | 0.148 | 0.153 |
| 259 (M3) | 0.069 | 0.069 | 0.024 | 0.019 | 0.136 | 0.133 | 0.005 | 0.006 |
| 260 (M4) |  |  | 0.019 | 0.015 | 0.013 | 0.013 | 0.034 | 0.027 |
| 261 (M5) |  |  | 0.004 | 0.002 | 0.025 | 0.028 | 0.001 | 0.001 |
| 262 (M6) |  |  | 0.003 | 0.001 | 0.001 | 0.001 | 0.007 | 0.003 |
| 263 (M7) |  |  |  |  | 0.003 | 0.003 | 0.000 | 0.000 |

**Table BE.** ISA model fits for palmitate (C16:0).

| *Flux* | Expt 1 | Expt 2 | Expt 3 | Expt 4 | Mean ± SEM |
| --- | --- | --- | --- | --- | --- |
| AcCoA.Gln > AcCoA | 9.8 | 9.6 | 9.9 | 9.1 | 9.6 ± 0.2 |
| AcCoA.GlnM1 > AcCoA.Gln | 0.4 | 0.5 | 0.5 | 0.6 | 0.5 ± 0.0 |
| AcCoA.GlnM2 > AcCoA.Gln | 9.4 | 9.1 | 9.4 | 8.6 | 9.1 ± 0.2 |
| AcCoA.Leu > AcCoA | 18.3 | 18.5 | 17.7 | 21.0 | 18.9 ± 0.7 |
| AcCoA.LeuM1 > AcCoA.Leu | 7.0 | 7.5 | 6.6 | 8.5 | 7.4 ± 0.4 |
| AcCoA.LeuM2 > AcCoA.Leu | 11.3 | 11.0 | 11.2 | 12.5 | 11.5 ± 0.3 |
| AcCoA.Ile > AcCoA | 7.5 | 7.4 | 7.2 | 8.2 | 7.6 ± 0.2 |
| AcCoA.IleM1 > AcCoA.Ile | 0.5 | 0.6 | 0.1 | 0.9 | 0.5 ± 0.1 |
| AcCoA.IleM2 > AcCoA.Ile | 7.0 | 6.8 | 7.1 | 7.3 | 7.0 ± 0.1 |
| 8 AcCoA > C16 | 32.5 | 33.0 | 34.3 | 33.8 | 33.4 ± 0.4 |

**Table BF.** Measured vs. simulated mass isotopomer distributions (MIDs) of methyl-palmitate (C16:0) fragment at *m/z* 270 (FAME, C1-C16) for parallel labeling experiment #1 (SSR = 7.1, statistically accepted fit) with [U-^13^C]valine, [U-^13^C]leucine, [U-^13^C]isoleucine, and [U-^13^C]glutamine measured on day 7 (t=24h). Data was corrected for natural isotope abundances.

| *m/z* | [U-^13^C]Val | | [U-^13^C]Leu | | [U-^13^C]Ile | | [U-^13^C]Gln | |
| --- | --- | --- | --- | --- | --- | --- | --- | --- |
|  | Expt | Sim | Expt | Sim | Expt | Sim | Expt | Sim |
| 270 (M0) | 0.999 | 1.000 | 0.847 | 0.847 | 0.918 | 0.919 | 0.820 | 0.821 |
| 271 (M1) | 0.000 | 0.000 | 0.041 | 0.045 | 0.005 | 0.006 | 0.007 | 0.007 |
| 272 (M2) | 0.001 | 0.000 | 0.071 | 0.074 | 0.065 | 0.066 | 0.118 | 0.116 |
| 273 (M3) | 0.000 | 0.000 | 0.019 | 0.016 | 0.002 | 0.001 | 0.006 | 0.005 |
| 274 (M4) |  |  | 0.016 | 0.014 | 0.009 | 0.008 | 0.037 | 0.040 |
| 275 (M5) |  |  | 0.004 | 0.003 | 0.000 | 0.000 | 0.001 | 0.002 |
| 276 (M6) |  |  | 0.003 | 0.001 | 0.001 | 0.001 | 0.011 | 0.008 |
| 277 (M7) |  |  |  |  |  |  | 0.001 | 0.000 |

**Table FG.** Measured vs. simulated mass isotopomer distributions (MIDs) of methyl-palmitate (C16:0) fragment at *m/z* 270 (FAME, C1-C16) for parallel labeling experiment #2 (SSR = 5.5, statistically accepted fit) with [U-^13^C]valine, [U-^13^C]leucine, [U-^13^C]isoleucine, and [U-^13^C]glutamine measured on day 7 (t=24h). Data was corrected for natural isotope abundances.

| *m/z* | [U-^13^C]Val | | [U-^13^C]Leu | | [U-^13^C]Ile | | [U-^13^C]Gln | |
| --- | --- | --- | --- | --- | --- | --- | --- | --- |
|  | Expt | Sim | Expt | Sim | Expt | Sim | Expt | Sim |
| 270 (M0) | 0.998 | 1.000 | 0.843 | 0.843 | 0.919 | 0.919 | 0.821 | 0.822 |
| 271 (M1) | 0.001 | 0.000 | 0.046 | 0.048 | 0.006 | 0.007 | 0.009 | 0.009 |
| 272 (M2) | 0.001 | 0.000 | 0.071 | 0.074 | 0.064 | 0.065 | 0.117 | 0.116 |
| 273 (M3) | 0.000 | 0.000 | 0.018 | 0.017 | 0.002 | 0.002 | 0.006 | 0.006 |
| 274 (M4) |  |  | 0.015 | 0.013 | 0.009 | 0.007 | 0.036 | 0.039 |
| 275 (M5) |  |  | 0.004 | 0.003 | 0.000 | 0.000 | 0.002 | 0.002 |
| 276 (M6) |  |  | 0.003 | 0.001 | 0.001 | 0.001 | 0.010 | 0.007 |
| 277 (M7) |  |  |  |  |  |  | 0.001 | 0.000 |

**Table BH.** Measured vs. simulated mass isotopomer distributions (MIDs) of methyl-palmitate (C16:0) fragment at *m/z* 270 (FAME, C1-C16) for parallel labeling experiment #3 (SSR = 7.8, statistically accepted fit) with [U-^13^C]valine, [U-^13^C]leucine, [U-^13^C]isoleucine, and [U-^13^C]glutamine measured on day 7 (t=24h). Data was corrected for natural isotope abundances.

| *m/z* | [U-^13^C]Val | | [U-^13^C]Leu | | [U-^13^C]Ile | | [U-^13^C]Gln | |
| --- | --- | --- | --- | --- | --- | --- | --- | --- |
|  | Expt | Sim | Expt | Sim | Expt | Sim | Expt | Sim |
| 270 (M0) | 0.998 | 1.000 | 0.842 | 0.842 | 0.917 | 0.917 | 0.809 | 0.810 |
| 271 (M1) | 0.000 | 0.000 | 0.041 | 0.045 | 0.001 | 0.003 | 0.007 | 0.009 |
| 272 (M2) | 0.002 | 0.000 | 0.076 | 0.078 | 0.070 | 0.070 | 0.123 | 0.122 |
| 273 (M3) | 0.000 | 0.000 | 0.019 | 0.016 | 0.002 | 0.001 | 0.007 | 0.006 |
| 274 (M4) |  |  | 0.015 | 0.014 | 0.009 | 0.008 | 0.039 | 0.042 |
| 275 (M5) |  |  | 0.004 | 0.002 | 0.000 | 0.000 | 0.002 | 0.002 |
| 276 (M6) |  |  | 0.003 | 0.002 | 0.001 | 0.001 | 0.012 | 0.008 |
| 277 (M7) |  |  |  |  |  |  | 0.001 | 0.000 |

**Table BI.** Measured vs. simulated mass isotopomer distributions (MIDs) of methyl-palmitate (C16:0) fragment at *m/z* 270 (FAME, C1-C16) for parallel labeling experiment #4 (SSR = 2.8, statistically accepted fit) with [U-^13^C]valine, [U-^13^C]leucine, [U-^13^C]isoleucine, and [U-^13^C]glutamine measured on day 7 (t=24h). Data was corrected for natural isotope abundances.

| *m/z* | [U-^13^C]Val | | [U-^13^C]Leu | | [U-^13^C]Ile | | [U-^13^C]Gln | |
| --- | --- | --- | --- | --- | --- | --- | --- | --- |
|  | Expt | Sim | Expt | Sim | Expt | Sim | Expt | Sim |
| 270 (M0) | 0.999 | 1.000 | 0.824 | 0.824 | 0.909 | 0.909 | 0.823 | 0.823 |
| 271 (M1) | 0.000 | 0.000 | 0.050 | 0.052 | 0.010 | 0.010 | 0.011 | 0.010 |
| 272 (M2) | 0.001 | 0.000 | 0.079 | 0.080 | 0.069 | 0.069 | 0.117 | 0.115 |
| 273 (M3) | 0.000 | 0.000 | 0.021 | 0.021 | 0.002 | 0.002 | 0.006 | 0.006 |
| 274 (M4) |  |  | 0.017 | 0.017 | 0.009 | 0.009 | 0.034 | 0.036 |
| 275 (M5) |  |  | 0.005 | 0.004 | 0.000 | 0.000 | 0.001 | 0.002 |
| 276 (M6) |  |  | 0.003 | 0.002 | 0.001 | 0.001 | 0.009 | 0.006 |
| 277 (M7) |  |  |  |  |  |  | 0.000 | 0.000 |

**Table BJ.** ISA model fits for heptadecanoate (C17:0).

| *Flux* | Expt 1 | Expt 2 | Expt 3 | Expt 4 | Mean ± SEM |
| --- | --- | --- | --- | --- | --- |
| AcCoA.Gln > AcCoA | 8.3 | 8.1 | 8.3 | 7.8 | 8.1 ± 0.1 |
| AcCoA.GlnM1 > AcCoA.Gln | 0.6 | 0.6 | 0.7 | 0.7 | 0.7 ± 0.0 |
| AcCoA.GlnM2 > AcCoA.Gln | 7.7 | 7.4 | 7.6 | 7.1 | 7.5 ± 0.1 |
| AcCoA.Leu > AcCoA | 18.0 | 17.6 | 17.5 | 18.9 | 18.0 ± 0.3 |
| AcCoA.LeuM1 > AcCoA.Leu | 6.9 | 7.1 | 6.9 | 7.5 | 7.1 ± 0.2 |
| AcCoA.LeuM2 > AcCoA.Leu | 11.1 | 10.6 | 10.7 | 11.3 | 10.9 ± 0.2 |
| AcCoA.Ile > AcCoA | 8.1 | 7.8 | 8.3 | 8.1 | 8.1 ± 0.1 |
| AcCoA.IleM1 > AcCoA.Ile | 0.5 | 0.7 | 0.7 | 0.9 | 0.7 ± 0.1 |
| AcCoA.IleM2 > AcCoA.Ile | 7.6 | 7.1 | 7.6 | 7.2 | 7.4 ± 0.1 |
| PropCoA.Val > PropCoA | 32.7 | 32.3 | 33.2 | 31.9 | 32.5 ± 0.3 |
| PropCoA.ValM1 > PropCoA.Val | 0.0 | 0.0 | 0.0 | 0.1 | 0.0 ± 0.0 |
| PropCoA.ValM2 > PropCoA.Val | 4.2 | 4.5 | 4.3 | 4.3 | 4.3 ± 0.1 |
| PropCoA.ValM3 > PropCoA.Val | 28.4 | 27.8 | 28.9 | 27.6 | 28.2 ± 0.3 |
| PropCoA.Ile > PropCoA | 67.3 | 67.7 | 66.8 | 68.1 | 67.5 ± 0.3 |
| PropCoA.IleM1 > PropCoA.Ile | 0.0 | 0.0 | 0.0 | 0.0 | 0.0 ± 0.0 |
| PropCoA.IleM2 > PropCoA.Ile | 2.5 | 3.8 | 2.2 | 4.5 | 3.2 ± 0.5 |
| PropCoA.IleM3 > PropCoA.Ile | 64.9 | 63.9 | 64.6 | 63.6 | 64.2 ± 0.3 |
| 7 AcCoA + PropCoA > C17 | 62.4 | 62.1 | 62.8 | 61.9 | 62.3 ± 0.3 |

**Table BK.** Measured vs. simulated mass isotopomer distributions (MIDs) of methyl-heptadecanoate (C17:0) fragment at *m/z* 284 (FAME, C1-C17) for parallel labeling experiment #1 (SSR = 58.2, statistically accepted fit) with [U-^13^C]valine, [U-^13^C]leucine, [U-^13^C]isoleucine, and [U-^13^C]glutamine measured on day 7 (t=24h). Data was corrected for natural isotope abundances.

| *m/z* | [U-^13^C]Val | | [U-^13^C]Leu | | [U-^13^C]Ile | | [U-^13^C]Gln | |
| --- | --- | --- | --- | --- | --- | --- | --- | --- |
|  | Expt | Sim | Expt | Sim | Expt | Sim | Expt | Sim |
| 284 (M0) | 0.905 | 0.908 | 0.734 | 0.736 | 0.698 | 0.701 | 0.720 | 0.722 |
| 285 (M1) | 0.000 | 0.000 | 0.073 | 0.081 | 0.007 | 0.007 | 0.013 | 0.019 |
| 286 (M2) | 0.016 | 0.014 | 0.126 | 0.133 | 0.097 | 0.093 | 0.185 | 0.194 |
| 287 (M3) | 0.079 | 0.078 | 0.030 | 0.025 | 0.148 | 0.142 | 0.006 | 0.009 |
| 288 (M4) |  |  | 0.025 | 0.021 | 0.015 | 0.015 | 0.053 | 0.047 |
| 289 (M5) |  |  | 0.006 | 0.003 | 0.030 | 0.036 | 0.002 | 0.002 |
| 290 (M6) |  |  | 0.005 | 0.002 | 0.002 | 0.002 | 0.016 | 0.006 |
| 291 (M7) |  |  |  |  | 0.004 | 0.004 | 0.000 | 0.000 |

**Table BL.** Measured vs. simulated mass isotopomer distributions (MIDs) of methyl-heptadecanoate (C17:0) fragment at *m/z* 284 (FAME, C1-C17) for parallel labeling experiment #2 (SSR = 47.7, statistically accepted fit) with [U-^13^C]valine, [U-^13^C]leucine, [U-^13^C]isoleucine, and [U-^13^C]glutamine measured on day 7 (t=24h). Data was corrected for natural isotope abundances.

| *m/z* | [U-^13^C]Val | | [U-^13^C]Leu | | [U-^13^C]Ile | | [U-^13^C]Gln | |
| --- | --- | --- | --- | --- | --- | --- | --- | --- |
|  | Expt | Sim | Expt | Sim | Expt | Sim | Expt | Sim |
| 284 (M0) | 0.906 | 0.910 | 0.740 | 0.742 | 0.703 | 0.705 | 0.727 | 0.729 |
| 285 (M1) | 0.002 | 0.000 | 0.076 | 0.083 | 0.009 | 0.009 | 0.016 | 0.020 |
| 286 (M2) | 0.016 | 0.015 | 0.121 | 0.128 | 0.094 | 0.091 | 0.182 | 0.189 |
| 287 (M3) | 0.077 | 0.075 | 0.029 | 0.024 | 0.147 | 0.142 | 0.006 | 0.009 |
| 288 (M4) |  |  | 0.024 | 0.019 | 0.015 | 0.015 | 0.050 | 0.044 |
| 289 (M5) |  |  | 0.007 | 0.003 | 0.028 | 0.034 | 0.002 | 0.002 |
| 290 (M6) |  |  | 0.004 | 0.002 | 0.001 | 0.002 | 0.013 | 0.006 |
| 291 (M7) |  |  |  |  | 0.004 | 0.003 | 0.001 | 0.000 |

**Table BM.** Measured vs. simulated mass isotopomer distributions (MIDs) of methyl-heptadecanoate (C17:0) fragment at *m/z* 284 (FAME, C1-C17) for parallel labeling experiment #3 (SSR = 67.7, statistically accepted fit) with [U-^13^C]valine, [U-^13^C]leucine, [U-^13^C]isoleucine, and [U-^13^C]glutamine measured on day 7 (t=24h). Data was corrected for natural isotope abundances.

| *m/z* | [U-^13^C]Val | | [U-^13^C]Leu | | [U-^13^C]Ile | | [U-^13^C]Gln | |
| --- | --- | --- | --- | --- | --- | --- | --- | --- |
|  | Expt | Sim | Expt | Sim | Expt | Sim | Expt | Sim |
| 284 (M0) | 0.902 | 0.906 | 0.738 | 0.740 | 0.695 | 0.698 | 0.718 | 0.720 |
| 285 (M1) | 0.000 | 0.000 | 0.075 | 0.082 | 0.010 | 0.010 | 0.016 | 0.022 |
| 286 (M2) | 0.016 | 0.014 | 0.124 | 0.130 | 0.097 | 0.092 | 0.183 | 0.193 |
| 287 (M3) | 0.081 | 0.079 | 0.028 | 0.024 | 0.149 | 0.143 | 0.006 | 0.010 |
| 288 (M4) |  |  | 0.025 | 0.019 | 0.015 | 0.016 | 0.055 | 0.046 |
| 289 (M5) |  |  | 0.007 | 0.003 | 0.029 | 0.036 | 0.002 | 0.002 |
| 290 (M6) |  |  | 0.004 | 0.002 | 0.002 | 0.002 | 0.015 | 0.006 |
| 291 (M7) |  |  |  |  | 0.004 | 0.004 | 0.001 | 0.000 |

**Table BN.** Measured vs. simulated mass isotopomer distributions (MIDs) of methyl-heptadecanoate (C17:0) fragment at *m/z* 284 (FAME, C1-C17) for parallel labeling experiment #4 (SSR = 47.5, statistically accepted fit) with [U-^13^C]valine, [U-^13^C]leucine, [U-^13^C]isoleucine, and [U-^13^C]glutamine measured on day 7 (t=24h). Data was corrected for natural isotope abundances.

| *m/z* | [U-^13^C]Val | | [U-^13^C]Leu | | [U-^13^C]Ile | | [U-^13^C]Gln | |
| --- | --- | --- | --- | --- | --- | --- | --- | --- |
|  | Expt | Sim | Expt | Sim | Expt | Sim | Expt | Sim |
| 284 (M0) | 0.907 | 0.911 | 0.727 | 0.729 | 0.699 | 0.701 | 0.736 | 0.738 |
| 285 (M1) | 0.002 | 0.000 | 0.078 | 0.086 | 0.013 | 0.012 | 0.018 | 0.021 |
| 286 (M2) | 0.015 | 0.014 | 0.126 | 0.133 | 0.096 | 0.092 | 0.177 | 0.184 |
| 287 (M3) | 0.076 | 0.075 | 0.031 | 0.027 | 0.145 | 0.140 | 0.006 | 0.009 |
| 288 (M4) |  |  | 0.027 | 0.021 | 0.015 | 0.016 | 0.047 | 0.041 |
| 289 (M5) |  |  | 0.007 | 0.003 | 0.028 | 0.034 | 0.001 | 0.002 |
| 290 (M6) |  |  | 0.005 | 0.002 | 0.002 | 0.002 | 0.012 | 0.005 |
| 291 (M7) |  |  |  |  | 0.003 | 0.004 | 0.001 | 0.000 |

**Table BO.** ISA model fits for stearate (C18:0).

| *Flux* | Expt 1 | Expt 2 | Expt 3 | Expt 4 | Mean ± SEM |
| --- | --- | --- | --- | --- | --- |
| AcCoA.Gln > AcCoA | 11.5 | 10.8 | 10.3 | 10.4 | 10.8 ± 0.3 |
| AcCoA.GlnM1 > AcCoA.Gln | 0.9 | 0.2 | 0.1 | 0.4 | 0.4 ± 0.2 |
| AcCoA.GlnM2 > AcCoA.Gln | 10.6 | 10.6 | 10.2 | 10.0 | 10.3 ± 0.1 |
| AcCoA.Leu > AcCoA | 16.6 | 19.2 | 19.3 | 19.1 | 18.6 ± 0.7 |
| AcCoA.LeuM1 > AcCoA.Leu | 6.2 | 6.7 | 7.6 | 7.8 | 7.1 ± 0.4 |
| AcCoA.LeuM2 > AcCoA.Leu | 10.4 | 12.5 | 11.7 | 11.3 | 11.5 ± 0.4 |
| AcCoA.Ile > AcCoA | 6.3 | 6.5 | 6.2 | 7.0 | 6.5 ± 0.2 |
| AcCoA.IleM1 > AcCoA.Ile | 0.6 | 0.0 | 0.1 | 0.6 | 0.3 ± 0.2 |
| AcCoA.IleM2 > AcCoA.Ile | 5.7 | 6.5 | 6.2 | 6.4 | 6.2 ± 0.2 |
| 9 AcCoA > C18 | 17.6 | 16.4 | 18.1 | 17.5 | 17.4 ± 0.4 |

**Table BP.** Measured vs. simulated mass isotopomer distributions (MIDs) of methyl-stearate (C18:0) fragment at *m/z* 298 (FAME, C1-C18) for parallel labeling experiment #1 (SSR = 13.5, statistically accepted fit) with [U-^13^C]valine, [U-^13^C]leucine, [U-^13^C]isoleucine, and [U-^13^C]glutamine measured on day 7 (t=24h). Data was corrected for natural isotope abundances.

| *m/z* | [U-^13^C]Val | | [U-^13^C]Leu | | [U-^13^C]Ile | | [U-^13^C]Gln | |
| --- | --- | --- | --- | --- | --- | --- | --- | --- |
|  | Expt | Sim | Expt | Sim | Expt | Sim | Expt | Sim |
| 298 (M0) | 0.998 | 1.000 | 0.916 | 0.916 | 0.958 | 0.958 | 0.882 | 0.885 |
| 299 (M1) | 0.000 | 0.000 | 0.020 | 0.024 | 0.004 | 0.004 | 0.009 | 0.007 |
| 300 (M2) | 0.002 | 0.000 | 0.038 | 0.041 | 0.033 | 0.033 | 0.063 | 0.062 |
| 301 (M3) | 0.000 | 0.000 | 0.011 | 0.009 | 0.000 | 0.001 | 0.002 | 0.006 |
| 302 (M4) |  |  | 0.009 | 0.008 | 0.004 | 0.004 | 0.024 | 0.028 |
| 303 (M5) |  |  | 0.004 | 0.002 | 0.000 | 0.000 | 0.001 | 0.002 |
| 304 (M6) |  |  | 0.002 | 0.001 | 0.001 | 0.000 | 0.010 | 0.008 |
| 305 (M7) |  |  |  |  | 0.000 | 0.000 | 0.001 | 0.000 |

**Table BQ.** Measured vs. simulated mass isotopomer distributions (MIDs) of methyl-stearate (C18:0) fragment at *m/z* 298 (FAME, C1-C18) for parallel labeling experiment #2 (SSR = 6.0, statistically accepted fit) with [U-^13^C]valine, [U-^13^C]leucine, [U-^13^C]isoleucine, and [U-^13^C]glutamine measured on day 7 (t=24h). Data was corrected for natural isotope abundances.

| *m/z* | [U-^13^C]Val | | [U-^13^C]Leu | | [U-^13^C]Ile | | [U-^13^C]Gln | |
| --- | --- | --- | --- | --- | --- | --- | --- | --- |
|  | Expt | Sim | Expt | Sim | Expt | Sim | Expt | Sim |
| 298 (M0) | 1.000 | 1.000 | 0.913 | 0.913 | 0.960 | 0.960 | 0.895 | 0.897 |
| 299 (M1) | 0.000 | 0.000 | 0.020 | 0.022 | 0.000 | 0.001 | 0.002 | 0.003 |
| 300 (M2) | 0.000 | 0.000 | 0.041 | 0.042 | 0.034 | 0.035 | 0.062 | 0.061 |
| 301 (M3) | 0.000 | 0.000 | 0.011 | 0.010 | 0.001 | 0.000 | 0.001 | 0.002 |
| 302 (M4) |  |  | 0.010 | 0.010 | 0.006 | 0.004 | 0.024 | 0.027 |
| 303 (M5) |  |  | 0.003 | 0.002 | 0.000 | 0.000 | 0.000 | 0.001 |
| 304 (M6) |  |  | 0.002 | 0.001 | 0.000 | 0.000 | 0.009 | 0.007 |
| 305 (M7) |  |  |  |  | 0.000 | 0.000 | 0.001 | 0.000 |

**Table BR.** Measured vs. simulated mass isotopomer distributions (MIDs) of methyl-stearate (C18:0) fragment at *m/z* 298 (FAME, C1-C18) for parallel labeling experiment #3 (SSR = 6.6, statistically accepted fit) with [U-^13^C]valine, [U-^13^C]leucine, [U-^13^C]isoleucine, and [U-^13^C]glutamine measured on day 7 (t=24h). Data was corrected for natural isotope abundances.

| *m/z* | [U-^13^C]Val | | [U-^13^C]Leu | | [U-^13^C]Ile | | [U-^13^C]Gln | |
| --- | --- | --- | --- | --- | --- | --- | --- | --- |
|  | Expt | Sim | Expt | Sim | Expt | Sim | Expt | Sim |
| 298 (M0) | 0.997 | 1.000 | 0.904 | 0.904 | 0.957 | 0.958 | 0.888 | 0.889 |
| 299 (M1) | 0.000 | 0.000 | 0.027 | 0.027 | 0.001 | 0.001 | 0.000 | 0.002 |
| 300 (M2) | 0.003 | 0.000 | 0.043 | 0.044 | 0.036 | 0.037 | 0.069 | 0.068 |
| 301 (M3) | 0.000 | 0.000 | 0.011 | 0.012 | 0.000 | 0.000 | 0.001 | 0.002 |
| 302 (M4) |  |  | 0.011 | 0.010 | 0.005 | 0.004 | 0.026 | 0.029 |
| 303 (M5) |  |  | 0.004 | 0.002 | 0.000 | 0.000 | 0.001 | 0.001 |
| 304 (M6) |  |  | 0.001 | 0.001 | 0.000 | 0.000 | 0.010 | 0.007 |
| 305 (M7) |  |  |  |  | 0.000 | 0.000 | 0.000 | 0.000 |

**Table BS.** Measured vs. simulated mass isotopomer distributions (MIDs) of methyl-stearate (C18:0) fragment at *m/z* 298 (FAME, C1-C18) for parallel labeling experiment #4 (SSR = 5.8, statistically accepted fit) with [U-^13^C]valine, [U-^13^C]leucine, [U-^13^C]isoleucine, and [U-^13^C]glutamine measured on day 7 (t=24h). Data was corrected for natural isotope abundances.

| *m/z* | [U-^13^C]Val | | [U-^13^C]Leu | | [U-^13^C]Ile | | [U-^13^C]Gln | |
| --- | --- | --- | --- | --- | --- | --- | --- | --- |
|  | Expt | Sim | Expt | Sim | Expt | Sim | Expt | Sim |
| 298 (M0) | 0.999 | 1.000 | 0.907 | 0.907 | 0.954 | 0.955 | 0.890 | 0.892 |
| 299 (M1) | 0.000 | 0.000 | 0.025 | 0.027 | 0.004 | 0.004 | 0.003 | 0.004 |
| 300 (M2) | 0.000 | 0.000 | 0.040 | 0.042 | 0.035 | 0.036 | 0.065 | 0.064 |
| 301 (M3) | 0.001 | 0.000 | 0.012 | 0.011 | 0.001 | 0.001 | 0.003 | 0.003 |
| 302 (M4) |  |  | 0.010 | 0.009 | 0.005 | 0.004 | 0.023 | 0.027 |
| 303 (M5) |  |  | 0.004 | 0.002 | 0.000 | 0.000 | 0.001 | 0.001 |
| 304 (M6) |  |  | 0.002 | 0.001 | 0.001 | 0.000 | 0.010 | 0.007 |
| 305 (M7) |  |  |  |  |  |  | 0.001 | 0.000 |

**Table BT.** Combined ISA model for all fatty acids.

| *Precursor fluxes* | *Fatty acid synthesis fluxes* |
| --- | --- |
| AcCoA.Gln > AcCoA | 7 AcCoA > C14 |
| AcCoA.GlnM1 > AcCoA.Gln | 6 AcCoA + PropCoA > C15 |
| AcCoA.GlnM2 > AcCoA.Gln | 8 AcCoA > C16 |
| AcCoA.Leu > AcCoA | 7 AcCoA + PropCoA > C17 |
| AcCoA.LeuM1 > AcCoA.Leu | 9 AcCoA > C18 |
| AcCoA.LeuM2 > AcCoA.Leu | 8 AcCoA > C15 + CO_2_ (α-oxidation) |
| AcCoA.Ile > AcCoA | 9 AcCoA > C17 + CO_2_ (α-oxidation) |
| AcCoA.IleM1 > AcCoA.Ile |  |
| AcCoA.IleM2 > AcCoA.Ile |  |
| PropCoA.Val > PropCoA |  |
| PropCoA.ValM1 > PropCoA.Val |  |
| PropCoA.ValM2 > PropCoA.Val |  |
| PropCoA.ValM3 > PropCoA.Val |  |
| PropCoA.Ile > PropCoA |  |
| PropCoA.IleM1 > PropCoA.Ile |  |
| PropCoA.IleM2 > PropCoA.Ile |  |
| PropCoA.IleM3 > PropCoA.Ile |  |
| PropCoA > PropCoA.sink |  |

**Table BU.** Combined ISA model results.

| *Flux* | Expt 1 | Expt 2 | Expt 3 | Expt 4 | Mean ± SEM |
| --- | --- | --- | --- | --- | --- |
| AcCoA.Gln > AcCoA | 8.4 | 8.4 | 8.3 | 7.8 | 8.2 ± 0.1 |
| AcCoA.GlnM1 > AcCoA.Gln | 0.6 | 0.7 | 0.6 | 0.6 | 0.6 ± 0.0 |
| AcCoA.GlnM2 > AcCoA.Gln | 7.8 | 7.7 | 7.7 | 7.2 | 7.6 ± 0.1 |
| AcCoA.Leu > AcCoA | 17.5 | 17.3 | 16.5 | 18.6 | 17.5 ± 0.4 |
| AcCoA.LeuM1 > AcCoA.Leu | 6.7 | 7.0 | 6.3 | 7.5 | 6.9 ± 0.3 |
| AcCoA.LeuM2 > AcCoA.Leu | 10.7 | 10.3 | 10.2 | 11.1 | 10.6 ± 0.2 |
| AcCoA.Ile > AcCoA | 7.3 | 7.2 | 7.0 | 7.5 | 7.3 ± 0.1 |
| AcCoA.IleM1 > AcCoA.Ile | 0.5 | 0.7 | 0.4 | 0.9 | 0.6 ± 0.1 |
| AcCoA.IleM2 > AcCoA.Ile | 6.7 | 6.5 | 6.6 | 6.6 | 6.6 ± 0.0 |
| PropCoA.Val > PropCoA | 31.4 | 31.5 | 31.6 | 30.9 | 31.4 ± 0.2 |
| PropCoA.ValM1 > PropCoA.Val | 0.0 | 0.0 | 0.0 | 0.0 | 0.0 ± 0.0 |
| PropCoA.ValM2 > PropCoA.Val | 3.7 | 4.1 | 3.7 | 3.8 | 3.8 ± 0.1 |
| PropCoA.ValM3 > PropCoA.Val | 27.7 | 27.3 | 27.9 | 27.1 | 27.5 ± 0.2 |
| PropCoA.Ile > PropCoA | 68.6 | 68.5 | 68.4 | 69.1 | 68.7 ± 0.2 |
| PropCoA.IleM1 > PropCoA.Ile | 0.0 | 0.0 | 0.0 | 0.0 | 0.0 ± 0.0 |
| PropCoA.IleM2 > PropCoA.Ile | 6.0 | 6.2 | 6.3 | 6.7 | 6.3 ± 0.1 |
| PropCoA.IleM3 > PropCoA.Ile | 62.6 | 62.3 | 62.1 | 62.4 | 62.4 ± 0.1 |
| 7 AcCoA > C14 | 31.0 | 32.6 | 34.9 | 33.4 | 33.0 ± 0.8 |
| 6 AcCoA + PropCoA > C15 | 56.0 | 56.8 | 57.6 | 57.1 | 56.9 ± 0.3 |
| 8 AcCoA > C16 | 34.8 | 35.3 | 37.3 | 37.0 | 36.1 ± 0.6 |
| 7 AcCoA + PropCoA > C17 | 63.2 | 62.2 | 64.0 | 62.4 | 63.0 ± 0.4 |
| 9 AcCoA > C18 | 18.6 | 18.1 | 20.0 | 19.1 | 19.0 ± 0.4 |
| 8 AcCoA > C15 + CO_2_ (α-oxidation) | 0.00 | 0.00 | 0.00 | 0.00 | 0.0 ± 0.0 |
| 9 AcCoA > C17 + CO_2_ (α-oxidation) | 0.00 | 0.00 | 0.00 | 0.00 | 0.0 ± 0.0 |

**Table BV.** Measured vs. simulated mass isotopomer distributions of fatty acids (FAME derivatives) for parallel labeling experiment #1 (SSR = 218.1, statistically accepted fit) with [U-^13^C]valine, [U-^13^C]leucine, [U-^13^C]isoleucine, and [U-^13^C]glutamine measured on day 7 (t=24h). Combined ISA model used for data fitting. Data was corrected for natural isotope abundances.

| FAME Fragment | M0 | M1 | M2 | M3 | M4 | M5 | M6 | M7 | M8 |
| --- | --- | --- | --- | --- | --- | --- | --- | --- | --- |
| *[U-^13^C]Valine* |  |  |  |  |  |  |  |  |  |
|  |  |  |  |  |  |  |  |  |  |
| C14 (*m/z* 242) |  |  |  |  |  |  |  |  |  |
| Expt | 0.999 | 0.000 | 0.001 | 0.000 | 0.000 | 0.000 |  |  |  |
| Sim | 1.000 | 0.000 | 0.000 | 0.000 | 0.000 | 0.000 |  |  |  |
| C15 (*m/z* 256) |  |  |  |  |  |  |  |  |  |
| Expt | 0.917 | 0.000 | 0.013 | 0.070 | 0.000 | 0.000 |  |  |  |
| Sim | 0.921 | 0.000 | 0.011 | 0.068 | 0.000 | 0.000 |  |  |  |
| C16 (*m/z* 270) |  |  |  |  |  |  |  |  |  |
| Expt | 0.999 | 0.000 | 0.001 | 0.000 | 0.000 | 0.000 |  |  |  |
| Sim | 1.000 | 0.000 | 0.000 | 0.000 | 0.000 | 0.000 |  |  |  |
| C17 (*m/z* 284) |  |  |  |  |  |  |  |  |  |
| Expt | 0.905 | 0.000 | 0.016 | 0.079 | 0.000 | 0.000 |  |  |  |
| Sim | 0.911 | 0.000 | 0.013 | 0.076 | 0.000 | 0.000 |  |  |  |
| C18 (*m/z 298*) |  |  |  |  |  |  |  |  |  |
| Expt | 0.998 | 0.000 | 0.002 | 0.000 | 0.000 | 0.000 |  |  |  |
| Sim | 1.000 | 0.000 | 0.000 | 0.000 | 0.000 | 0.000 |  |  |  |
|  |  |  |  |  |  |  |  |  |  |
| *[U-^13^C]Leucine* |  |  |  |  |  |  |  |  |  |
|  |  |  |  |  |  |  |  |  |  |
| C14 (*m/z* 242) |  |  |  |  |  |  |  |  |  |
| Expt | 0.879 | 0.036 | 0.058 | 0.013 | 0.010 | 0.002 | 0.002 |  |  |
| Sim | 0.873 | 0.040 | 0.065 | 0.012 | 0.010 | 0.001 | 0.001 |  |  |
| C15 (*m/z* 256) |  |  |  |  |  |  |  |  |  |
| Expt | 0.786 | 0.062 | 0.105 | 0.022 | 0.019 | 0.004 | 0.003 |  |  |
| Sim | 0.795 | 0.066 | 0.107 | 0.016 | 0.013 | 0.002 | 0.001 |  |  |
| C16 (*m/z* 270) |  |  |  |  |  |  |  |  |  |
| Expt | 0.847 | 0.041 | 0.071 | 0.019 | 0.016 | 0.004 | 0.003 |  |  |
| Sim | 0.842 | 0.047 | 0.078 | 0.016 | 0.014 | 0.002 | 0.001 |  |  |
| C17 (*m/z* 284) |  |  |  |  |  |  |  |  |  |
| Expt | 0.734 | 0.073 | 0.126 | 0.030 | 0.025 | 0.006 | 0.005 |  |  |
| Sim | 0.740 | 0.081 | 0.132 | 0.024 | 0.020 | 0.003 | 0.002 |  |  |
| C18 (*m/z 298*) |  |  |  |  |  |  |  |  |  |
| Expt | 0.916 | 0.020 | 0.038 | 0.011 | 0.009 | 0.004 | 0.002 |  |  |
| Sim | 0.908 | 0.026 | 0.044 | 0.010 | 0.009 | 0.002 | 0.001 |  |  |

**Table BV continued.**

| FAME Fragment | M0 | M1 | M2 | M3 | M4 | M5 | M6 | M7 | M8 |
| --- | --- | --- | --- | --- | --- | --- | --- | --- | --- |
| *[U-^13^C]Isoleucine* |  |  |  |  |  |  |  |  |  |
|  |  |  |  |  |  |  |  |  |  |
| C14 (*m/z* 242) |  |  |  |  |  |  |  |  |  |
| Expt | 0.933 | 0.004 | 0.055 | 0.001 | 0.006 | 0.000 | 0.001 |  |  |
| Sim | 0.933 | 0.006 | 0.055 | 0.001 | 0.005 | 0.000 | 0.000 |  |  |
| C15 (*m/z* 256) |  |  |  |  |  |  |  |  |  |
| Expt | 0.741 | 0.005 | 0.079 | 0.134 | 0.012 | 0.024 | 0.001 | 0.003 |  |
| Sim | 0.748 | 0.006 | 0.076 | 0.131 | 0.011 | 0.025 | 0.001 | 0.002 |  |
| C16 (*m/z* 270) |  |  |  |  |  |  |  |  |  |
| Expt | 0.918 | 0.005 | 0.065 | 0.002 | 0.009 | 0.000 | 0.001 |  |  |
| Sim | 0.916 | 0.007 | 0.068 | 0.002 | 0.008 | 0.000 | 0.001 |  |  |
| C17 (*m/z* 284) |  |  |  |  |  |  |  |  |  |
| Expt | 0.698 | 0.007 | 0.097 | 0.148 | 0.015 | 0.030 | 0.002 | 0.004 |  |
| Sim | 0.704 | 0.008 | 0.093 | 0.144 | 0.015 | 0.032 | 0.001 | 0.003 |  |
| C18 (*m/z 298*) |  |  |  |  |  |  |  |  |  |
| Expt | 0.958 | 0.004 | 0.033 | 0.000 | 0.004 | 0.000 | 0.001 |  |  |
| Sim | 0.950 | 0.004 | 0.039 | 0.001 | 0.005 | 0.000 | 0.000 |  |  |
|  |  |  |  |  |  |  |  |  |  |
| *[U-^13^C]Glutamine* |  |  |  |  |  |  |  |  |  |
|  |  |  |  |  |  |  |  |  |  |
| C14 (*m/z* 242) |  |  |  |  |  |  |  |  |  |
| Expt | 0.854 | 0.006 | 0.099 | 0.004 | 0.027 | 0.002 | 0.007 | 0.002 |  |
| Sim | 0.862 | 0.009 | 0.097 | 0.004 | 0.023 | 0.001 | 0.003 | 0.000 |  |
| C15 (*m/z* 256) |  |  |  |  |  |  |  |  |  |
| Expt | 0.786 | 0.009 | 0.151 | 0.006 | 0.038 | 0.001 | 0.008 | 0.000 |  |
| Sim | 0.777 | 0.016 | 0.163 | 0.006 | 0.033 | 0.001 | 0.004 | 0.000 |  |
| C16 (*m/z* 270) |  |  |  |  |  |  |  |  |  |
| Expt | 0.820 | 0.007 | 0.118 | 0.006 | 0.037 | 0.001 | 0.011 | 0.001 |  |
| Sim | 0.829 | 0.011 | 0.114 | 0.006 | 0.032 | 0.002 | 0.005 | 0.000 |  |
| C17 (*m/z* 284) |  |  |  |  |  |  |  |  |  |
| Expt | 0.720 | 0.013 | 0.185 | 0.006 | 0.053 | 0.002 | 0.016 | 0.000 | 0.005 |
| Sim | 0.718 | 0.019 | 0.198 | 0.009 | 0.048 | 0.002 | 0.006 | 0.000 | 0.001 |
| C18 (*m/z 298*) |  |  |  |  |  |  |  |  |  |
| Expt | 0.882 | 0.009 | 0.063 | 0.002 | 0.024 | 0.001 | 0.010 | 0.001 | 0.008 |
| Sim | 0.901 | 0.006 | 0.063 | 0.004 | 0.020 | 0.001 | 0.004 | 0.000 | 0.001 |

**Table BW.** Measured vs. simulated mass isotopomer distributions of fatty acids (FAME derivatives) for parallel labeling experiment #2 (SSR = 191.2, statistically accepted fit) with [U-^13^C]valine, [U-^13^C]leucine, [U-^13^C]isoleucine, and [U-^13^C]glutamine measured on day 7 (t=24h). Combined ISA model used for data fitting. Data was corrected for natural isotope abundances.

| FAME Fragment | M0 | M1 | M2 | M3 | M4 | M5 | M6 | M7 | M8 |
| --- | --- | --- | --- | --- | --- | --- | --- | --- | --- |
| *[U-^13^C]Valine* |  |  |  |  |  |  |  |  |  |
|  |  |  |  |  |  |  |  |  |  |
| C14 (*m/z* 242) |  |  |  |  |  |  |  |  |  |
| Expt | 0.998 | 0.002 | 0.001 | 0.000 | 0.000 | 0.000 |  |  |  |
| Sim | 1.000 | 0.000 | 0.000 | 0.000 | 0.000 | 0.000 |  |  |  |
| C15 (*m/z* 256) |  |  |  |  |  |  |  |  |  |
| Expt | 0.912 | 0.005 | 0.014 | 0.069 | 0.000 | 0.000 |  |  |  |
| Sim | 0.920 | 0.000 | 0.012 | 0.068 | 0.000 | 0.000 |  |  |  |
| C16 (*m/z* 270) |  |  |  |  |  |  |  |  |  |
| Expt | 0.998 | 0.001 | 0.001 | 0.000 | 0.000 | 0.000 |  |  |  |
| Sim | 1.000 | 0.000 | 0.000 | 0.000 | 0.000 | 0.000 |  |  |  |
| C17 (*m/z* 284) |  |  |  |  |  |  |  |  |  |
| Expt | 0.906 | 0.002 | 0.016 | 0.077 | 0.000 | 0.000 |  |  |  |
| Sim | 0.912 | 0.000 | 0.014 | 0.074 | 0.000 | 0.000 |  |  |  |
| C18 (*m/z 298*) |  |  |  |  |  |  |  |  |  |
| Expt | 1.000 | 0.000 | 0.000 | 0.000 | 0.000 | 0.000 |  |  |  |
| Sim | 1.000 | 0.000 | 0.000 | 0.000 | 0.000 | 0.000 |  |  |  |
|  |  |  |  |  |  |  |  |  |  |
| *[U-^13^C]Leucine* |  |  |  |  |  |  |  |  |  |
|  |  |  |  |  |  |  |  |  |  |
| C14 (*m/z* 242) |  |  |  |  |  |  |  |  |  |
| Expt | 0.874 | 0.039 | 0.060 | 0.014 | 0.011 | 0.002 | 0.001 |  |  |
| Sim | 0.867 | 0.044 | 0.066 | 0.012 | 0.009 | 0.001 | 0.001 |  |  |
| C15 (*m/z* 256) |  |  |  |  |  |  |  |  |  |
| Expt | 0.787 | 0.067 | 0.100 | 0.022 | 0.017 | 0.004 | 0.003 |  |  |
| Sim | 0.794 | 0.070 | 0.105 | 0.016 | 0.012 | 0.002 | 0.001 |  |  |
| C16 (*m/z* 270) |  |  |  |  |  |  |  |  |  |
| Expt | 0.843 | 0.046 | 0.071 | 0.018 | 0.015 | 0.004 | 0.003 |  |  |
| Sim | 0.841 | 0.050 | 0.076 | 0.016 | 0.013 | 0.002 | 0.001 |  |  |
| C17 (*m/z* 284) |  |  |  |  |  |  |  |  |  |
| Expt | 0.740 | 0.076 | 0.121 | 0.029 | 0.024 | 0.007 | 0.004 |  |  |
| Sim | 0.746 | 0.083 | 0.126 | 0.023 | 0.018 | 0.003 | 0.001 |  |  |
| C18 (*m/z 298*) |  |  |  |  |  |  |  |  |  |
| Expt | 0.913 | 0.020 | 0.041 | 0.011 | 0.010 | 0.003 | 0.002 |  |  |
| Sim | 0.911 | 0.027 | 0.041 | 0.010 | 0.008 | 0.002 | 0.001 |  |  |

**Table BW continued.**

| FAME Fragment | M0 | M1 | M2 | M3 | M4 | M5 | M6 | M7 | M8 |
| --- | --- | --- | --- | --- | --- | --- | --- | --- | --- |
| *[U-^13^C]Isoleucine* |  |  |  |  |  |  |  |  |  |
|  |  |  |  |  |  |  |  |  |  |
| C14 (*m/z* 242) |  |  |  |  |  |  |  |  |  |
| Expt | 0.930 | 0.007 | 0.054 | 0.001 | 0.007 | 0.000 | 0.000 |  |  |
| Sim | 0.930 | 0.007 | 0.056 | 0.001 | 0.005 | 0.000 | 0.000 |  |  |
| C15 (*m/z* 256) |  |  |  |  |  |  |  |  |  |
| Expt | 0.738 | 0.008 | 0.079 | 0.135 | 0.013 | 0.024 | 0.002 | 0.003 |  |
| Sim | 0.745 | 0.008 | 0.076 | 0.133 | 0.011 | 0.025 | 0.001 | 0.002 |  |
| C16 (*m/z* 270) |  |  |  |  |  |  |  |  |  |
| Expt | 0.919 | 0.006 | 0.064 | 0.002 | 0.009 | 0.000 | 0.001 |  |  |
| Sim | 0.915 | 0.008 | 0.067 | 0.002 | 0.007 | 0.000 | 0.001 |  |  |
| C17 (*m/z* 284) |  |  |  |  |  |  |  |  |  |
| Expt | 0.703 | 0.009 | 0.094 | 0.147 | 0.015 | 0.028 | 0.001 | 0.004 |  |
| Sim | 0.709 | 0.009 | 0.091 | 0.141 | 0.015 | 0.031 | 0.001 | 0.003 |  |
| C18 (*m/z 298*) |  |  |  |  |  |  |  |  |  |
| Expt | 0.960 | 0.000 | 0.034 | 0.001 | 0.006 | 0.000 | 0.000 |  |  |
| Sim | 0.952 | 0.005 | 0.037 | 0.001 | 0.005 | 0.000 | 0.000 |  |  |
|  |  |  |  |  |  |  |  |  |  |
| *[U-^13^C]Glutamine* |  |  |  |  |  |  |  |  |  |
|  |  |  |  |  |  |  |  |  |  |
| C14 (*m/z* 242) |  |  |  |  |  |  |  |  |  |
| Expt | 0.842 | 0.009 | 0.101 | 0.004 | 0.028 | 0.005 | 0.008 | 0.004 |  |
| Sim | 0.854 | 0.011 | 0.101 | 0.005 | 0.024 | 0.001 | 0.003 | 0.000 |  |
| C15 (*m/z* 256) |  |  |  |  |  |  |  |  |  |
| Expt | 0.776 | 0.014 | 0.149 | 0.007 | 0.043 | 0.003 | 0.009 | 0.000 |  |
| Sim | 0.774 | 0.018 | 0.163 | 0.007 | 0.033 | 0.001 | 0.003 | 0.000 |  |
| C16 (*m/z* 270) |  |  |  |  |  |  |  |  |  |
| Expt | 0.821 | 0.009 | 0.117 | 0.006 | 0.036 | 0.002 | 0.010 | 0.001 |  |
| Sim | 0.827 | 0.013 | 0.115 | 0.007 | 0.032 | 0.002 | 0.005 | 0.000 |  |
| C17 (*m/z* 284) |  |  |  |  |  |  |  |  |  |
| Expt | 0.727 | 0.016 | 0.182 | 0.006 | 0.050 | 0.002 | 0.013 | 0.001 | 0.005 |
| Sim | 0.722 | 0.021 | 0.192 | 0.010 | 0.046 | 0.002 | 0.006 | 0.000 | 0.001 |
| C18 (*m/z 298*) |  |  |  |  |  |  |  |  |  |
| Expt | 0.895 | 0.002 | 0.062 | 0.001 | 0.024 | 0.000 | 0.009 | 0.001 | 0.006 |
| Sim | 0.904 | 0.007 | 0.061 | 0.004 | 0.019 | 0.001 | 0.004 | 0.000 | 0.000 |

**Table BX.** Measured vs. simulated mass isotopomer distributions of fatty acids (FAME derivatives) for parallel labeling experiment #3 (SSR = 199.3, statistically accepted fit) with [U-^13^C]valine, [U-^13^C]leucine, [U-^13^C]isoleucine, and [U-^13^C]glutamine measured on day 7 (t=24h). Combined ISA model used for data fitting. Data was corrected for natural isotope abundances.

| FAME Fragment | M0 | M1 | M2 | M3 | M4 | M5 | M6 | M7 | M8 |
| --- | --- | --- | --- | --- | --- | --- | --- | --- | --- |
| *[U-^13^C]Valine* |  |  |  |  |  |  |  |  |  |
|  |  |  |  |  |  |  |  |  |  |
| C14 (*m/z* 242) |  |  |  |  |  |  |  |  |  |
| Expt | 0.998 | 0.000 | 0.002 | 0.000 | 0.000 | 0.000 |  |  |  |
| Sim | 1.000 | 0.000 | 0.000 | 0.000 | 0.000 | 0.000 |  |  |  |
| C15 (*m/z* 256) |  |  |  |  |  |  |  |  |  |
| Expt | 0.913 | 0.000 | 0.014 | 0.073 | 0.000 | 0.000 |  |  |  |
| Sim | 0.918 | 0.000 | 0.012 | 0.070 | 0.000 | 0.000 |  |  |  |
| C16 (*m/z* 270) |  |  |  |  |  |  |  |  |  |
| Expt | 0.998 | 0.000 | 0.002 | 0.000 | 0.000 | 0.000 |  |  |  |
| Sim | 1.000 | 0.000 | 0.000 | 0.000 | 0.000 | 0.000 |  |  |  |
| C17 (*m/z* 284) |  |  |  |  |  |  |  |  |  |
| Expt | 0.902 | 0.000 | 0.016 | 0.081 | 0.001 | 0.000 |  |  |  |
| Sim | 0.909 | 0.000 | 0.013 | 0.078 | 0.000 | 0.000 |  |  |  |
| C18 (*m/z 298*) |  |  |  |  |  |  |  |  |  |
| Expt | 0.997 | 0.000 | 0.003 | 0.000 | 0.000 | 0.000 |  |  |  |
| Sim | 1.000 | 0.000 | 0.000 | 0.000 | 0.000 | 0.000 |  |  |  |
|  |  |  |  |  |  |  |  |  |  |
| *[U-^13^C]Leucine* |  |  |  |  |  |  |  |  |  |
|  |  |  |  |  |  |  |  |  |  |
| C14 (*m/z* 242) |  |  |  |  |  |  |  |  |  |
| Expt | 0.868 | 0.039 | 0.065 | 0.013 | 0.011 | 0.003 | 0.001 |  |  |
| Sim | 0.863 | 0.043 | 0.071 | 0.012 | 0.010 | 0.001 | 0.001 |  |  |
| C15 (*m/z* 256) |  |  |  |  |  |  |  |  |  |
| Expt | 0.798 | 0.057 | 0.103 | 0.021 | 0.016 | 0.004 | 0.002 |  |  |
| Sim | 0.799 | 0.065 | 0.106 | 0.015 | 0.012 | 0.001 | 0.001 |  |  |
| C16 (*m/z* 270) |  |  |  |  |  |  |  |  |  |
| Expt | 0.842 | 0.041 | 0.076 | 0.019 | 0.015 | 0.004 | 0.003 |  |  |
| Sim | 0.838 | 0.049 | 0.081 | 0.016 | 0.013 | 0.002 | 0.001 |  |  |
| C17 (*m/z* 284) |  |  |  |  |  |  |  |  |  |
| Expt | 0.738 | 0.075 | 0.124 | 0.028 | 0.025 | 0.007 | 0.004 |  |  |
| Sim | 0.748 | 0.079 | 0.129 | 0.022 | 0.018 | 0.003 | 0.001 |  |  |
| C18 (*m/z 298*) |  |  |  |  |  |  |  |  |  |
| Expt | 0.904 | 0.027 | 0.043 | 0.011 | 0.011 | 0.004 | 0.001 |  |  |
| Sim | 0.905 | 0.028 | 0.046 | 0.010 | 0.009 | 0.002 | 0.001 |  |  |

**Table BX continued.**

| FAME Fragment | M0 | M1 | M2 | M3 | M4 | M5 | M6 | M7 | M8 |
| --- | --- | --- | --- | --- | --- | --- | --- | --- | --- |
| *[U-^13^C]Isoleucine* |  |  |  |  |  |  |  |  |  |
|  |  |  |  |  |  |  |  |  |  |
| C14 (*m/z* 242) |  |  |  |  |  |  |  |  |  |
| Expt | 0.928 | 0.005 | 0.059 | 0.001 | 0.007 | 0.000 | 0.001 |  |  |
| Sim | 0.927 | 0.005 | 0.061 | 0.001 | 0.006 | 0.000 | 0.000 |  |  |
| C15 (*m/z* 256) |  |  |  |  |  |  |  |  |  |
| Expt | 0.736 | 0.001 | 0.082 | 0.139 | 0.013 | 0.025 | 0.001 | 0.003 |  |
| Sim | 0.744 | 0.005 | 0.078 | 0.135 | 0.010 | 0.025 | 0.001 | 0.002 |  |
| C16 (*m/z* 270) |  |  |  |  |  |  |  |  |  |
| Expt | 0.917 | 0.001 | 0.070 | 0.002 | 0.009 | 0.000 | 0.001 |  |  |
| Sim | 0.913 | 0.006 | 0.072 | 0.001 | 0.008 | 0.000 | 0.001 |  |  |
| C17 (*m/z* 284) |  |  |  |  |  |  |  |  |  |
| Expt | 0.695 | 0.010 | 0.097 | 0.149 | 0.015 | 0.029 | 0.002 | 0.004 |  |
| Sim | 0.704 | 0.007 | 0.095 | 0.145 | 0.014 | 0.032 | 0.001 | 0.003 |  |
| C18 (*m/z 298*) |  |  |  |  |  |  |  |  |  |
| Expt | 0.957 | 0.001 | 0.036 | 0.000 | 0.005 | 0.000 | 0.000 |  |  |
| Sim | 0.948 | 0.004 | 0.042 | 0.001 | 0.005 | 0.000 | 0.000 |  |  |
|  |  |  |  |  |  |  |  |  |  |
| *[U-^13^C]Glutamine* |  |  |  |  |  |  |  |  |  |
|  |  |  |  |  |  |  |  |  |  |
| C14 (*m/z* 242) |  |  |  |  |  |  |  |  |  |
| Expt | 0.838 | 0.008 | 0.110 | 0.005 | 0.030 | 0.002 | 0.008 | 0.000 |  |
| Sim | 0.845 | 0.010 | 0.109 | 0.005 | 0.026 | 0.001 | 0.004 | 0.000 |  |
| C15 (*m/z* 256) |  |  |  |  |  |  |  |  |  |
| Expt | 0.778 | 0.007 | 0.159 | 0.006 | 0.039 | 0.001 | 0.010 | 0.000 |  |
| Sim | 0.773 | 0.016 | 0.167 | 0.006 | 0.033 | 0.001 | 0.004 | 0.000 |  |
| C16 (*m/z* 270) |  |  |  |  |  |  |  |  |  |
| Expt | 0.809 | 0.007 | 0.123 | 0.007 | 0.039 | 0.002 | 0.012 | 0.001 |  |
| Sim | 0.818 | 0.011 | 0.122 | 0.006 | 0.034 | 0.002 | 0.006 | 0.000 |  |
| C17 (*m/z* 284) |  |  |  |  |  |  |  |  |  |
| Expt | 0.718 | 0.016 | 0.183 | 0.006 | 0.055 | 0.002 | 0.015 | 0.001 | 0.006 |
| Sim | 0.716 | 0.019 | 0.200 | 0.009 | 0.048 | 0.002 | 0.006 | 0.000 | 0.001 |
| C18 (*m/z 298*) |  |  |  |  |  |  |  |  |  |
| Expt | 0.888 | 0.000 | 0.069 | 0.001 | 0.026 | 0.001 | 0.010 | 0.000 | 0.005 |
| Sim | 0.894 | 0.006 | 0.068 | 0.004 | 0.022 | 0.001 | 0.004 | 0.000 | 0.001 |

**Table BY.** Measured vs. simulated mass isotopomer distributions of fatty acids (FAME derivatives) for parallel labeling experiment #4 (SSR = 140.1, statistically accepted fit) with [U-^13^C]valine, [U-^13^C]leucine, [U-^13^C]isoleucine, and [U-^13^C]glutamine measured on day 7 (t=24h). Combined ISA model used for data fitting. Data was corrected for natural isotope abundances.

| FAME Fragment | M0 | M1 | M2 | M3 | M4 | M5 | M6 | M7 | M8 |
| --- | --- | --- | --- | --- | --- | --- | --- | --- | --- |
| *[U-^13^C]Valine* |  |  |  |  |  |  |  |  |  |
|  |  |  |  |  |  |  |  |  |  |
| C14 (*m/z* 242) |  |  |  |  |  |  |  |  |  |
| Expt | 0.999 | 0.000 | 0.001 | 0.000 | 0.000 | 0.000 |  |  |  |
| Sim | 1.000 | 0.000 | 0.000 | 0.000 | 0.000 | 0.000 |  |  |  |
| C15 (*m/z* 256) |  |  |  |  |  |  |  |  |  |
| Expt | 0.916 | 0.001 | 0.013 | 0.069 | 0.000 | 0.000 |  |  |  |
| Sim | 0.921 | 0.000 | 0.012 | 0.068 | 0.000 | 0.000 |  |  |  |
| C16 (*m/z* 270) |  |  |  |  |  |  |  |  |  |
| Expt | 0.999 | 0.000 | 0.001 | 0.000 | 0.000 | 0.000 |  |  |  |
| Sim | 1.000 | 0.000 | 0.000 | 0.000 | 0.000 | 0.000 |  |  |  |
| C17 (*m/z* 284) |  |  |  |  |  |  |  |  |  |
| Expt | 0.907 | 0.002 | 0.015 | 0.076 | 0.000 | 0.000 |  |  |  |
| Sim | 0.913 | 0.000 | 0.013 | 0.074 | 0.000 | 0.000 |  |  |  |
| C18 (*m/z 298*) |  |  |  |  |  |  |  |  |  |
| Expt | 0.999 | 0.000 | 0.000 | 0.001 | 0.000 | 0.000 |  |  |  |
| Sim | 1.000 | 0.000 | 0.000 | 0.000 | 0.000 | 0.000 |  |  |  |
|  |  |  |  |  |  |  |  |  |  |
| *[U-^13^C]Leucine* |  |  |  |  |  |  |  |  |  |
|  |  |  |  |  |  |  |  |  |  |
| C14 (*m/z* 242) |  |  |  |  |  |  |  |  |  |
| Expt | 0.857 | 0.043 | 0.067 | 0.016 | 0.012 | 0.003 | 0.002 |  |  |
| Sim | 0.856 | 0.046 | 0.071 | 0.014 | 0.011 | 0.002 | 0.001 |  |  |
| C15 (*m/z* 256) |  |  |  |  |  |  |  |  |  |
| Expt | 0.775 | 0.068 | 0.107 | 0.024 | 0.019 | 0.004 | 0.003 |  |  |
| Sim | 0.781 | 0.073 | 0.111 | 0.018 | 0.014 | 0.002 | 0.001 |  |  |
| C16 (*m/z* 270) |  |  |  |  |  |  |  |  |  |
| Expt | 0.824 | 0.050 | 0.079 | 0.021 | 0.017 | 0.005 | 0.003 |  |  |
| Sim | 0.824 | 0.054 | 0.083 | 0.019 | 0.015 | 0.003 | 0.002 |  |  |
| C17 (*m/z* 284) |  |  |  |  |  |  |  |  |  |
| Expt | 0.727 | 0.078 | 0.126 | 0.031 | 0.027 | 0.007 | 0.005 |  |  |
| Sim | 0.730 | 0.086 | 0.132 | 0.026 | 0.021 | 0.003 | 0.002 |  |  |
| C18 (*m/z 298*) |  |  |  |  |  |  |  |  |  |
| Expt | 0.907 | 0.025 | 0.040 | 0.012 | 0.010 | 0.004 | 0.002 |  |  |
| Sim | 0.902 | 0.029 | 0.045 | 0.012 | 0.010 | 0.002 | 0.001 |  |  |

**Table BY continued.**

| FAME Fragment | M0 | M1 | M2 | M3 | M4 | M5 | M6 | M7 | M8 |
| --- | --- | --- | --- | --- | --- | --- | --- | --- | --- |
| *[U-^13^C]Isoleucine* |  |  |  |  |  |  |  |  |  |
|  |  |  |  |  |  |  |  |  |  |
| C14 (*m/z* 242) |  |  |  |  |  |  |  |  |  |
| Expt | 0.927 | 0.008 | 0.057 | 0.001 | 0.007 | 0.000 | 0.001 |  |  |
| Sim | 0.926 | 0.009 | 0.058 | 0.002 | 0.006 | 0.000 | 0.000 |  |  |
| C15 (*m/z* 256) |  |  |  |  |  |  |  |  |  |
| Expt | 0.732 | 0.010 | 0.080 | 0.136 | 0.013 | 0.025 | 0.001 | 0.003 |  |
| Sim | 0.740 | 0.010 | 0.077 | 0.133 | 0.012 | 0.025 | 0.001 | 0.002 |  |
| C16 (*m/z* 270) |  |  |  |  |  |  |  |  |  |
| Expt | 0.909 | 0.010 | 0.069 | 0.002 | 0.009 | 0.000 | 0.001 |  |  |
| Sim | 0.908 | 0.011 | 0.070 | 0.002 | 0.008 | 0.000 | 0.001 |  |  |
| C17 (*m/z* 284) |  |  |  |  |  |  |  |  |  |
| Expt | 0.699 | 0.013 | 0.096 | 0.145 | 0.015 | 0.028 | 0.002 | 0.003 |  |
| Sim | 0.704 | 0.012 | 0.092 | 0.141 | 0.016 | 0.031 | 0.002 | 0.003 |  |
| C18 (*m/z 298*) |  |  |  |  |  |  |  |  |  |
| Expt | 0.954 | 0.004 | 0.035 | 0.001 | 0.005 | 0.000 | 0.001 |  |  |
| Sim | 0.947 | 0.006 | 0.039 | 0.002 | 0.005 | 0.000 | 0.000 |  |  |
|  |  |  |  |  |  |  |  |  |  |
| *[U-^13^C]Glutamine* |  |  |  |  |  |  |  |  |  |
|  |  |  |  |  |  |  |  |  |  |
| C14 (*m/z* 242) |  |  |  |  |  |  |  |  |  |
| Expt | 0.854 | 0.010 | 0.101 | 0.004 | 0.026 | 0.001 | 0.005 | 0.000 |  |
| Sim | 0.859 | 0.011 | 0.100 | 0.005 | 0.022 | 0.001 | 0.003 | 0.000 |  |
| C15 (*m/z* 256) |  |  |  |  |  |  |  |  |  |
| Expt | 0.792 | 0.014 | 0.148 | 0.005 | 0.034 | 0.001 | 0.007 | 0.000 |  |
| Sim | 0.785 | 0.017 | 0.158 | 0.006 | 0.029 | 0.001 | 0.003 | 0.000 |  |
| C16 (*m/z* 270) |  |  |  |  |  |  |  |  |  |
| Expt | 0.823 | 0.011 | 0.117 | 0.006 | 0.034 | 0.001 | 0.009 | 0.000 |  |
| Sim | 0.828 | 0.013 | 0.117 | 0.007 | 0.030 | 0.002 | 0.004 | 0.000 |  |
| C17 (*m/z* 284) |  |  |  |  |  |  |  |  |  |
| Expt | 0.736 | 0.018 | 0.177 | 0.006 | 0.047 | 0.001 | 0.012 | 0.001 | 0.004 |
| Sim | 0.736 | 0.020 | 0.186 | 0.009 | 0.041 | 0.002 | 0.005 | 0.000 | 0.000 |
| C18 (*m/z 298*) |  |  |  |  |  |  |  |  |  |
| Expt | 0.890 | 0.003 | 0.065 | 0.003 | 0.023 | 0.001 | 0.010 | 0.001 | 0.004 |
| Sim | 0.903 | 0.007 | 0.063 | 0.004 | 0.019 | 0.001 | 0.003 | 0.000 | 0.000 |
